# An Integrative Multi-Omics Analysis Based on Nomogram for Predicting Prostate Cancer Bone Metastasis Incidence

**DOI:** 10.1155/2022/8213723

**Published:** 2022-09-29

**Authors:** Jun Zhao, Rui Wang, Xiaoxin Sun, Kai Huang, Jiacheng Jin, Lan Lan, Yuli Jian, Zhongyang Xu, Haotian Wu, Shujing Wang, Jianbo Wang

**Affiliations:** ^1^Department of Urology, First Affiliated Hospital of Dalian Medical University, Dalian, Liaoning 116000, China; ^2^College of Integrative Medicine, Dalian Medical University, Dalian, Liaoning 116000, China; ^3^Department of Biochemistry, Institute of Glycobiology, Dalian Medical University, Dalian, Liaoning 116000, China

## Abstract

**Background:**

The most common site of prostate cancer metastasis is bone tissue with many recent studies having conducted genomic and clinical research regarding bone metastatic prostate cancer. However, further work is needed to better define those patients that are at an elevated risk of such metastasis.

**Methods:**

SEER and TCGA databases were searched to develop a nomogram for predicting prostate cancer bone metastasis.

**Results:**

Herein, we leveraged the Surveillance, Epidemiology, and End Results (SEER) database to construct a predictive nomogram capable of readily and accurately predicted the odds of bone metastasis in prostate cancer patients. This nomogram was utilized to assign patients with prostate cancer included in The Cancer Genome Atlas (TCGA) to cohorts at a high or low risk of bone metastasis (HRBM and LRBM, respectively). Comparisons of these LRBM and HRBM cohorts revealed marked differences in mutational landscapes between these patient cohorts, with increased frequencies of gene fusions, somatic copy number variations (CNVs), and single nucleotide variations (SNVs), particularly in the P53 gene, being evident in the HRBM cohort. We additionally identified lncRNAs, miRNAs, and mRNAs that were differentially expressed between these two patient cohorts and used them to construct a competing endogenous RNA (ceRNA) network. Moreover, three weighted gene co-expression network analysis (WGCNA) modules were constructed from the results of these analyses, with KIF14, MYH7, and COL10A1 being identified as hub genes within these modules. We further found immune response activity levels in the HRBM cohort to be elevated relative to that in the LRBM cohort, with single sample gene enrichment analysis (ssGSEA) scores for the immune checkpoint signature being increased in HRBM patient samples relative to those from LRBM patients.

**Conclusion:**

We successfully developed a nomogram capable of readily detecting patients with prostate cancer at an elevated risk of bone metastasis.

## 1. Introduction

Prostate cancer is the second most common form of cancer affecting males, with approximately 1.275 million new cases and 0.35 million deaths worldwide each year [1]. Prostate tumors most frequently metastasize to the bone, which is the site of metastasis in roughly 70% of cases [2]. Through the development of the large-scale clinical SEER database (https://seer.cancer.gov/) and the next-generation sequencing-based TCGA database (https://portal.gdc.cancer.gov/), important advances have been made in the current understanding of the genetic and clinical nature of prostate cancer.

To date, most studies regarding prostate cancer bone metastasis have focused on cases that have already progressed to this phase of disease [3, 4], whereas in clinical settings urologists primarily seek a reliable means of identifying patients likely to progress to bone metastatic disease. Most patients with prostate cancer do not harbor bone metastases when initially diagnosed, and it is thus critical to determine who is at an elevated risk of such metastases in order to guide appropriate preventative treatment where possible. In one prior multivariate logistic regression analysis-based model, patients who were married, lived in urban areas, exhibited lower levels of prostate-specific antigen (PSA), had undergone surgery, and had undergone radiation treatment exhibited lower odds ratios (ORs) for bone metastasis when analyzing SEER database data [5]. However, owing to uniform evaluative standards, some of these variables may be classified differently across datasets, particularly socioeconomic variables, thus constraining the overall utility of this model. As such, there is a clear need for the development of a simple, graphical nomogram capable of enabling precise clinical predictions based on relevant risk factors [6], with such nomograms having been used as predictive tools in prostate cancer and other diseases [7]. An appropriately constructed and calibrated nomogram would enable urologists to more reliably identify prostate cancer patients at an elevated risk of bone metastases and would be applicable to multiple different datasets, thus enabling integrated analyses of prostate cancer bone metastasis.

The Cancer Genome Atlas Research Network has leveraged data from the TCGA database to identify a range of genomic changes including gene fusions, mutations, and copy number variations (CNVs) associated with the incidence of primary prostate cancer [3]. One recent report utilized transcriptomic data from prostate cancer patients in the TCGA database to develop prognosis-related ceRNA networks [8]. Such networks incorporate miRNAs, target mRNAs, and lncRNAs that can competitively bind to specific miRNAs in a sequence-specific manner, thus modulating their ability to regulate downstream target gene expression [8]. The R WGCNA package allows users to conduct weighted correlation network analyses, enabling the identification of modules of genes that are highly correlated with one another and related to TCGA prostate cancer patient Gleason scores [9]. Moreover, ssGSEAs allow for analyses of gene set scores in a given sample, and they have previously been employed to demonstrate that immune environments differ between samples based on intratumoral infiltration by CD8+ T cells, regulatory T cells (Tregs), and helper T (Th) cells [10]. To date, no studies to our knowledge have conducted a comprehensive analysis of diverse sets of omics data via a bioinformatic approach to identify novel targets with potential therapeutic relevance in the context of preventing prostate cancer bone metastasis.

Herein, we leveraged the SEER database to develop a multivariate logistic regression-based nomogram capable of predicting the odds of bone metastasis in prostate cancer patients, with an optimal cutoff value being identified through receiver operating characteristic (ROC) curve analyses. This nomogram was then employed to stratify patients in the TCGA cohort into LRBM and HRBM groups, after which we compared the somatic mutational landscape between these two patient subsets. We additionally developed a competing endogenous RNA (ceRNA) network based on lncRNAs, miRNAs, and mRNAs that were differentially expressed between LRBM and HRBM samples, and we employed a WGCNA approach to construct three co-expression modules in which KIF14, MYH7, and COL10A1 were identified as central hub genes. Lastly, we found immune response activity levels to be elevated in the HRBM cohort as compared to the LRBM cohort. As such, we herein developed a nomogram amenable to broad clinical utilization as a tool for defining prostate cancer patients at a high risk of bone metastasis in clinical settings. Moreover, these results will serve as a foundation for future research efforts aimed at understanding the mechanisms governing prostate cancer bone metastasis and aiding in the identification of biomarkers or therapeutic targets associated with these destructive metastatic processes in prostate cancer patients.

## 2. Methods

### 2.1. Sample Selection

The SEER-18 Regs Research Data (release date: November 2019) were retrieved with SEER*∗*Stat v8.3.6 (https://seer.cancer.gov/seerstat/software/) (NCI, NIH, USA). Samples included in the present analysis were those meeting the following criteria: primary site = “Prostate,” with codes including ICD-O-3 Hist/behave, malignant = “8140/3: Adenocarcinoma,” and “NOS.” Patients were excluded if they exhibited unclear TNM staging according to the 6th edition AJCC criteria, unclear ages, PSA levels, or Gleason score. Overall, 281,550 SEER samples were included in the present analysis, with all prostate cancer patients being randomly assigned to a training and a validation cohort containing 70% and 30% of cases, respectively.

### 2.2. Study Variables

Patient clinicopathological characteristics including PSA levels, Gleason score, and TNM stage at the time of diagnosis were extracted from the SEER database. According to the 6th edition of AJCC criteria, patients with M1b stage disease were affected by bone metastases.

### 2.3. Nomogram Construction and Validation

Training data from the SEER database were utilized in univariate and multivariate analyses for nomogram construction using the R “glm” function. Univariate logistic model development was conducted by analyzing variables including age, T stage, N stage, PSA levels, primary Gleason scores, and secondary Gleason scores. Those variables that were significant in univariate analyses were then included in a multivariate logistic regression analysis, the results of which were utilized to construct a nomogram using the R “nomogram” function. Nomogram calibration was assessed with the “val.prob” function in the R “rms” package [11], with *P* > 0.05 being indicative of goodness of fit. The area under the ROC curve (AUC) was used to assess the ability of this nomogram to discriminate between patients with and without bone metastases using the R “pROC” package, with AUC values of 0.5 and 1.0, respectively, corresponding to an absence of discrimination and perfect discrimination. Youden's index was utilized to calculate the optimal cutoff threshold. This nomogram was also used to assess the odds of bone metastasis in the validation cohort, with both calibration and ROC curves for data in the validation group being utilized to assess nomogram predictive efficacy. The “roc.test” function in the “pROC” package was used for comparing ROC curves from the training and validation groups.

### 2.4. SNV Analysis

Prostate cancer SNV data, aggregated and masked with VarScan2, were obtained from the Genomic Data Commons (GDC) data portal and the TCGA (https://tcga-data.nci.nih.gov/tcga/) database. SNV in the VCF format was analyzed using the R “maftools” package [12]. The “mafcompare” function was utilized to compare SNVs between the LRBM and HRBM cohorts. Tumor mutational burden (TMB) was calculated for each TCGA sample, with TMB scores being defined as the total number of coding errors in somatic genes, insertions, deletions, and substitutions per million bases as follows: TMB score = number of variants/the length of exons (38 million). Perl scripts were utilized for TMB score calculation with the Java 8 platform.

### 2.5. CNV Analysis

The GDC portal was employed to download “Masked Copy Number Segment” data, with CNB segment means being transformed to gene copy numbers as follows: (copy number = 2 (1 + segment)). CNVs were transformed to yield discrete values as follows copy number <0.5 and $\underline{\geq }$0: −2 (loss of two copies), copy number <1.5 and $\underline{\geq }$0.5: −1 (loss of one copy), copy number $\underline{\leq }$2.5 and $\underline{\geq }$1.5 : 0 (no copy changes), copy number $\underline{\leq }$3.5 and >2.5 : 1 (single copy amplification), and copy number>3.5 : 2 (high-level copy number amplification). CNV frequencies were compared between cohorts using Pearson's chi-squared test, with *P* values being corrected using the Bonferroni approach. An adjusted *P* < 0.05 was the significance threshold.

### 2.6. Functional Enrichment Analyses

The R “ClusterProfiler” package was utilized to conduct GO and KEGG enrichment analyses, with an adjusted *P* < 0.05 as the significance threshold.

### 2.7. ceRNA Network Construction

The GDC data portal was utilized to download raw lncRNA, miRNA, and mRNA read data, after which genes that were differentially expressed between the HRBM and LRBM groups were identified with the R “DESeq2” package. DElncRNAs and DEmRNAs were selected based on an adjusted *P* < 0.05 and |FC| > 2, while DEmiRNAS were selected based on an adjusted *P* < 0.05 and |FC| > 1.5. The miRcode database (v 11: https://www.mircode.org/) was used to identify interactions between miRNAs and lncRNAs, with this predictive database including the complete Encyclopedia of DNA Elements (ENCODE)-annotated transcriptome [13]. DEmRNA targets of DEmiRNAs were identified using miRDB (v 5.0; https://mirdb.org), miRTarBase (v 7.0; https://mirtarbase.mbc.nctu.edu.tw/), and TargetScan (v 7.2; https://www.targetscan.org/vert_72/) [14–16]. Those interactions predicted by at least 2 databases were retained for analysis.

### 2.8. WGCNA

The R “WGCNA” package was utilized for co-expression network development based on mRNAs that were differentially expressed when comparing the LRBM and HRBM cohorts [17]. Adjacency matrices were transformed into a topological overlap matrix (TOM), with genes being separated into different modules based on TOM-based dissimilarity measures. The “pick Soft Threshold” function in this package was used to select a soft power threshold of 18 (scale-free *R*2 = 0.85), with a cut height of 0.8 and a minimum module size of 5 being utilized for key module identification. These co-expression modules were then prepared for visualization with the “exportNetworkToCytoscape” function and were visualized with a threshold of 0.2 using Cytoscape, which facilitates integrated biomolecular interaction network visualization [18]. Within these networks, mRNAs were visualized, and hub genes were identified based on the maximal clique centrality (MCC) using the cytoHubba plugin [19]. The functional roles of genes within each module were identified via GO and KEGG enrichment analyses, and protein-protein interaction (PPI) networks were developed using the online STRING database (https://string-db.org/).

### 2.9. Immune Signature Enrichment Analyses

To evaluate immune signature enrichment in different samples, 29 immune-related signatures were selected based on three published studies [20–22]. The “ssGSEA” function in the R “GSVA” package was then utilized for the calculation of ssGSEA value(xi) for each prostate cancer sample in the TCGA cohort, with final ssGSEA results being normalized as follows: (xi = (xi-xmin)/(xmax-xmin)), where xmin and xmax correspond, respectively, to the lowest and highest ssGSEA values for the analyzed prostate cancer samples. These scores were then compared between the HRBM and LRBM cohorts via the Mann–Whitney *U* tests.

### 2.10. Immune, Stromal, ESTIMATE, and Tumor Purity Score Calculation

The “estimateScore” function in the R “estimate” package was employed for the calculation of tumor purity, immune, stromal, and ESTIMATE scores in the LRBM and HRBM cohorts, with these scores then being compared via the Mann–Whitney *U* tests.

### 2.11. Statistical Analysis

R 3.5.0 was used for all statistical analyses, with a two-tailed *P* < 0.05 as the significance threshold.

## 3. Results

### 3.1. Bone Metastasis-Related Nomogram Construction

**Figure 1:**
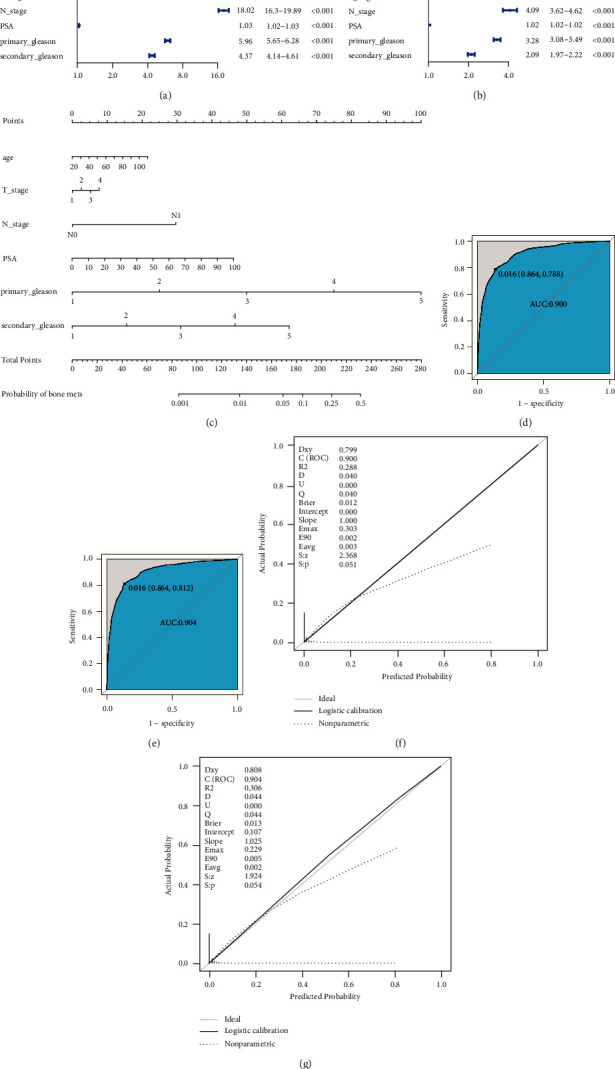
Construction of a nomogram capable of predicting prostate cancer patient bone metastasis. (a) Forest plot results corresponding to a univariate logistic regression model analysis of bone metastasis risk. (b) Forest plot results corresponding to a multivariate logistic regression model analysis of bone metastasis risk. The *x*-axis corresponds to the OR for bone metastasis. OR: odds ratio. CI: confidence interval. (c) A nomogram used to predict the odds of prostate cancer patient bone metastasis based on patient age, T_stage, N_stage, PSA, primary Gleason score, and secondary Gleason score. To use the nomogram, a straight line was drawn upwards from the appropriate point on each variable axis to the score axis, with the points for each of these predictors being summed together. The total sum score was then used to judge the odds of bone metastasis for that patient by drawing a line downwards. (d) ROC curves for the predictive nomogram in the training cohort (ROC curve AUC = 0.9; cutoff = 0.016; sensitivity = 0.864; specificity = 0.788). (e) ROC curves for the predictive nomogram in the validation cohort (ROC curve AUC = 0.904; cutoff = 0.016; sensitivity = 0.864; specificity = 0.812). (f) Calibration models for the predictive model when used to analyze the training cohort, with the actual and predicted probability being graphed against one another. (g) Calibration models for the predictive model when used to analyze the validation cohort. In the calibration curves, the reference line corresponds to perfect concordance between predicted and actual odds of bone metastasis.

To construct a nomogram capable of assessing the risk of bone metastasis in prostate cancer patients, we selected seven clinical variables including age, T stage, N stage, M stage, PSA, primary Gleason score, and secondary Gleason score that were shared in the SEER and TCGA databases. In total, the SEER database included 1,307,625 prostate cancer patients of which 102,675 were excluded due to an absence of necessary data or a failure to meet with study inclusion criteria, while 281,550 patients were incorporated into this analysis and randomly separated into a training set (*n* = 197,090) and a validation set (*n* = 84,460). Patient characteristics are summarized in Supplementary Data 1-1. In logistic regression analyses of the patients in the training cohort, age, T stage, N stage, PSA, primary Gleason score, and secondary Gleason score were all identified as independent predictors of the risk of bone metastasis in individuals with prostate cancer in univariate and multivariate analyses (Figures 1(a) and 1(b)). These six variables were thus included in a predictive nomogram capable of gauging the odds of bone metastasis (Figure 1(c)). This nomogram exhibited high discriminative potential, with an AUC value of 0.9 (Figure 1(d)). ROC curves corresponding to our nomogram revealed that at the optimal cutoff value of 0.016, the sensitivity and specificity values were 0.788 and 0.864, respectively. We then utilized this nomogram to predict the odds of bone metastasis for SEER database patients included in our validation cohort, with an AUC value of 0.904 at the optimal cutoff of 0.016, thus supporting the results obtained from the training cohort (Figure 1(e)). Moreover, consistency between the training and validation cohorts was assessed via Delong's test, demonstrating that these two ROC curves were highly consistent with one another (Supplementary Data 1 and 2). When calibrating our nomogram, we found that it was able to accurately gauge the odds of bone metastasis in our training samples (Figure 1(f)). In the validation cohort, however, we obtained *P* < 0.05, indicating that it was able to accurately predict the odds of bone metastasis in this group (Figure 1(g)). All of these data suggested that our nomogram was able to differentiate between low- and high-risk bone metastasis patients.

### 3.2. Nomogram-Based TCGA Data Analysis and Patient Grouping

Next, prostate cancer patient data from the TCGA database were obtained from the GDC data portal. Of the 500 patients in this database, 379 exhibited complete data pertaining to patient age, PSA levels, Gleason scores, and TNM stage at the time of initial diagnosis. Using the nomogram constructed above, the odds of bone marrow metastasis were gauged for these patients (cutoff = 0.016). All 10 TCGA cases exhibiting bone metastases were assigned to the HRBM group (Supplementary Data 1–3).

### 3.3. Comparison of SNVs in the LRBM and HRBM Cohorts

**Figure 2:**
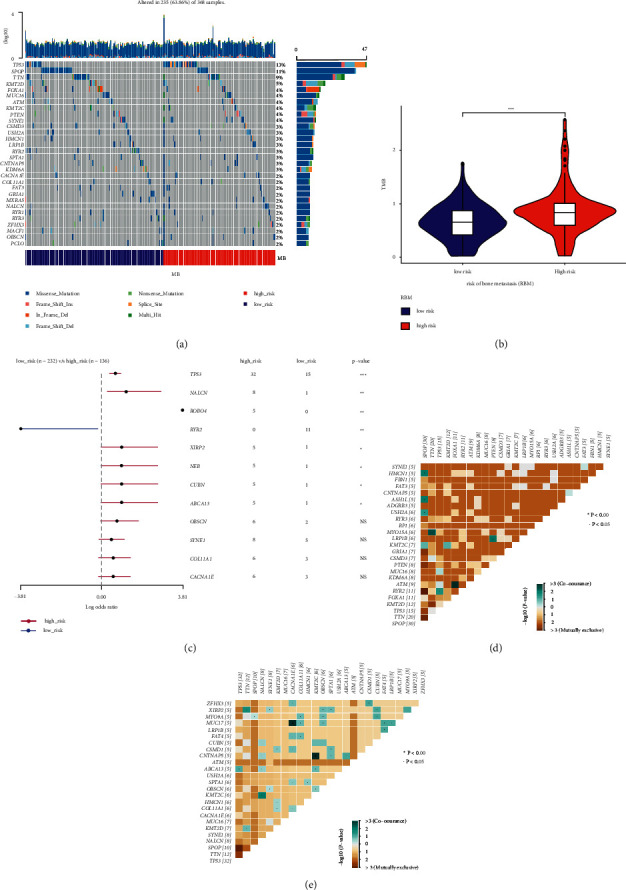
SNV comparisons in patients at a low and high risk of bone metastasis. (a) Mutational landscape profile for prostate cancer patient samples, with the waterfall plot being used to show mutational information for each gene, while colors with specific annotations along the bottom of the plot denote specific types of mutations. Mutational burden is shown in a bar plot above the legend. MB, metastasis of bone. (b) TMB value for the LRBM and HRBM cohorts. Violin plots represent TMB values as dots, with a box plot being present within this violin plot. ^*∗*^*P* < 0.05, ^*∗∗*^*P* < 0.01, ^*∗∗∗*^*P* < 0.001; two-sided Mann–Whitney *U* test. (c) Differentially mutated genes were compared between the LRBM and HRBM cohorts with the “mafCompare” function in the R “maftools” package. ^*∗*^*P* < 0.05, ^*∗∗*^*P* < 0.01, ^*∗∗∗*^*P* < 0.001. (d-e) Coincident and exclusive associations among mutated genes within the (d) LRBM and (e) HRBM cohorts.

Next, waterfall plots were generated for 368 prostate cancer patients in the TCGA cohort, revealing that a majority of genes exhibited low-frequency SNV mutation rates, with SNV frequency being highest for the TP53 gene (Figure 2(a)). We assess SNV frequencies in the overall (Figure S1(a)), LRBM (Figure S1(b)), and HRBM (Figure S1(c)) cohorts, revealing that missense mutations were the most common mutation type, with SNPs being more common than insertions/deletions and with *C* > *T* mutations being the most common SNV type in these patients. Median numbers of variants per sample differed between these cohorts, with a higher number of median variants per sample in the HRBM cohort (median: 22) (Figure S1(c)) as compared to the overall (median: 19) (Figure S1(a)) and LRBM (median: 17) cohorts (Figure S1(b)). We then calculated TMB scores in these patients (Supplementary Data 2), with these scores being higher in HRBM samples relative to LRBM samples (Figure 2(b)). Differences in the top 30 high-frequency SNVs in the LRBM (Figure S1(d)) and HRBM (Figure S1(e)) cohorts reveal that 8 genes exhibited significant differences in SNV frequencies between these two patient groups (TP53, NALCN, ROBO4, RYR2, XIRP2, NEB, CUBN, ABCA13) (Figure 2(c)). Mutational plots revealed that TP53 mutations in the LRBM group co-occurred with RYR2 mutations (Figure 2(d)), while in HRBM patients they co-occurred with ABCA13 mutations (Figure 2(e)). These data suggested that prostate cancer patients harboring high SNV frequencies are more likely to develop bone metastases, particularly among individuals with elevated p53 mutational frequencies.

### 3.4. Differences in CNVs and Fusion Genes in the LRBM and HRBM Cohorts

**Figure 3:**
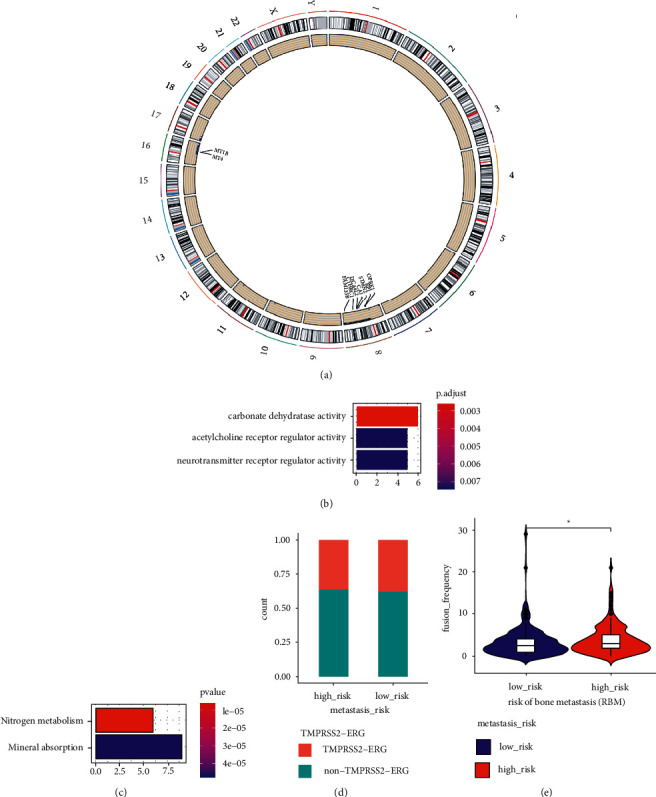
Comparisons of CNV and gene fusion frequencies in the HRBM and LRBM cohorts. (a) CNVs with different mutational frequencies in the LRBM and HRBM patient cohorts, with the inner circle presenting a scatter plot of the 676 CNVs that were differentially frequent in these two cohorts, and the outer circle corresponding to the locations of these genes on specific chromosomes. Those genes harboring CNVs that were also significantly differentially expressed between these two cohorts are marked within the circle. (b-c) GO (b) and KEGG (c) analyses of genes containing CNVs at different frequencies in the LRBM and HRBM cohorts, with enriched terms being shown on the left and bar plots on the right corresponding to the number of genes associated with the indicated term. Bar coloration is based on the *P* value for the corresponding term. (d) Frequencies of TMPRSS2-ERG gene fusions in the LRBM and HRBM cohorts, with the *y*-axis corresponding to the TMPRSS2-ERG gene fusion proportion. (e) Frequencies of gene fusions in the LRBM and HRBM cohorts. Violin plots show gene fusion frequencies in individual samples as dots, with boxplots being drawn within violin plots. ^*∗*^*P* < 0.05, ^*∗∗*^*P* < 0.01, ^*∗∗∗*^*P* < 0.001; two-sided Mann–Whitney *U* test.

When CNV frequencies were compared between the HRBM and LRBM cohorts, 676 genes harboring CNVs at different frequencies between these groups were identified (Figure 3(a)), Supplementary Data 3). These genes were enriched in GO terms including carbonate dehydratase activity, acetylcholine receptor regulator activity, and neurotransmitter receptor regulator activity (Figure 3(b)), as well as KEGG terms such as nitrogen metabolism and mineral absorption (Figure 3(c)). The Tumor Fusion Gene Data Portal (https://www.tumorfusions.org/) [23] was additionally used to assess gene fusion data, revealing comparable frequencies of TMPRSS2-ERG, which is the most frequent form of gene fusion in prostate cancer, in both patient cohorts (Figure 3(d)). Overall, the average gene fusion frequencies in the HRBM cohort were increased as compared to the LRBM cohort (Figure 3(e)). Together, these results suggested that HRBM patients exhibit increased heterogeneity as compared to the LRBM cohort.

### 3.5. Generation of a Bone Metastasis-Related ceRNA Network

**Figure 4:**
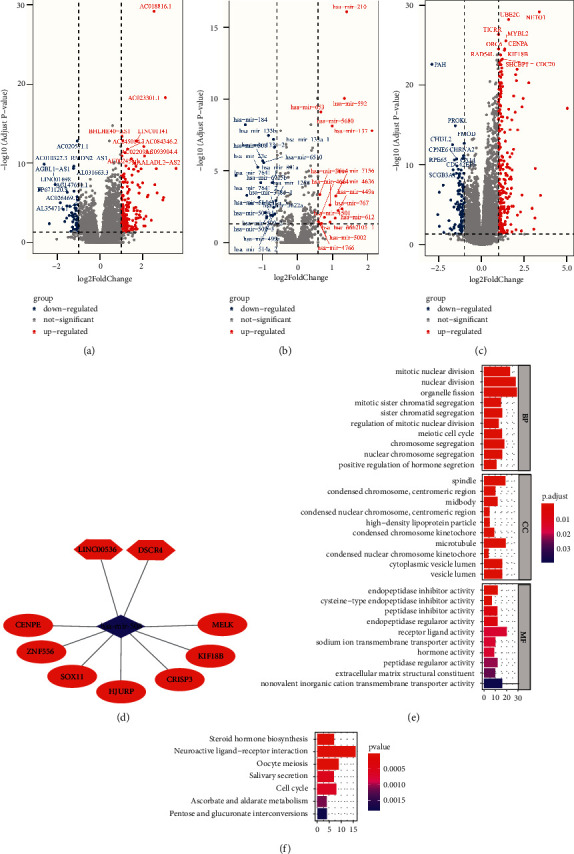
Generation of a bone metastasis-related ceRNA network. (a-c) Differentially expressed lncRNAs (a), miRNAs (b), and mRNAs (c) identified when comparing the LRBM and HRBM cohorts. The top 10 upregulated and downregulated genes are shown for each category, with horizontal lines corresponding to an adjusted P value of 0.05. (a, c) Vertical lines correspond to a log2 (fold change) at −1 and 1. (b) Vertical lines correspond to a log2 (fold change) at −0.58 and 0.58. (d) A ceRNA network was generated in which lncRNAs, miRNAs, and mRNAs were represented by hexagons, rhombuses, and ovals, respectively. (e) Enriched GO terms associated with mRNAs differentially expressed between the LRBM and HRBM cohorts, with the top 10 terms in each of three categories being shown. BP: biological processes. CC: cell component. MF: molecular function. (f) KEGG enrichment analyses for mRNAs differentially expressed in the HRBM and LRBM cohorts. The brown module was composed of five genes that were upregulated in HRBM patient samples, among which COL10A1 was the hub gene (Figure 5(h)). These five genes were enriched for GO terms including extracellular matrix structural constituent, extracellular matrix structural constituent conferring tensile strength, heparin binding, glycosaminoglycan binding, and sulfur compound binding (Figure 5(i)), as well as the protein digestion and absorption of KEGG pathway (Figure 5(j)). In the brown module PPI network, COL11A1 and COL10A1 were predicted to interact (Figure S2(e)).

The R “DESeq2” package was used to identify 201 DElncRNAs (Figure 4(a), Supplementary Data 4-1), 38 DEmiRNAs (Figure 4(b), Supplementary Data 4-2), and 358 DEmRNAs (Figure 4(c), Supplementary Data 4-3) when comparing the LRBM and HRBM cohorts. The miRcode database was used to identify two DEmiRNAs predicted to interact with four lncRNAs. However, of these interactions, both hsa-mir-137 and its predicted binding partners (LINC00536 and DSCR8) were upregulated in the HRBM cohort, in contrast with their predicted regulatory relationship given that lncRNAs are generally expected to downregulate miRNAs. These interactions were thus omitted from our network. The resultant ceRNA network incorporated one DEmiRNA (hsa-mir-508) and two DElncRNAs (LINC00536 and DSCR4) (Figure 4(d)). Next, the miRDB, miRTarBase, and TargetScan databases were used to identify seven DEmRNAs that were expressed at higher levels in HRBM samples relative to LRBM samples that were predicted to be targets of hsa-mir-508 (Figure 4(d)).

As these mRNAs accounted for only a small fraction of the overall DEmRNAs identified in this study, we conducted GO and KEGG analyses of all such DEmRNAs, revealing them to be primarily associated with mitosis-related GO terms (Figure 4(e)), hormone activity, and extracellular matrix structural constituents, which are closely associated with the risk of bone metastasis (Figure 4(e)). In KEGG pathway analyses, these DEmRNAs were associated with the steroid hormone biosynthesis, oocyte meiosis, and cell cycle pathways (Figure 4(f)). These genes and related functional networks may thus promote the incidence of prostate cancer bone metastasis.

### 3.6. Identification of Gene Co-Expression Modules Related to Bone Metastasis Risk

**Figure 5:**
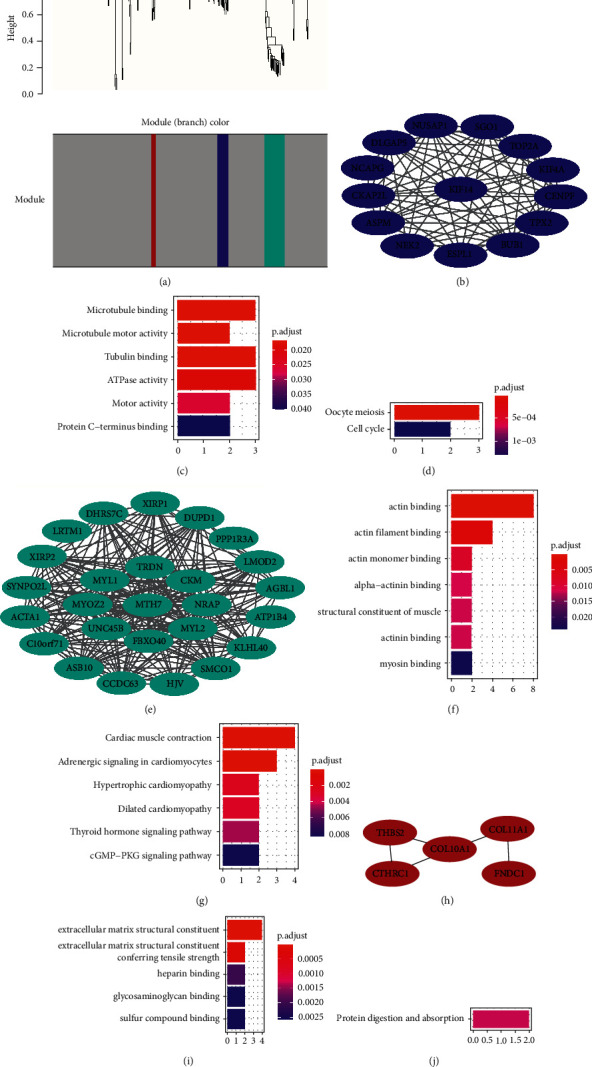
Bone metastasis-related gene module identification. (a) A dendrogram generated via the clustering of dissimilarity based on consensus topological overlap with corresponding modules being shown based on the colored rows corresponding to modules containing genes that were highly connected with one another. (b) Blue module co-expression network, with the hub gene in the network center. (c) GO analysis of genes in the blue module. (d) KEGG analysis of genes in the blue module. (e) Turquoise module co-expression network, with the hub gene in the network center. (f) GO analysis of genes in the turquoise module. (g) KEGG analysis of genes in the turquoise module. (h) Brown module co-expression network, with the hub gene in the network center. (i) GO analysis of genes in the brown module. (j) KEGG analysis of genes in the brown module.

Next, we generated WGCNA co-expression modules for the 358 DEmRNAs identified when comparing the LRBM and HRBM cohorts. When screening at a soft power value of 18 to reflect co-expression network scale-free topology (Figure S2(a), S2(b)), we were able to establish four separate co-expression modules that were colored blue, turquoise, brown, and grey, with the latter containing all genes that did not fit into the three former modules (Figure 5(a), Supplementary Data 5).

The blue module consisted of 15 genes that were upregulated in HRBM samples relative to LRBM samples, with KIF14 being identified as the hub gene in this module using the CytoHubba MCC method (Figure 5(b)). GO analyses revealed these blue module genes to be enriched in terms associated with microtubule binding, microtubule motor activity, tubulin binding, ATPase activity, motor activity, and protein C-terminal (Figure 5(c)), as well as KEGG terms including oocyte meiosis and cell cycle (Figure 5(d)).

We also generated a PPI network incorporating all of these genes (Figure S2(c)). The turquoise module consisted of 26 genes that were downregulated in HRBM samples relative to LRBM samples, with MYH7 being identified as the hub gene within this module (Figure 5(e)). Enrichment analyses revealed these genes to be enriched for GO terms including actin binding, actin filament binding, actin monomer binding, alpha-actin binding, structural constituent of muscle, actin binding, and myosin binding (Figure 5(f)) and for KEGG terms such as cardiac muscle contraction, adrenergic signaling in cardiomyocytes, hypertrophic cardiomyopathy, thyroid hormone signaling pathway, and the cGMP-PKG signaling pathway (Figure 5(g)). In the PPI network constructed based on these genes, MYH7 was predicted to interact with certain other genes from this module (Figure S2(d)).

### 3.7. The Relationship between the Immune Microenvironment and the Odds of Bone Metastasis

**Figure 6:**
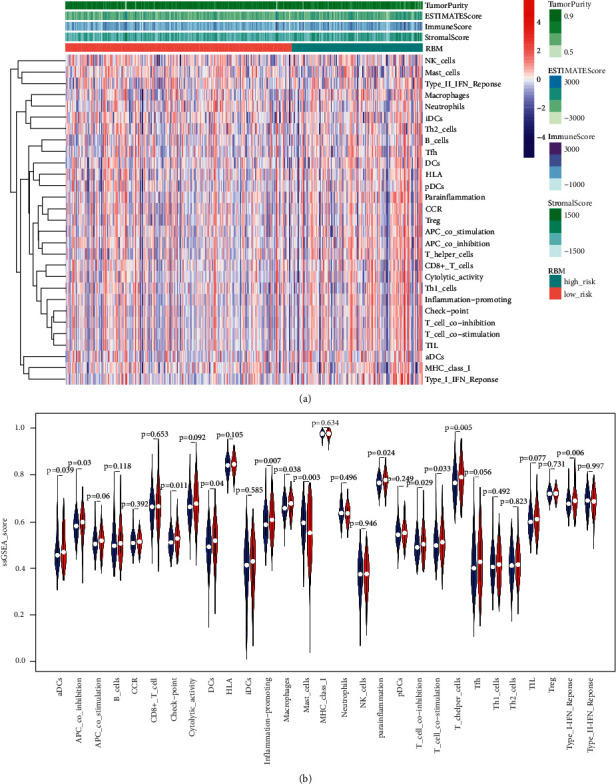
Relationship between immunological microenvironment and the odds of bone metastasis. (a) A hierarchical clustering analysis of 367 TCGA prostate cancer samples based on 29 immune-related gene sets. Tumor purity, ESTIMATE, immune, and stromal scores were determined using ESTIMATE. RBM: risk of bone metastasis. (b) ssGSEA score comparisons in the LRBM and HRBM cohorts for 29 immune-related gene sets. ^*∗*^*P* < 0.05, ^*∗∗*^*P* < 0.01, ^*∗∗∗*^*P* < 0.001; two-sided Mann–Whitney *U* test.

To evaluate the differences in the immune microenvironment in prostate cancer patients as a function of bone metastasis risk, we evaluated 29 immunological signatures (Supplementary Data 6-1) identified in another previous report [21]. Based on these compiled signatures, we assessed the enrichment of particular immune cells, pathways, or function types within prostate cancer samples in the TCGA database using an ssGSEA algorithm (Figure 6(a)). We found the ssGSEA scores for aDCs, APC_co_inhibition, checkpoint, DCs, inflammation-promoting, macrophages, para-inflammation, *T*_cell_co-inhibition, *T*_cell_co-stimulation, *T*_helper_cells, and TypeI_IFN_reponse to be higher in the HRBM cohort as compared to the LRBM cohort, whereas the mast cell ssGSEA score exhibited the opposite trend (Figure 6(b)). We also calculated immune, stromal, tumor purity, and ESTIMATE scores for these cohorts to gauge differences in the immune microenvironment between low- and high-risk patients (Supplementary Data 6-2) and revealed the stromal (Figure S3(a)), immune (Figure S3(b)), and ESTIMATE (Figure S3(c)) scores to be increased in HRBM patients relative to LRBM patients (Figure S3(d)), while the opposite trend was evident for tumor purity scores. These data suggested that HRBM samples exhibit enhanced immune system activation. When we explored differences in immune checkpoint gene expression between these cohorts, we found several such genes to be upregulated in HRBM patient samples including BTLA, CD276, CD70, CD80, CD86, HAVCR2, HHLA2, ICOS, IDO1, IDO2, LAIR1, LGALS9, NRP1, TIGIT, TNFRSF18, TNFRSF25, TNFRSF4, TNFRSF8, TNFRSF9, and TNFSF18 (Figure S3(e)). Together, these data indicated that HRBM patient immune activity was increased overall as compared to LRBM patients, with a pronounced upregulation of checkpoint gene expression in these high-risk patients.

## 4. Discussion

### 4.1. Bone Tissue Is the Most Common Site of Prostate Cancer

Metastasis [2]. While medical advances have improved prognostic outcomes in prostate cancer patients, bone metastases are still relatively common among patients with this form of malignancy [1]. As such, there is clear value in the construction of a model capable of predicting bone marrow metastasis and to explore genomic differences between individuals at low or high risk of bone metastasis in order to enable urologists to more accurately prevent and treat these metastases.

In prior reports, age, T stage, N stage, M stage, PSA, primary Gleason score, and secondary Gleason score are closely related to prostate cancer prognosis [24]. In univariate and multivariate logistic regression analyses, we confirmed that these variables were independent predictors of prostate cancer patient bone metastasis that were then used for nomogram construction. Our nomogram was not only accurately calibrated, although it exhibited excellent discriminatory capabilities, with an AUC of 0.9 [25]. Predictive results in the validation cohort of patients confirmed that we were able to accurately stratify patients into low-risk and high-risk cohorts based on the optimal cutoff value.

Prostate tumors frequently exhibit many low-frequency SNVs [3], and as such, we were only able to detect eight genes exhibiting differences in SNV frequencies when comparing the HRBM and LRBM patient cohorts. The gene most frequently harboring SNVs in prostate cancer patients is TP53 [3], and we further found the frequency of TP53 SNVs to be elevated in the HRBM cohort as compared to the LRBM cohort. The P53 protein plays essential roles in regulating transcription, metabolic changes, and cell cycle arrest [26]. Genomic instability is a hallmark of prostate cancer, resulting in high rates of detectable CNVs [27] and gene rearrangements [28]. We further found the HRBM cohort to exhibit an elevated CNV frequency as compared to the LRBM cohort. While no significant differences in TMPRSS2-ERG gene fusion frequency were observed when comparing the two patient cohorts, we did observe elevated gene fusion mutation rates in the HRBM group relative to the LRBM group. These differences are consistent with the higher levels of increased heterogeneity in HRBM patients relative to LRBM patients.

We additionally assessed posttranscriptional regulatory mechanisms involved in the onset of bone metastases. Through ceRNA-based competitive regulation of miRNAs, lncRNAs can regulate genes at the posttranscriptional level to contribute to prostate cancer bone metastases [29]. In the ceRNA network constructed in this study, we found hsa-mir-508 to be downregulated in HRBM samples relative to LRBM samples, whereas LINC00536 and DSCR4 were lncRNAs exhibiting the opposite trend. Likewise, the mRNAs encoding ENPF, ZNF556, SOX11, HJURP, CRISP3, KIF18B, and MELK were upregulated in HRBM samples relative to LRBM samples. Prior studies have indicated that hsa-mir-508 acts to suppress gastric cancer metastasis [30]. LINC00536 has been reported to drive bladder cancer progression and worse patient outcomes [31]. Moreover, a recent analysis indicated that DSCR4 suppresses human choriocarcinoma cell migration and invasion [32]. CENPF is related to cellular proliferation and mitotic activity, driving prostate cancer metastasis [33], while HJURP is involved in regulating the centromeric deposition of CENPF and controls cell cycle progression [34]. In one report, the loss of p53 was linked to the upregulation of both CENPF and HJURP [35]. SOX11 [36], CRISP3 [37], KIF18B [38], and MELK [39] expression levels have also been found to be positively correlated with poor prostate cancer patient prognostic outcomes, while ZNF556 has been associated with poor colon cancer patient prognosis [40]. Overall, these findings confirmed that the ceRNA network developed herein may offer insight into the likelihood that a given prostate tumor is likely to metastasize to the bone.

In one recent analysis, 9 DEGs and significant gene modules associated with CRPC phenotypes were identified, with a WGCNA approach being further utilized for the selection of three key hub genes [41]. We similarly leveraged a WGCNA approach to develop three co-expression networks associated with bone metastasis. PPI network analyses revealed genes in the blue module to exhibit functional correlations with one another, while GO analyses suggested that these blue module genes were microtubule-related. As essential components of the cytoskeleton, microtubules can control mitosis, the trafficking of organelles and vesicles, and the migration of cells [42]. Microtubule inhibitors have been reported to suppress the growth of metastatic prostate cancer [43]. Additional KEGG analyses revealed these blue module genes to be associated with oocyte meiosis and the cell cycle, additionally confirming the relationship between these genes and prostate cancer cell proliferative activity. The microtubule motor protein KIF14 [44] has also been reportedly linked to poor patient outcomes and disease progression in individuals with prostate cancer [45]. We found KIF14 to exhibit the highest connectivity, serving as a hub gene within the blue module. When we similarly conducted GO enrichment analyses of the turquoise module, we found the genes therein to be primarily related to actin binding. Recent work suggests that actin cytoskeletal remodeling can inhibit prostate cancer metastasis and invasion [46]. The turquoise module hub gene MYH7 has been reported to be involved in myosin-actin interactions [47]. Four of the five genes in the brown module were associated with the extracellular matrix. Prostate cancer cells must modify the primary tumor site ECM to escape this site and must similarly modify the ECM at the site of metastasis to facilitate metastatic growth and adaptation [48]. COL10A1, which was the brown module hub gene, is a component of the ECM that is known to facilitate lung adenocarcinoma metastasis [49]. Together, these data indicated that these three hub genes and the associated co-expression modules support bone metastasis via three different mechanisms, offering new insights for future study of this form of metastasis in individuals with prostate cancer.

For patients with inoperable bone metastases, immunotherapy may be one of the limited hopes. However, bone is a special immune site with a unique immunosuppressive microenvironment [50]. We found that samples in the HRBM group exhibited a significantly higher TMB as compared to samples in the LRBM group. Moreover, HRBM patient samples exhibited higher stromal, immune, and ESTIMATE scores but lower tumor purity as compared to LRBM samples. These results suggested that HRBM samples exhibit increased immune activity. Consistently, prior evidence has shown that a higher TMB is conducive to neoantigen formation, thus rendering tumors more immunogenic, thereby improving clinical responses to immunotherapeutic intervention [51]. In 2010, the FDA approved the first DC-based cancer vaccine, Sipuleucel-T, as a treatment for patients diagnosed with minimally symptomatic mCRPC, utilizing DCs loaded with PAP to generate specific anti-PAP responses to control tumor growth in these patients [52]. We observed increases in DCs and aDCs within samples from HRBM patients, suggesting that these individuals may be more responsive to DC-based vaccination. We additionally compared differences in immune components between these two patient cohorts, revealing an increased immune checkpoint signature in the HRBM group. Notably, a prior phase 3 trial testing a monoclonal CTLA-4-blocking antibody failed to improve OS in mCRPC patients [53], and the efficacy of PD-1 blockade has also been limited in this oncogenic context owing to the relatively low rates of PD-L1 expression relative to those observed for other cancer types [54]. Moreover, we detected no differences in PD-L1 or CTLA-4 expression when comparing the HRBM and LRBM groups, although other immune checkpoint molecules including TIGIT, IDO1, and CD274 were upregulated in HRBM samples as compared to LRBM samples, suggesting that they may be viable targets for immunotherapeutic intervention.

## 5. Conclusions

Herein, we used the SEER database to construct an easy-to-use nomogram capable of accurately gauging the odds of bone metastasis in prostate cancer patients. Comparisons of patients in the HRBM and LRBM cohorts revealed clear differences in mutational landscapes between these groups, with HRBM patients exhibiting higher frequencies of gene fusions, CNVs, and SNVs (particularly in the P53 gene) relative to LRBM patients. Moreover, we constructed a ceRNA network based on the lncRNAs, miRNAs, and mRNAs differentially expressed between these two patient cohorts, and we extracted three WGCNA co-expression modules. In so doing, we were able to identify KIF14, MYH7, and COL10A1 as hub genes within these modules. Lastly, we detected a higher degree of activity associated with immune responses in the HRBM group relative to the LRBM group, with immune checkpoint ssGSEA signature scores being higher in the HRBM cohort as compared to the LRBM cohort. These results have the potential to guide the prevention and targeted treatment of prostate cancer-associated bone metastases, in addition to providing a tool for screening for HRBM prostate cancer patients.

## Data Availability

The TCGA genetic mutation data and transcriptome data can be downloaded from the Genomic Data Commons (GDC) data portal (https://tcga-data.nci.nih.gov/tcga/). The SEER clinical data can be downloaded from SEER database (https://seer.cancer.gov/). The more bioinformatic analysis is included within the supplementary information files.

## Supplementary Materials

Supplementary Materials Figure S1: associated with Figure 2. Figure S2: associated with Figure 5. Figure S3: associated with Figure 6. Data 1-1: SEER data baseline characteristics of patient with prostate cancer; Data 1-2: train group ROC and validation ROC, Delong's test; Data 1-3: clinical features of TCGA samples and predictive results. Data 2: TMB scores of TCGA samples. Data 3: genes with different frequencies of CNVs between LRBM and HRBM cohorts. Data 4-1: DElncRNAs; Data 4-2: DEmiRNAs; Data 4-3: DEmRNAs. Data 5: gene module. Data 6-1: 29 immune microenvironment signatures; Data-2: ssGSEA score of immune microenvironment signature; Data 6-3: stromal score, immune score, estimate score, and tumor purity of TCGA samples.
