# Supplementary material for: An Integrative Multi-Omics Analysis Based on Nomogram for Predicting Prostate Cancer Bone Metastasis Incidence
Source: Genet Res (Camb). 2022 Sep 29;2022:8213723. doi: 10.1155/2022/8213723 (PMC9537037; doi:10.1155/2022/8213723)
Supplement: Supplementary Materials — Figure S1: associated with Figure 2. Figure S2: associated with Figure 5. Figure S3: associated with Figure 6. Data 1-1: SEER data baseline characteristics of patient with prostate cancer; Data 1-2: train group ROC and validation ROC, Delong's test; Data 1-3: clinical features of TCGA samples and predictive results. Data 2: TMB scores of TCGA samples. Data 3: genes with different frequencies of CNVs between LRBM and HRBM cohorts. Data 4-1: DElncRNAs; Data 4-2: DEmiRNAs; Data 4-3: DEmRNAs. Data 5: gene module. Data 6-1: 29 immune microenvironment signatures; Data-2: ssGSEA score of immune microenvironment signature; Data 6-3: stromal score, immune score, estimate score, and tumor purity of TCGA samples. [file 8213723.f1.zip › 8213723.f1/supplementary data1 (1).pdf]

Supplementary Data 1-1: SEER data baseline characteristics of patient with prostate cancer									
**SEER data baseline characteristics of patient with prostate cancer**									
	**The total cohort**			**The train cohort**			**The validation cohort**		
		**Bone metastases**			**Bone metastases**			**Bone metastases**	
	**Total**	**Yes**	**No**	**Total**	**Yes**	**No**	**Total**	**Yes**	**No**
**Age**									
**Median**	66	71	66	66	71	66	66	71	66
**Range**	20-106	35-99	20-106	20-106	35-99	20-106	31-99	39-98	31-99
**PSA(ng/ml)**									
**Mean**	21.65	63.4	21.05	21.65	63.07	21.06	21.63	64.14	21.01
**Primary gleason grade**									
**1**	246	0	246	186	0	186	60	0	60
**2**	2882	10	2872	2002	4	1998	880	6	874
**3**	211535	786	210749	148082	555	147527	63453	231	63222
**4**	59926	2385	57541	42012	1656	40356	17914	729	17185
**5**	6961	804	6157	4808	543	4265	2153	261	1892
**Secondary gleason grade**									
**1**	117	0	117	81	0	81	36	0	36
**2**	3266	4	3262	2319	1	2318	947	3	944
**3**	160840	836	160004	112452	589	111863	48388	247	48141
**4**	100552	1686	98866	70533	1165	69368	30019	521	29498
**5**	16775	1459	15316	11705	1003	10702	5070	456	4614
**T stage**									
**T1**	106325	1228	105097	74387	846	73541	31938	382	31556
**T2**	149834	1743	148091	104967	1229	103738	44867	514	44353
**T3**	22598	478	22120	15820	337	15483	6778	141	6637
**T4**	2793	536	2257	1916	346	1570	877	190	687
**N stage**									
**N0**	276818	3139	273679	193743	2187	191556	83075	952	82123
**N1**	4732	846	3886	3347	571	2776	1385	275	1110
**Bone mets**									
**Yes**	3985	3985	0	2758	2758	0	1227	1227	0
**No**	277565	0	277565	194332	0	194332	83233	0	83233

Supplementary Data 1-2:trian group roc and validation roc，Delong’s test

data: trian group roc and validation roc, Delong’s test
D = -0.76576, df = 160270, p-value = 0.4438
alternative hypothesis: true difference in AUC is not equal to 0
sample estimates:

AUC of training group data	AUC of validattion group data
89.96380	90.38788

Supplementary Data 1-3:clinical features of TCGA samples and predictive results										
	**ID**	**primary_gleason**	**secondary_gleason**	**PSA**	**N_stage**	**T_stage**	**age**	**probability of bone metastasis**	**risk of bone metastasis**	**actually bone metastasis**
	tcga-kk-a7b4	4	5	0.6	N1	3	65	0.092531	high_risk	YES
	tcga-ch-5751	5	5	0.01	N1	4	68	0.278363	high_risk	YES
	tcga-ej-5518	4	5	0.1	N0	4	67	0.027717	high_risk	YES
	tcga-ej-5525	4	5	3	N0	2	67	0.02321	high_risk	YES
	tcga-g9-a9s0	4	4	10.87	N1	3	54	0.051137	high_risk	YES
	tcga-hi-7168	4	5	0.3	N0	3	63	0.023621	high_risk	YES
	tcga-kk-a8i4	4	3	0.2	N1	3	65	0.022602	high_risk	YES
	tcga-kk-a8ii	4	5	39.8	N0	3	62	0.053847	high_risk	YES
	tcga-vp-a878	4	5	0.1	N1	2	59	0.076885	high_risk	YES
	tcga-yl-a9wy	5	4	1.8	N1	3	58	0.131496	high_risk	YES
	tcga-ch-5753	4	5	4.53	N1	3	71	0.106369	high_risk	NO
	tcga-c	4	5	0.	N0	3	6	0.023747	high_risk	NO

	h-5772			03			4			
	tcga-ej-a65j	4	5	0.1	N0	3	63	0.02352	high_risk	NO
	tcga-hc-7821	4	4	0.01	N1	3	55	0.041183	high_risk	NO
	tcga-kk-a7b3	4	5	0.1	N0	3	63	0.02352	high_risk	NO
	tcga-2a-a8vt	4	5	0.05	N1	4	48	0.085776	high_risk	NO
	tcga-2a-a8w3	4	5	0.36	N0	3	70	0.025568	high_risk	NO
	tcga-ch-5741	4	5	0.02	N1	3	57	0.084158	high_risk	NO
	tcga-ch-5754	4	5	37.36	N1	3	65	0.185979	high_risk	NO
	tcga-ch-5761	5	4	8.49	N0	3	61	0.042454	high_risk	NO
	tcga-ch-5766	4	3	13.9	N1	3	56	0.027414	high_risk	NO
	tcga-ch-5769	5	4	11.16	N0	3	49	0.03938	high_risk	NO
	tcga-ch-5788	4	3	3.58	N1	3	69	0.025406	high_risk	NO
	tcga-ch-5792	4	5	11.49	N0	3	58	0.028382	high_risk	NO
	tcga-ej-5495	5	3	0.13	N1	3	68	0.072574	high_risk	NO
	tcga-ej-5504	4	3	1	N1	3	66	0.023251	high_risk	NO
	tcga-ej-5506	5	3	0.1	N1	3	67	0.071766	high_risk	NO
	tcga-ej-5507	4	5	0.1	N1	3	54	0.081688	high_risk	NO

	tcga-ej-5514	4	5	0.1	N0	2	66	0.021568	high_risk	NO
	tcga-ej-5519	4	4	0.2	N1	3	65	0.046116	high_risk	NO
	tcga-ej-5524	4	5	0.1	N0	3	57	0.021997	high_risk	NO
	tcga-ej-5526	4	4	0.1	N1	3	56	0.041715	high_risk	NO
	tcga-ej-8469	4	5	3.1	N0	3	47	0.020982	high_risk	NO
	tcga-ej-a46f	4	4	19.8	N1	3	58	0.064217	high_risk	NO
	tcga-ej-a65b	4	5	0.01	N0	3	55	0.02147	high_risk	NO
	tcga-ej-a65f	4	5	0.8	N0	3	59	0.022834	high_risk	NO
	tcga-ej-a6ra	4	4	0.652	N1	3	70	0.049154	high_risk	NO
	tcga-ej-a7nm	4	5	0.1	N1	3	65	0.091614	high_risk	NO
	tcga-ej-a7nn	4	3	0.3	N1	3	62	0.021905	high_risk	NO
	tcga-fc-a5ob	4	5	5.22	N0	3	53	0.023479	high_risk	NO
	tcga-g9-6356	4	5	0.13	N0	3	60	0.02276	high_risk	NO
	tcga-g9-6367	4	5	0.42	N0	3	61	0.023159	high_risk	NO
	tcga-g9-6499	4	5	0.11	N0	3	67	0.024596	high_risk	NO
	tcga-g9-7521	4	4	0.06	N1	3	53	0.040333	high_risk	NO
	tcga-g9-7523	5	5	0.03	N0	3	45	0.060271	high_risk	NO
	tcga-g9-a9s4	4	4	0.03	N1	3	62	0.044474	high_risk	NO
	tcga-h	4	5	4.	N0	3	5	0.023174	high_risk	NO

	c-7213			09			4			
	tcga-hc-a8cy	5	4	0.9	N1	3	63	0.135816	high_risk	NO
	tcga-hc-a9te	5	4	0.064	N0	3	65	0.03714	high_risk	NO
	tcga-hc-a9th	5	4	0.38	N0	3	59	0.035001	high_risk	NO
	tcga-hi-7171	5	4	0.7	N0	3	57	0.034471	high_risk	NO
	tcga-j4-aatz	4	5	0	N0	3	66	0.024267	high_risk	NO
	tcga-j9-a52b	5	4	0.01	N0	3	63	0.036291	high_risk	NO
	tcga-j9-a52e	4	5	0.01	N0	3	65	0.024003	high_risk	NO
	tcga-j9-a8cm	5	4	0.55	N1	3	66	0.138964	high_risk	NO
	tcga-kc-a4br	4	5	0	N1	3	76	0.10241	high_risk	NO
	tcga-kc-a4bv	4	5	0.53	N0	3	66	0.024544	high_risk	NO
	tcga-kk-a59v	4	5	0.1	N0	3	64	0.023783	high_risk	NO
	tcga-kk-a59y	4	5	0.1	N1	3	57	0.084293	high_risk	NO
	tcga-kk-a5a1	4	5	0.1	N0	3	72	0.025998	high_risk	NO
	tcga-kk-a6e0	4	5	0.3	N0	3	59	0.02259	high_risk	NO
	tcga-kk-a6e1	4	5	0.1	N1	3	58	0.085178	high_risk	NO
	tcga-kk-a6e6	4	5	0.1	N0	3	69	0.025145	high_risk	NO

	tcga-kk-a6e7	5	4	0.1	N1	3	47	0.113986	high_risk	NO
	tcga-kk-a6e8	4	5	0.1	N0	2	69	0.022302	high_risk	NO
	tcga-kk-a7au	4	5	0	N1	3	63	0.089553	high_risk	NO
	tcga-kk-a7aw	4	3	0.8	N1	3	57	0.020939	high_risk	NO
	tcga-kk-a7b0	4	5	0.1	N0	3	68	0.024866	high_risk	NO
	tcga-kk-a7b2	4	5	0.3	N1	3	68	0.09488	high_risk	NO
	tcga-kk-a8i7	4	5	0.1	N0	3	55	0.021511	high_risk	NO
	tcga-kk-a8i8	4	5	0.1	N0	3	70	0.025426	high_risk	NO
	tcga-kk-a8ia	4	5	0.1	N1	3	69	0.095485	high_risk	NO
	tcga-kk-a8ic	4	5	0.9	N0	3	55	0.021884	high_risk	NO
	tcga-kk-a8id	5	4	0.1	N1	3	71	0.144707	high_risk	NO
	tcga-kk-a8ij	4	3	0.1	N1	3	60	0.021329	high_risk	NO
	tcga-kk-a8ik	4	5	0	N0	2	56	0.019245	high_risk	NO
	tcga-kk-a8il	4	5	0.1	N1	3	65	0.091614	high_risk	NO
	tcga-m7-a723	4	3	0.1	N1	3	55	0.02017	high_risk	NO
	tcga-mg-aamc	4	5	0.01	N0	3	60	0.022702	high_risk	NO
	tcga-v1-a8wv	4	5	0.01	N1	3	53	0.080689	high_risk	NO
	tcga-v	4	5	0.	N1	3	6	0.086826	high_risk	NO

	1-a8w w			01 5			0			
	tcga-v 1-a9o 5	5	4	3. 26	N1	4	6 5	0.160705	high_risk	NO
	tcga-v 1-a9o 7	5	4	9. 69	N0	3	6 1	0.043538	high_risk	NO
	tcga-v 1-a9o a	4	5	0. 01	N1	3	6 2	0.088644	high_risk	NO
	tcga-v 1-a9ol	5	4	0. 01	N0	3	6 6	0.037507	high_risk	NO
	tcga-v 1-a9o y	4	3	0. 09 9	N1	3	5 8	0.020857	high_risk	NO
	tcga-v 1-a9z 7	5	4	28 .1	N0	3	5 5	0.059858	high_risk	NO
	tcga-v 1-a9z 8	5	4	0. 01	N0	3	6 0	0.035112	high_risk	NO
	tcga-v 1-a9z 9	4	5	0. 01 5	N0	2	6 4	0.021053	high_risk	NO
	tcga-v 1-a9zg	4	5	0. 01 5	N0	2	6 5	0.021289	high_risk	NO
	tcga-v 1-a9zi	4	5	3. 11	N0	3	6 9	0.026816	high_risk	NO
	tcga-v n-a88 k	4	4	0	N1	3	5 9	0.043015	high_risk	NO
	tcga-v p-a87 2	5	3	0. 01	N0	3	6 1	0.017301	high_risk	NO
	tcga-v p-a87 9	4	5	0. 01	N0	2	7 1	0.022761	high_risk	NO
	tcga-v p-a87 d	4	5	0. 2	N1	3	5 4	0.081852	high_risk	NO
	tcga-v p-a87	4	5	0. 01	N0	3	7 7	0.02743	high_risk	NO

	h									
	tcga-vp-aa1n	5	4	0.01	N0	3	71	0.039623	high_risk	NO
	tcga-x4-a8kq	4	5	0.25	N0	3	66	0.024397	high_risk	NO
	tcga-xk-aaiv	5	5	0.32	N1	3	64	0.247067	high_risk	NO
	tcga-xk-aaiw	5	4	0.09	N0	3	79	0.04332	high_risk	NO
	tcga-xq-a8tb	4	5	0	N0	3	67	0.024539	high_risk	NO
	tcga-yl-a8hk	4	5	1	N0	3	60	0.023189	high_risk	NO
	tcga-yl-a8hl	4	5	0.03	N1	3	58	0.085059	high_risk	NO
	tcga-yl-a8hm	4	5	0.03	N0	3	66	0.024282	high_risk	NO
	tcga-yl-a8s8	4	5	1.36	N0	3	69	0.025831	high_risk	NO
	tcga-yl-a8s9	4	5	0.03	N0	3	64	0.023747	high_risk	NO
	tcga-yl-a8sb	4	5	0.23	N0	3	63	0.023585	high_risk	NO
	tcga-yl-a8sc	4	5	0.65	N0	3	66	0.024607	high_risk	NO
	tcga-yl-a8si	4	5	0.48	N0	3	69	0.02535	high_risk	NO
	tcga-yl-a8sj	4	5	0.24	N0	3	60	0.022814	high_risk	NO
	tcga-yl-a8sk	4	5	0.03	N1	3	68	0.094372	high_risk	NO
	tcga-yl-a8sl	4	4	0	N1	3	74	0.05064	high_risk	NO
	tcga-yl-a8sq	4	5	1.1	N0	3	62	0.023763	high_risk	NO
	tcga-yl-a8sr	4	5	0.06	N0	3	64	0.023763	high_risk	NO
	tcga-yl-a9wh	4	5	0.03	N1	3	68	0.094372	high_risk	NO
	tcga-yl-a9wi	4	5	0.03	N1	3	64	0.090542	high_risk	NO
	tcga-yl	4	4	3.	N1	3	4	0.040265	high_risk	NO

	-a9wj			1			7			
	tcga-yl-a9wk	4	5	15.6	N0	3	64	0.033101	high_risk	NO
	tcga-yl-a9wl	4	5	6.4	N0	3	59	0.025743	high_risk	NO
	tcga-yl-a9wx	4	5	0.76	N1	3	69	0.096743	high_risk	NO
	tcga-zg-a8qz	4	5	0.01	N1	3	66	0.092402	high_risk	NO
	tcga-zg-a9l1	4	5	0.07	N1	3	66	0.092513	high_risk	NO
	tcga-zg-a9l2	4	5	0.01	N0	3	70	0.025377	high_risk	NO
	tcga-zg-a9l4	5	4	0.03	N0	3	61	0.035516	high_risk	NO
	tcga-zg-a9l6	5	4	0.36	N0	3	65	0.037373	high_risk	NO
	tcga-zg-a9lb	5	4	0.02	N0	3	72	0.040068	high_risk	NO
	tcga-zg-a9lm	4	5	0	N1	3	72	0.098289	high_risk	NO
	tcga-zg-a9ln	5	4	7.42	N1	3	58	0.146233	high_risk	NO
	tcga-zg-a9ls	5	4	0.03	N1	3	64	0.134917	high_risk	NO
	tcga-zg-a9lu	5	4	0.7	N0	3	68	0.038903	high_risk	NO
	tcga-zg-a9ly	4	5	0.01	N1	3	60	0.086817	high_risk	NO
	tcga-zg-a9lz	4	5	2.19	N0	3	66	0.025431	high_risk	NO
	tcga-zg-a9m4	5	4	0.95	N1	3	66	0.140017	high_risk	NO
	tcga-zg-a9mc	5	4	31.85	N1	3	70	0.251366	high_risk	NO
	tcga-zg-a9n3	5	4	0.1	N1	3	74	0.148996	high_risk	NO
	tcga-zg-a9nd	5	4	0.08	N0	3	56	0.033648	high_risk	NO

	tcga-zg-a9ni	5	4	0.03	N0	3	73	0.040518	high_risk	NO
	tcga-ch-5763	3	4	0.1	N0	3	67	0.003668	low_risk	NO
	tcga-ch-5764	4	3	13.95	N0	3	66	0.007669	low_risk	NO
	tcga-ch-5790	3	4	0.1	N0	2	64	0.003137	low_risk	NO
	tcga-ch-5791	3	4	0.18	N0	3	72	0.00389	low_risk	NO
	tcga-ej-5542	3	4	0.1	N0	3	60	0.003388	low_risk	NO
	tcga-ej-7123	3	4	0.1	N0	2	60	0.002997	low_risk	NO
	tcga-ej-7312	4	3	0.1	N0	3	58	0.005178	low_risk	NO
	tcga-ej-7325	3	4	0.1	N0	3	63	0.003505	low_risk	NO
	tcga-ej-7791	3	4	0.1	N0	2	67	0.003246	low_risk	NO
	tcga-ej-a46b	4	4	0.1	N0	3	67	0.011915	low_risk	NO
	tcga-ej-a46g	4	4	0.1	N0	3	72	0.012606	low_risk	NO
	tcga-g9-6364	3	4	0.03	N0	3	73	0.003921	low_risk	NO
	tcga-hc-7748	3	3	0.1	N0	2	60	0.001436	low_risk	NO
	tcga-hc-a76w	3	4	0.01	N0	3	73	0.00392	low_risk	NO
	tcga-kc-a7fa	3	4	0	N0	3	64	0.003538	low_risk	NO
	tcga-qu-a6im	3	4	0.1	N0	2	60	0.002997	low_risk	NO
	tcga-xk-aaj3	4	4	0.09	N0	3	56	0.010522	low_risk	NO

	tcga-xk-aak1	4	3	0.09	N0	2	63	0.004849	low_risk	NO
	tcga-2a-a8vl	3	3	0.05	N0	2	51	0.001294	low_risk	NO
	tcga-2a-a8vv	3	3	0.05	N0	2	52	0.001309	low_risk	NO
	tcga-2a-a8vx	4	4	0.05	N0	3	71	0.012451	low_risk	NO
	tcga-2a-a8w1	4	3	0.05	N0	3	55	0.004999	low_risk	NO
	tcga-2a-aayu	3	3	0.05	N0	3	56	0.001549	low_risk	NO
	tcga-4l-aa1f	4	4	3.5	N0	3	65	0.012541	low_risk	NO
	tcga-ch-5737	4	3	14.69	N0	2	73	0.007468	low_risk	NO
	tcga-ch-5739	3	4	0.02	N0	3	66	0.003621	low_risk	NO
	tcga-ch-5740	3	4	1.6	N0	2	57	0.002993	low_risk	NO
	tcga-ch-5743	3	4	0.1	N0	2	67	0.003246	low_risk	NO
	tcga-ch-5744	4	3	9.63	N0	2	65	0.006108	low_risk	NO
	tcga-ch-5745	3	4	4.3	N0	3	68	0.004067	low_risk	NO
	tcga-ch-5746	3	4	0.1	N0	2	57	0.002897	low_risk	NO
	tcga-ch-5750	3	4	0.02	N0	2	72	0.00343	low_risk	NO
	tcga-ch-5752	4	4	0.02	N0	3	67	0.011895	low_risk	NO
	tcga-c	4	3	0.	N0	3	6	0.005286	low_risk	NO

	h-5762			01			0			
	tcga-ch-5765	3	4	0.02	N0	3	56	0.003231	low_risk	NO
	tcga-ch-5767	4	3	0.04	N0	2	67	0.005069	low_risk	NO
	tcga-ch-5768	2	4	0.1	N0	3	73	0.001202	low_risk	NO
	tcga-ch-5771	4	3	0.03	N0	2	63	0.004842	low_risk	NO
	tcga-ch-5789	3	4	0.02	N0	3	62	0.00346	low_risk	NO
	tcga-ch-5794	3	4	0.01	N0	2	66	0.003203	low_risk	NO
	tcga-ej-5496	3	4	0.1	N0	2	60	0.002997	low_risk	NO
	tcga-ej-5497	3	4	0.1	N0	2	48	0.002615	low_risk	NO
	tcga-ej-5498	4	3	0.1	N0	2	56	0.004479	low_risk	NO
	tcga-ej-5499	4	3	0.1	N0	3	61	0.005357	low_risk	NO
	tcga-ej-5501	4	3	0.1	N0	3	56	0.005062	low_risk	NO
	tcga-ej-5502	4	3	0.1	N0	2	51	0.004232	low_risk	NO
	tcga-ej-5503	5	3	0.1	N0	3	50	0.015321	low_risk	NO
	tcga-ej-5505	4	3	0.1	N0	2	58	0.004582	low_risk	NO
	tcga-ej-5508	3	4	0.1	N0	3	65	0.003586	low_risk	NO
	tcga-ej-5509	3	4	0.1	N0	2	64	0.003137	low_risk	NO
	tcga-ej-5510	4	3	0.1	N0	2	49	0.004137	low_risk	NO
	tcga-ej-5511	3	4	0.04	N0	3	56	0.003233	low_risk	NO

	tcga-ej-5512	3	4	0	N0	2	47	0.002579	low_risk	NO
	tcga-ej-5515	3	4	0.1	N0	3	61	0.003426	low_risk	NO
	tcga-ej-5516	3	4	0.12	N0	3	49	0.002991	low_risk	NO
	tcga-ej-5517	3	3	0.1	N0	2	56	0.001372	low_risk	NO
	tcga-ej-5521	3	4	0.1	N0	3	64	0.003545	low_risk	NO
	tcga-ej-5522	3	4	0.1	N0	2	52	0.002736	low_risk	NO
	tcga-ej-5527	4	3	0.1	N0	3	69	0.005867	low_risk	NO
	tcga-ej-5530	3	4	0.1	N0	2	61	0.003032	low_risk	NO
	tcga-ej-5531	4	3	0.1	N0	3	62	0.005418	low_risk	NO
	tcga-ej-5532	3	4	0.1	N0	3	58	0.003312	low_risk	NO
	tcga-ej-7115	4	3	0.1	N0	3	66	0.00567	low_risk	NO
	tcga-ej-7125	3	4	0.1	N0	2	45	0.002527	low_risk	NO
	tcga-ej-7218	3	4	0.1	N0	2	72	0.003436	low_risk	NO
	tcga-ej-7314	4	3	0.1	N0	3	62	0.005418	low_risk	NO
	tcga-ej-7315	4	3	0.1	N0	3	69	0.005867	low_risk	NO
	tcga-ej-7317	3	4	0.1	N0	2	71	0.003397	low_risk	NO
	tcga-ej-7321	3	3	0.1	N0	3	58	0.001587	low_risk	NO
	tcga-ej-7327	3	4	0.1	N0	3	62	0.003466	low_risk	NO
	tcga-ej-7330	4	3	0.1	N0	3	69	0.005867	low_risk	NO
	tcga-ej-7331	3	4	0.8	N0	2	64	0.003185	low_risk	NO
	tcga-ej-7781	3	4	0.1	N0	2	66	0.003209	low_risk	NO
	tcga-ej-7782	4	4	0.1	N0	2	71	0.01104	low_risk	NO

	tcga-ej-7783	4	3	0.7	N0	3	70	0.006012	low_risk	NO
	tcga-ej-7784	4	3	0.1	N0	2	64	0.004905	low_risk	NO
	tcga-ej-7785	3	4	0.1	N0	3	55	0.0032	low_risk	NO
	tcga-ej-7786	3	4	0.1	N0	2	62	0.003066	low_risk	NO
	tcga-ej-7788	3	4	0.2	N0	3	54	0.003171	low_risk	NO
	tcga-ej-7792	3	4	0.13	N0	2	53	0.00277	low_risk	NO
	tcga-ej-7794	3	4	0.1	N0	2	67	0.003246	low_risk	NO
	tcga-ej-7797	3	4	0.1	N0	2	54	0.002799	low_risk	NO
	tcga-ej-8468	4	4	0.1	N0	3	64	0.011519	low_risk	NO
	tcga-ej-8472	4	4	0.4	N0	3	63	0.011464	low_risk	NO
	tcga-ej-8474	4	4	0.01	N0	3	70	0.012301	low_risk	NO
	tcga-ej-a46d	3	5	0.03	N0	2	53	0.00576	low_risk	NO
	tcga-ej-a46h	3	4	0.1	N0	2	60	0.002997	low_risk	NO
	tcga-ej-a46i	3	4	0.1	N0	3	58	0.003312	low_risk	NO
	tcga-ej-a65d	4	4	0.1	N0	3	67	0.011915	low_risk	NO
	tcga-ej-a65e	4	3	0.1	N0	3	67	0.005735	low_risk	NO
	tcga-ej-a65g	3	5	0.1	N0	2	57	0.006037	low_risk	NO
	tcga-ej-a65m	3	3	0.1	N0	2	65	0.00152	low_risk	NO
	tcga-ej-a7nf	3	4	0.1	N0	2	56	0.002864	low_risk	NO
	tcga-ej-a7ng	3	4	0.1	N0	3	64	0.003545	low_risk	NO
	tcga-ej-a7nh	3	4	0.1	N0	2	57	0.002897	low_risk	NO
	tcga-e	4	4	0.	N0	2	6	0.00975	low_risk	NO

	j-a7nj			1			0			
	tcga-ej-a7nk	3	4	0.1	N0	3	59	0.003349	low_risk	NO
	tcga-ej-a8fn	3	4	0.1	N0	2	59	0.002963	low_risk	NO
	tcga-ej-a8fo	4	3	0.1	N0	3	52	0.004837	low_risk	NO
	tcga-ej-a8fp	3	5	0.2	N0	2	60	0.006259	low_risk	NO
	tcga-ej-a8fs	3	4	0.3	N0	3	67	0.003685	low_risk	NO
	tcga-ej-ab20	3	3	0.1	N0	2	62	0.001469	low_risk	NO
	tcga-ej-ab27	3	3	0.1	N0	2	51	0.001296	low_risk	NO
	tcga-fc-a66v	4	3	10.8	N0	3	75	0.007929	low_risk	NO
	tcga-fc-a8o0	3	3	7.82	N0	3	69	0.00213	low_risk	NO
	tcga-g9-6332	4	3	0.73	N0	3	55	0.005074	low_risk	NO
	tcga-g9-6333	4	3	0.03	N0	2	66	0.00501	low_risk	NO
	tcga-g9-6338	4	3	0.05	N0	3	61	0.005351	low_risk	NO
	tcga-g9-6339	3	4	0.03	N0	3	58	0.003306	low_risk	NO
	tcga-g9-6348	3	4	0.02	N0	2	68	0.003277	low_risk	NO
	tcga-g9-6361	3	4	0.1	N0	3	62	0.003466	low_risk	NO
	tcga-g9-6362	4	3	0.07	N0	3	58	0.005175	low_risk	NO
	tcga-g9-6363	4	3	0.03	N0	4	64	0.006253	low_risk	NO
	tcga-g	3	4	0.	N0	4	7	0.00438	low_risk	NO

	9-636 5			01			2			
	tcga-g 9-636 9	4	3	0. 03	N0	2	5 5	0.004422	low_risk	NO
	tcga-g 9-637 0	3	4	0. 03	N0	3	5 2	0.003088	low_risk	NO
	tcga-g 9-637 7	3	4	0. 03	N0	2	6 1	0.003027	low_risk	NO
	tcga-g 9-637 9	4	3	0. 03	N0	3	6 9	0.005858	low_risk	NO
	tcga-g 9-638 4	3	4	0. 03	N0	3	5 4	0.003159	low_risk	NO
	tcga-g 9-649 4	4	3	1. 13	N0	4	6 7	0.006627	low_risk	NO
	tcga-g 9-649 6	4	3	0. 03	N0	2	6 2	0.004788	low_risk	NO
	tcga-g 9-751 0	4	4	0. 03	N0	3	6 6	0.011764	low_risk	NO
	tcga-g 9-752 2	3	4	0. 1	N0	2	4 9	0.002645	low_risk	NO
	tcga-g 9-752 5	4	3	0. 03	N0	2	6 4	0.004898	low_risk	NO
	tcga-g 9-a9s7	4	4	0. 13	N0	3	5 5	0.010413	low_risk	NO
	tcga-h c-707 8	3	4	0. 01	N0	3	7 0	0.003788	low_risk	NO
	tcga-h c-707 9	3	4	0. 1	N0	3	5 1	0.003058	low_risk	NO
	tcga-h c-708 0	3	4	0. 1	N0	2	6 6	0.003209	low_risk	NO
	tcga-h	3	3	0.	N0	2	6	0.001451	low_risk	NO

	c-7209			05			1			
	tcga-hc-7210	3	4	0.1	N0	2	75	0.003555	low_risk	NO
	tcga-hc-7211	3	4	10.9	N0	2	62	0.003883	low_risk	NO
	tcga-hc-7212	3	4	0.28	N0	2	56	0.002875	low_risk	NO
	tcga-hc-7230	3	4	3	N0	2	51	0.002883	low_risk	NO
	tcga-hc-7736	3	4	0.02	N0	2	59	0.002958	low_risk	NO
	tcga-hc-7737	3	4	0.1	N1	2	55	0.011488	low_risk	NO
	tcga-hc-7740	3	4	0	N0	2	60	0.002991	low_risk	NO
	tcga-hc-7742	3	4	0.1	N0	3	58	0.003312	low_risk	NO
	tcga-hc-7744	4	3	0.04	N0	3	47	0.004564	low_risk	NO
	tcga-hc-7747	3	4	0.19	N0	2	53	0.002773	low_risk	NO
	tcga-hc-7749	4	3	0.1	N0	3	70	0.005933	low_risk	NO
	tcga-hc-7752	3	4	0.1	N0	2	61	0.003032	low_risk	NO
	tcga-hc-7818	3	4	0.01	N0	2	67	0.003239	low_risk	NO
	tcga-hc-7819	4	4	0.01	N0	2	54	0.009092	low_risk	NO

	tcga-hc-8216	4	3	0.19	N0	3	59	0.005247	low_risk	NO
	tcga-hc-8257	3	4	0.1	N1	3	70	0.015355	low_risk	NO
	tcga-hc-8258	3	3	0.01	N0	2	57	0.001385	low_risk	NO
	tcga-hc-8260	3	4	0.01	N0	2	44	0.002493	low_risk	NO
	tcga-hc-8262	4	4	0.1	N0	2	57	0.009425	low_risk	NO
	tcga-hc-8265	3	5	0.1	N0	3	67	0.007639	low_risk	NO
	tcga-hc-a76x	3	4	0.01	N0	2	62	0.00306	low_risk	NO
	tcga-hc-a8d0	3	4	0.01	N0	2	62	0.00306	low_risk	NO
	tcga-hc-a8d1	3	4	0.01	N0	2	69	0.003314	low_risk	NO
	tcga-hi-7169	3	4	0.1	N0	3	55	0.0032	low_risk	NO
	tcga-hi-7170	3	3	0.1	N0	2	58	0.001403	low_risk	NO
	tcga-j4-8200	3	4	0.1	N0	2	47	0.002585	low_risk	NO
	tcga-j4-a67l	3	4	0.01	N0	3	55	0.003194	low_risk	NO
	tcga-j4-a67m	4	3	0.1	N0	3	55	0.005005	low_risk	NO
	tcga-j4-a67n	3	4	0.7	N0	3	61	0.003472	low_risk	NO
	tcga-j4-a67o	3	4	0.1	N0	2	59	0.002963	low_risk	NO

	tcga-j4-a67r	4	3	0.1	N0	2	65	0.004961	low_risk	NO
	tcga-j4-a67s	3	4	0.1	N0	3	64	0.003545	low_risk	NO
	tcga-j4-a6g1	4	4	0.1	N0	3	68	0.01205	low_risk	NO
	tcga-j4-a6g3	4	4	0.4	N0	3	57	0.010713	low_risk	NO
	tcga-j4-a83i	4	3	0.1	N0	3	64	0.005543	low_risk	NO
	tcga-j4-a83j	3	4	0.04	N0	2	68	0.003279	low_risk	NO
	tcga-j4-a83l	3	4	0.1	N0	2	61	0.003032	low_risk	NO
	tcga-j4-a83m	4	3	0.2	N0	2	65	0.004972	low_risk	NO
	tcga-j4-aatv	3	3	0	N0	2	71	0.001624	low_risk	NO
	tcga-j4-aau2	3	3	0	N0	2	59	0.001416	low_risk	NO
	tcga-kc-a4bl	3	4	3.15	N0	2	66	0.00343	low_risk	NO
	tcga-kc-a4bn	3	4	0	N0	2	56	0.002858	low_risk	NO
	tcga-kc-a4bo	3	4	0.04	N0	2	56	0.00286	low_risk	NO
	tcga-kc-a7f3	3	4	0	N0	2	68	0.003276	low_risk	NO
	tcga-kc-a7f5	4	3	0	N0	2	55	0.004419	low_risk	NO
	tcga-kc-a7f6	3	4	0	N0	2	64	0.00313	low_risk	NO
	tcga-kc-a7fd	3	4	0	N0	3	62	0.003458	low_risk	NO
	tcga-kc-a7fe	3	4	0	N0	2	62	0.003059	low_risk	NO
	tcga-kk-a59z	4	3	0.1	N0	2	67	0.005075	low_risk	NO

	tcga-kk-a6dy	3	4	0.1	N0	2	50	0.002675	low_risk	NO
	tcga-kk-a6e2	3	4	0.1	N0	2	55	0.002831	low_risk	NO
	tcga-kk-a6e3	3	4	0.1	N0	2	56	0.002864	low_risk	NO
	tcga-kk-a6e4	3	4	0.1	N0	2	69	0.00332	low_risk	NO
	tcga-kk-a6e5	4	3	0.1	N0	3	63	0.00548	low_risk	NO
	tcga-kk-a7aq	4	3	0.1	N0	2	62	0.004795	low_risk	NO
	tcga-kk-a7av	3	4	0.1	N0	2	58	0.00293	low_risk	NO
	tcga-kk-a7ay	4	3	0.2	N0	2	61	0.004751	low_risk	NO
	tcga-kk-a7b1	4	3	0.1	N0	3	65	0.005606	low_risk	NO
	tcga-kk-a8i5	3	4	0.1	N0	2	56	0.002864	low_risk	NO
	tcga-kk-a8i6	4	3	0.2	N0	3	59	0.005249	low_risk	NO
	tcga-kk-a8i9	4	4	0.1	N0	3	61	0.011135	low_risk	NO
	tcga-kk-a8if	4	3	0.7	N0	3	57	0.005187	low_risk	NO
	tcga-kk-a8ig	4	3	0.1	N0	3	56	0.005062	low_risk	NO
	tcga-kk-a8ih	4	3	0.1	N0	3	51	0.004782	low_risk	NO
	tcga-kk-a8im	4	3	0.1	N0	2	56	0.004479	low_risk	NO
	tcga-qu-a6il	3	4	0.2	N0	2	65	0.00318	low_risk	NO
	tcga-qu-a6in	3	4	0.1	N0	2	61	0.003032	low_risk	NO

	tcga-qu-a6io	3	3	0.1	N0	2	54	0.001341	low_risk	NO
	tcga-qu-a6ip	3	3	0	N0	2	66	0.001534	low_risk	NO
	tcga-su-a7e7	5	3	0.01	N0	2	61	0.015331	low_risk	NO
	tcga-tp-a8tt	4	3	0.024	N0	3	64	0.005534	low_risk	NO
	tcga-tp-a8tv	4	3	0.019	N0	2	63	0.004841	low_risk	NO
	tcga-v1-a8mf	3	3	0.14	N0	3	66	0.00174	low_risk	NO
	tcga-v1-a8mj	3	4	0.015	N1	3	59	0.013543	low_risk	NO
	tcga-v1-a8mk	3	3	0.83	N0	2	42	0.001188	low_risk	NO
	tcga-v1-a8ml	3	4	0.01	N0	2	64	0.003131	low_risk	NO
	tcga-v1-a8mm	4	3	0.37	N0	3	61	0.005389	low_risk	NO
	tcga-v1-a8mu	3	4	0.015	N1	3	57	0.013241	low_risk	NO
	tcga-v1-a8wl	3	4	0.015	N0	3	65	0.003579	low_risk	NO
	tcga-v1-a8x3	3	4	0.03	N0	2	52	0.002732	low_risk	NO
	tcga-v1-a9o9	3	5	5.04	N0	3	57	0.007596	low_risk	NO
	tcga-v1-a9ox	3	5	0.04	N0	2	57	0.006029	low_risk	NO
	tcga-v	4	4	0.	N0	3	6	0.012163	low_risk	NO

	1-a9zk			01			9			
	tcga-v1-a9zr	4	4	1.44	N0	4	69	0.014163	low_risk	NO
	tcga-vn-a88i	4	4	0.16	N0	3	59	0.010901	low_risk	NO
	tcga-vn-a88l	3	4	0	N0	2	55	0.002825	low_risk	NO
	tcga-vn-a88n	4	3	0	N0	2	62	0.004785	low_risk	NO
	tcga-vn-a88o	3	4	0	N0	2	49	0.002639	low_risk	NO
	tcga-vn-a88p	3	4	0	N0	2	60	0.002991	low_risk	NO
	tcga-vn-a88q	4	4	0	N0	2	61	0.009839	low_risk	NO
	tcga-vn-a88r	4	4	0.15	N0	2	53	0.009018	low_risk	NO
	tcga-vn-a943	4	4	0.01	N0	2	72	0.011143	low_risk	NO
	tcga-vp-a875	3	4	0.01	N0	3	68	0.003703	low_risk	NO
	tcga-vp-a876	3	5	0.1	N0	2	46	0.005328	low_risk	NO
	tcga-vp-a87b	4	4	3.37	N0	2	64	0.010951	low_risk	NO
	tcga-vp-a87c	4	3	0.5	N0	2	67	0.00512	low_risk	NO
	tcga-vp-a87e	3	3	0.11	N0	2	60	0.001436	low_risk	NO
	tcga-vp-a87j	3	4	0.13	N1	3	57	0.013274	low_risk	NO
	tcga-vp-a87k	4	4	0.3	N0	3	63	0.011439	low_risk	NO

	tcga-ww-a8zi	4	4	0	N0	2	70	0.010892	low_risk	NO
	tcga-xa-a8jr	4	3	0	N0	2	69	0.00518	low_risk	NO
	tcga-xk-aair	4	4	0.09	N0	2	76	0.011678	low_risk	NO
	tcga-xk-aaja	4	3	0.09	N0	3	62	0.005417	low_risk	NO
	tcga-xk-aajp	4	3	0.09	N0	3	67	0.005734	low_risk	NO
	tcga-xk-aajr	4	3	0.09	N0	3	62	0.005417	low_risk	NO
	tcga-xk-aajt	4	3	0.09	N0	3	75	0.006278	low_risk	NO
	tcga-xk-aaju	4	3	0.09	N0	2	66	0.005017	low_risk	NO
	tcga-y6-a8tl	3	3	0	N0	3	65	0.001715	low_risk	NO
	tcga-yl-a8ho	4	3	0.01	N0	3	67	0.005724	low_risk	NO
	tcga-yl-a8sa	4	4	0.06	N0	3	69	0.012176	low_risk	NO
	tcga-yl-a8sf	4	4	0.01	N0	3	63	0.011367	low_risk	NO
	tcga-yl-a8sh	4	3	0.06	N0	3	70	0.005928	low_risk	NO
