# Supplementary material for: An Integrative Multi-Omics Analysis Based on Nomogram for Predicting Prostate Cancer Bone Metastasis Incidence
Source: Genet Res (Camb). 2022 Sep 29;2022:8213723. doi: 10.1155/2022/8213723 (PMC9537037; doi:10.1155/2022/8213723)
Supplement: Supplementary Materials — Figure S1: associated with Figure 2. Figure S2: associated with Figure 5. Figure S3: associated with Figure 6. Data 1-1: SEER data baseline characteristics of patient with prostate cancer; Data 1-2: train group ROC and validation ROC, Delong's test; Data 1-3: clinical features of TCGA samples and predictive results. Data 2: TMB scores of TCGA samples. Data 3: genes with different frequencies of CNVs between LRBM and HRBM cohorts. Data 4-1: DElncRNAs; Data 4-2: DEmiRNAs; Data 4-3: DEmRNAs. Data 5: gene module. Data 6-1: 29 immune microenvironment signatures; Data-2: ssGSEA score of immune microenvironment signature; Data 6-3: stromal score, immune score, estimate score, and tumor purity of TCGA samples. [file 8213723.f1.zip › 8213723.f1/supplementary data2 (1).pdf]

Supplementary Data2: TMB scores of TCGA samples		
	**id**	**TMB**
	TCGA-XJ-A83H	0.5
	TCGA-EJ-7789	0.789474
	TCGA-V1-A8WV	1.157895
	TCGA-VP-A87K	0.736842
	TCGA-HC-7077	1.421053
	TCGA-CH-5792	0.842105
	TCGA-CH-5761	1.421053
	TCGA-V1-A9ZG	0.842105
	TCGA-HC-8213	0.342105
	TCGA-HC-A48F	0.789474
	TCGA-V1-A9ZK	0.526316
	TCGA-KK-A8IB	1
	TCGA-V1-A9OY	0.5
	TCGA-J4-A67T	0.447368
	TCGA-2A-A8VL	0.842105
	TCGA-XK-AAJU	0.052632
	TCGA-V1-A8X3	0.605263
	TCGA-EJ-5524	0.736842
	TCGA-YL-A8HM	1.105263
	TCGA-HC-A6HX	0.394737
	TCGA-EJ-7321	0.526316
	TCGA-HC-8258	0.131579
	TCGA-EJ-A8FN	0.921053
	TCGA-J4-A67R	0.131579
	TCGA-V1-A9OL	0.868421
	TCGA-HC-A6AP	0.421053
	TCGA-J4-A67S	0.394737
	TCGA-KK-A6E0	0.842105
	TCGA-EJ-A65G	0.473684
	TCGA-EJ-5512	0.421053
	TCGA-EJ-5532	0.736842
	TCGA-CH-5746	0.236842
	TCGA-V1-A9O9	0.657895
	TCGA-YL-A9WY	0.894737
	TCGA-KC-A7F6	0.526316
	TCGA-YL-A8S9	0.921053
	TCGA-KK-A8I5	1.078947
	TCGA-CH-5767	0.815789
	TCGA-HC-A76W	0.894737
	TCGA-M7-A723	0.026316

	TCGA-XQ-A8TA	3
	TCGA-EJ-5504	1.052632
	TCGA-KK-A7B1	0.447368
	TCGA-HC-8262	0.868421
	TCGA-CH-5794	0.815789
	TCGA-EJ-7781	0.842105
	TCGA-KK-A8I7	0.210526
	TCGA-VN-A88R	0.657895
	TCGA-J4-A6M7	0.447368
	TCGA-EJ-8474	0.947368
	TCGA-EJ-7786	0.368421
	TCGA-FC-A5OB	0.578947
	TCGA-EJ-5517	0.657895
	TCGA-G9-A9S7	0.578947
	TCGA-2A-A8VT	0.868421
	TCGA-VP-A879	0.868421
	TCGA-KK-A6E7	0.736842
	TCGA-KK-A7AW	0.789474
	TCGA-HC-A6AL	1.394737
	TCGA-YL-A8SP	0.710526
	TCGA-EJ-5511	0.973684
	TCGA-G9-6373	0.763158
	TCGA-M7-A71Z	0.684211
	TCGA-CH-5772	0.736842
	TCGA-CH-5789	0.026316
	TCGA-HC-7817	0.421053
	TCGA-ZG-A9L9	1.026316
	TCGA-EJ-7325	0.815789
	TCGA-EJ-7317	0.789474
	TCGA-CH-5766	0.710526
	TCGA-KK-A8IL	0.842105
	TCGA-HC-7819	0.342105
	TCGA-HC-A8D0	0.605263
	TCGA-VN-A88P	1.210526
	TCGA-J9-A8CP	0.789474
	TCGA-HC-A6AQ	0.342105
	TCGA-KC-A4BR	0.026316
	TCGA-VP-A87C	0.578947
	TCGA-HC-A8CY	0.868421
	TCGA-EJ-A65D	0.973684
	TCGA-X4-A8KS	0.263158
	TCGA-YL-A8SI	0.210526
	TCGA-G9-6351	0.473684

	TCGA-VP-A87H	0.736842
	TCGA-Y6-A9XI	1.184211
	TCGA-QU-A6IN	0.684211
	TCGA-EJ-A65M	0.789474
	TCGA-2A-A8VO	0.815789
	TCGA-J9-A8CM	2.078947
	TCGA-ZG-A9LY	0.894737
	TCGA-TP-A8TV	1.052632
	TCGA-YL-A8SC	1
	TCGA-V1-A8ML	0.236842
	TCGA-XK-AAIW	236
	TCGA-QU-A6IP	0.473684
	TCGA-YL-A8SO	0.842105
	TCGA-YL-A8SF	0.552632
	TCGA-KK-A8I6	0.657895
	TCGA-KK-A6E2	1
	TCGA-YL-A8SL	1.263158
	TCGA-G9-6364	0.473684
	TCGA-2A-AAYO	0.526316
	TCGA-V1-A9OA	0.5
	TCGA-G9-7523	0.236842
	TCGA-HC-A6AS	0.394737
	TCGA-EJ-5516	0.447368
	TCGA-EJ-7793	0.131579
	TCGA-V1-A9O7	0.473684
	TCGA-HC-A6AO	0.947368
	TCGA-KK-A8IK	0.894737
	TCGA-KK-A8ID	1.710526
	TCGA-XJ-A9DI	0.105263
	TCGA-G9-6362	0.894737
	TCGA-YL-A8HK	0.5
	TCGA-XK-AAJP	0.947368
	TCGA-FC-A8O0	0.342105
	TCGA-ZG-A9LZ	0.736842
	TCGA-G9-6377	0.5
	TCGA-TP-A8TT	0.763158
	TCGA-EJ-A6RA	0.052632
	TCGA-HC-7209	0.657895
	TCGA-G9-7525	0.789474
	TCGA-EJ-8469	0.605263
	TCGA-J9-A52C	16.21053
	TCGA-YJ-A8SW	1.684211
	TCGA-EJ-A65J	0.921053

	TCGA-HC-8216	1.315789
	TCGA-KK-A6E1	0.684211
	TCGA-EJ-5497	0.342105
	TCGA-G9-6384	0.526316
	TCGA-KK-A7AQ	0.657895
	TCGA-V1-A9ZI	0.921053
	TCGA-EJ-5525	1.815789
	TCGA-EJ-8470	0.657895
	TCGA-EJ-A8FS	1
	TCGA-EJ-5519	2
	TCGA-YL-A8SJ	0.710526
	TCGA-HC-7738	0.789474
	TCGA-ZG-A9LN	0.789474
	TCGA-EJ-7218	0.421053
	TCGA-KC-A7F5	0.947368
	TCGA-H9-A6BX	0.052632
	TCGA-V1-A9Z9	0.868421
	TCGA-XK-AAJR	0.789474
	TCGA-EJ-5530	0.578947
	TCGA-EJ-A8FO	0.289474
	TCGA-EJ-5505	0.947368
	TCGA-KK-A5A1	2.368421
	TCGA-G9-6366	0.657895
	TCGA-VP-A876	0.526316
	TCGA-G9-6361	0.868421
	TCGA-M7-A725	0.815789
	TCGA-HC-7078	0.868421
	TCGA-EJ-5521	0.868421
	TCGA-HC-7750	0.315789
	TCGA-EJ-5496	0.815789
	TCGA-EJ-A46B	0.052632
	TCGA-J4-A83N	0.394737
	TCGA-YL-A9WI	1.210526
	TCGA-CH-5744	0.789474
	TCGA-EJ-A8FP	0.026316
	TCGA-HC-A9TH	1.315789
	TCGA-KC-A7FD	1.157895
	TCGA-CH-5739	0.921053
	TCGA-CH-5754	2.578947
	TCGA-HI-7171	0.789474
	TCGA-KK-A8IA	0.973684
	TCGA-V1-A9ZR	1.105263
	TCGA-G9-6333	0.578947

	TCGA-HC-8261	0.078947
	TCGA-HI-7168	1.052632
	TCGA-HC-7821	0.605263
	TCGA-HC-A76X	0.657895
	TCGA-KK-A6E8	0.947368
	TCGA-SU-A7E7	0.368421
	TCGA-EJ-A46I	0.052632
	TCGA-CH-5748	0.394737
	TCGA-ZG-A9KY	1.868421
	TCGA-KK-A8IJ	0.605263
	TCGA-KK-A8IC	0.447368
	TCGA-EJ-A65F	1.842105
	TCGA-EJ-A46D	0.131579
	TCGA-J4-A67M	0.842105
	TCGA-XJ-A9DX	1.815789
	TCGA-CH-5768	1
	TCGA-KK-A7B4	0.921053
	TCGA-HC-8260	0.157895
	TCGA-EJ-AB20	0.236842
	TCGA-HC-7230	0.578947
	TCGA-G9-6339	1.263158
	TCGA-EJ-7784	0.710526
	TCGA-EJ-8472	1.447368
	TCGA-G9-6371	0.447368
	TCGA-ZG-A9LU	0.289474
	TCGA-EJ-5515	0.078947
	TCGA-HC-7737	0.236842
	TCGA-J4-A67N	0.552632
	TCGA-KK-A6E5	0.342105
	TCGA-EJ-A7NN	1
	TCGA-VP-A875	0.973684
	TCGA-G9-6499	0.894737
	TCGA-VN-A88N	0.447368
	TCGA-G9-7522	0.210526
	TCGA-YL-A8SQ	0.894737
	TCGA-YL-A9WL	0.605263
	TCGA-V1-A9O5	0.868421
	TCGA-CH-5751	0.736842
	TCGA-G9-6332	0.710526
	TCGA-CH-5738	0.263158
	TCGA-HC-A6AN	1
	TCGA-VP-A87B	0.842105
	TCGA-KK-A8II	0.947368

	TCGA-HC-A9TE	1.289474
	TCGA-VP-A872	0.578947
	TCGA-EJ-5501	0.526316
	TCGA-ZG-A9M4	1
	TCGA-CH-5752	0.894737
	TCGA-KK-A59V	25.05263
	TCGA-EJ-5510	0.657895
	TCGA-HC-8259	0.421053
	TCGA-EJ-AB27	0.210526
	TCGA-G9-A9S4	1.052632
	TCGA-KC-A4BN	0.315789
	TCGA-ZG-A9L1	0.842105
	TCGA-CH-5765	0.973684
	TCGA-HC-8256	0.710526
	TCGA-G9-6354	0.5
	TCGA-J9-A8CN	0.473684
	TCGA-CH-5745	0.657895
	TCGA-G9-6378	0.657895
	TCGA-G9-6496	1.078947
	TCGA-G9-6365	0.473684
	TCGA-KK-A7AP	0.868421
	TCGA-V1-A8WN	0.394737
	TCGA-M7-A722	0.921053
	TCGA-EJ-5509	0.394737
	TCGA-G9-6370	0.026316
	TCGA-V1-A8MF	0.868421
	TCGA-XQ-A8TB	0.763158
	TCGA-EJ-A46G	1.526316
	TCGA-2A-A8W3	1.131579
	TCGA-J4-A6G3	0.236842
	TCGA-M7-A71Y	0.184211
	TCGA-KK-A7B0	0.736842
	TCGA-J4-A67Q	0.394737
	TCGA-4L-AA1F	0.552632
	TCGA-XK-AAJ3	1.736842
	TCGA-YL-A8HJ	0.763158
	TCGA-M7-A721	0.473684
	TCGA-XK-AAIR	0.473684
	TCGA-FC-7961	1.184211
	TCGA-2A-A8VV	0.342105
	TCGA-J9-A52E	0.815789
	TCGA-G9-6494	0.815789
	TCGA-J4-AAU2	0.526316

	TCGA-KC-A7FE	0.052632
	TCGA-YL-A8HL	0.657895
	TCGA-V1-A9OX	0.526316
	TCGA-EJ-7794	0.289474
	TCGA-HC-7210	1.105263
	TCGA-HC-7820	0.657895
	TCGA-V1-A8MM	0.578947
	TCGA-XK-AAJA	1.105263
	TCGA-HC-A8D1	0.578947
	TCGA-G9-A9S0	0.684211
	TCGA-G9-6379	0.605263
	TCGA-YL-A9WJ	0.552632
	TCGA-VN-A88L	0.289474
	TCGA-FC-A4JI	0.973684
	TCGA-KC-A7FA	0.894737
	TCGA-J4-A67O	0.394737
	TCGA-EJ-5498	1
	TCGA-G9-6347	0.236842
	TCGA-J9-A52D	1.131579
	TCGA-G9-6353	0.026316
	TCGA-EJ-A6RC	0.131579
	TCGA-V1-A9OQ	0.947368
	TCGA-EJ-7791	0.157895
	TCGA-XJ-A83F	0.815789
	TCGA-KK-A7AU	0.605263
	TCGA-ZG-A8QX	0.736842
	TCGA-EJ-5507	0.842105
	TCGA-HI-7169	0.605263
	TCGA-ZG-A8QZ	0.289474
	TCGA-VP-A87E	0.289474
	TCGA-KK-A7AZ	0.763158
	TCGA-J9-A8CL	1.421053
	TCGA-HC-8266	0.5
	TCGA-HC-7747	0.394737
	TCGA-CH-5750	1.052632
	TCGA-KK-A8IM	0.710526
	TCGA-EJ-7328	0.789474
	TCGA-YL-A8SK	0.921053
	TCGA-EJ-7125	0.710526
	TCGA-EJ-5522	0.657895
	TCGA-EJ-7314	0.736842
	TCGA-HC-7231	0.815789
	TCGA-KK-A8IF	0.710526

	TCGA-EJ-5499	0.947368
	TCGA-V1-A9Z7	0.763158
	TCGA-KK-A6E6	0.973684
	TCGA-CH-5762	1.263158
	TCGA-YL-A8SA	0.868421
	TCGA-EJ-A46F	1
	TCGA-KK-A59Y	0.631579
	TCGA-HC-7736	0.552632
	TCGA-J4-A83K	0.552632
	TCGA-G9-6498	0.657895
	TCGA-VN-A88K	0.736842
	TCGA-G9-7510	0.526316
	TCGA-CH-5771	0.5
	TCGA-EJ-7782	10.89474
	TCGA-EJ-5494	0.342105
	TCGA-CH-5737	1.315789
	TCGA-2A-A8VX	0.842105
	TCGA-YL-A8SB	0.605263
	TCGA-XJ-A9DQ	0.078947
	TCGA-EJ-A7NF	0.526316
	TCGA-CH-5788	0.947368
	TCGA-ZG-A9LM	0.894737
	TCGA-XK-AAJT	0.684211
	TCGA-G9-6348	0.973684
	TCGA-EJ-5527	0.578947
	TCGA-VN-A88I	0.131579
	TCGA-HC-7742	0.921053
	TCGA-XK-AAK1	1.210526
	TCGA-VN-A943	0.578947
	TCGA-EJ-7331	0.631579
	TCGA-EJ-7788	0.947368
	TCGA-EJ-A65B	0.394737
	TCGA-EJ-A7NK	0.078947
	TCGA-EJ-5502	0.026316
	TCGA-EJ-7783	0.736842
	TCGA-G9-6343	0.394737
	TCGA-KK-A7AY	1.210526
	TCGA-KK-A8IG	0.657895
	TCGA-XA-A8JR	0.421053
	TCGA-EJ-5506	0.052632
	TCGA-J4-8198	0.815789
	TCGA-HC-7212	0.421053
	TCGA-J4-A67K	0.684211

	TCGA-2A-AAYF	0.657895
	TCGA-HC-8265	1.210526
	TCGA-QU-A6IO	0.842105
	TCGA-J4-8200	0.368421
	TCGA-MG-AAMC	0.631579
	TCGA-HC-8257	1.157895
	TCGA-VN-A88M	0.315789
	TCGA-EJ-7318	0.578947
	TCGA-HC-7818	0.131579
	TCGA-J4-A83L	0.342105
	TCGA-HC-A631	1.131579
	TCGA-VP-A87D	1.210526
	TCGA-CH-5763	0.026316
	TCGA-KK-A6E3	0.289474
	TCGA-EJ-7792	0.421053
	TCGA-V1-A9OH	1.157895
	TCGA-ZG-A9L5	1.184211
	TCGA-J9-A8CK	0.605263
	TCGA-YL-A9WH	1.578947
	TCGA-CH-5791	0.973684
	TCGA-ZG-A8QW	0.763158
	TCGA-X4-A8KQ	1.236842
	TCGA-Y6-A8TL	0.657895
	TCGA-KK-A7B3	1.078947
	TCGA-CH-5743	0.026316
	TCGA-EJ-5526	0.894737
	TCGA-CH-5740	0.447368
	TCGA-ZG-A9L2	0.657895
	TCGA-CH-5764	0.605263
	TCGA-HC-A632	0.973684
	TCGA-V1-A8MG	0.421053
	TCGA-KK-A6E4	1.052632
	TCGA-V1-A8WW	0.894737
	TCGA-EJ-7312	0.710526
	TCGA-KK-A8I8	0.657895
	TCGA-QU-A6IL	0.684211
	TCGA-KK-A8IH	0.657895
	TCGA-2A-AAYU	0.736842
	TCGA-ZG-A9MC	0.315789
	TCGA-HC-A4ZV	3.447368
	TCGA-G9-6338	0.736842
	TCGA-HC-7075	0.684211
	TCGA-EJ-7327	0.631579

	TCGA-HC-7081	0.578947
	TCGA-V1-A9OT	0.973684
	TCGA-VN-A88O	0.657895
	TCGA-YL-A8HO	0.631579
	TCGA-EJ-7330	1.078947
	TCGA-G9-7519	0.552632
	TCGA-G9-6369	0.684211
	TCGA-KK-A59Z	1.394737
	TCGA-G9-6356	0.236842
	TCGA-HI-7170	0.710526
	TCGA-G9-6385	0.078947
	TCGA-HC-7752	0.684211
	TCGA-EJ-7123	1
	TCGA-ZG-A9L4	0.368421
	TCGA-EJ-8468	0.631579
	TCGA-ZG-A8QY	1.736842
	TCGA-EJ-7785	0.5
	TCGA-ZG-A9LB	1.342105
	TCGA-EJ-A65E	1.052632
	TCGA-G9-7521	0.815789
	TCGA-VP-AA1N	0.921053
	TCGA-J4-AATZ	0.842105
	TCGA-HC-8264	0.973684
	TCGA-ZG-A9N3	1.473684
	TCGA-WW-A8ZI	0.842105
	TCGA-HC-7213	0.815789
	TCGA-KK-A6DY	0.657895
	TCGA-M7-A724	0.842105
	TCGA-HC-7749	1.105263
	TCGA-2A-A8W1	0.526316
	TCGA-HC-7233	0.789474
	TCGA-J9-A52B	0.921053
	TCGA-HC-7748	0.578947
	TCGA-CH-5741	0.526316
	TCGA-ZG-A9LS	0.789474
	TCGA-G9-6342	0.552632
	TCGA-EJ-A7NM	1.552632
	TCGA-KC-A4BV	0.894737
	TCGA-KC-A7F3	0.736842
	TCGA-EJ-A7NG	0.026316
	TCGA-HC-A6HY	0.657895
	TCGA-ZG-A9NI	2.210526
	TCGA-H9-A6BY	0.710526

	TCGA-ZG-A9L0	1.131579
	TCGA-EJ-5495	2
	TCGA-FC-A6HD	0.842105
	TCGA-KK-A59X	1.473684
	TCGA-EJ-A7NH	0.421053
	TCGA-J4-A67L	0.684211
	TCGA-HC-7745	0.631579
	TCGA-HC-7740	0.342105
	TCGA-YL-A9WK	0.552632
	TCGA-V1-A8MJ	0.342105
	TCGA-EJ-5508	0.473684
	TCGA-QU-A6IM	1.763158
	TCGA-EJ-5542	0.526316
	TCGA-ZG-A9L6	0.605263
	TCGA-YL-A9WX	0.184211
	TCGA-VP-A87J	0.763158
	TCGA-KK-A8I9	0.815789
	TCGA-KK-A8I4	0.5
	TCGA-G9-6363	0.868421
	TCGA-YL-A8SH	0.763158
	TCGA-G9-6329	0.578947
	TCGA-EJ-7315	0.631579
	TCGA-V1-A8WL	0.868421
	TCGA-HC-7080	0.921053
	TCGA-HC-7232	0.763158
	TCGA-EJ-7797	0.421053
	TCGA-EJ-5518	1.052632
	TCGA-VP-A878	0.894737
	TCGA-H9-7775	0.815789
	TCGA-J4-A83M	0.657895
	TCGA-KC-A4BL	0.052632
	TCGA-EJ-7115	1.210526
	TCGA-EJ-5531	0.578947
	TCGA-FC-7708	0.526316
	TCGA-ZG-A9ND	0.815789
	TCGA-G9-7509	0.342105
	TCGA-HC-7744	0.657895
	TCGA-CH-5769	1.342105
	TCGA-HC-7211	0.421053
	TCGA-KK-A7B2	0.052632
	TCGA-XK-AAIV	1.026316
	TCGA-VN-A88Q	1.026316
	TCGA-J4-A83I	0.736842

	TCGA-TK-A8OK	1.157895
	TCGA-J4-A83J	1.236842
	TCGA-V1-A8MU	0.842105
	TCGA-V1-A8WS	0.578947
	TCGA-EJ-5514	0.973684
	TCGA-XJ-A83G	0.763158
	TCGA-V1-A9OF	0.342105
	TCGA-YL-A8S8	1.131579
	TCGA-CH-5753	1.078947
	TCGA-YL-A8SR	0.578947
	TCGA-CH-5790	0.289474
	TCGA-KK-A7AV	0.368421
	TCGA-V1-A9Z8	0.526316
	TCGA-G9-6336	0.447368
	TCGA-G9-6336	0.447368
