# Supplementary material for: An Integrative Multi-Omics Analysis Based on Nomogram for Predicting Prostate Cancer Bone Metastasis Incidence
Source: Genet Res (Camb). 2022 Sep 29;2022:8213723. doi: 10.1155/2022/8213723 (PMC9537037; doi:10.1155/2022/8213723)
Supplement: Supplementary Materials — Figure S1: associated with Figure 2. Figure S2: associated with Figure 5. Figure S3: associated with Figure 6. Data 1-1: SEER data baseline characteristics of patient with prostate cancer; Data 1-2: train group ROC and validation ROC, Delong's test; Data 1-3: clinical features of TCGA samples and predictive results. Data 2: TMB scores of TCGA samples. Data 3: genes with different frequencies of CNVs between LRBM and HRBM cohorts. Data 4-1: DElncRNAs; Data 4-2: DEmiRNAs; Data 4-3: DEmRNAs. Data 5: gene module. Data 6-1: 29 immune microenvironment signatures; Data-2: ssGSEA score of immune microenvironment signature; Data 6-3: stromal score, immune score, estimate score, and tumor purity of TCGA samples. [file 8213723.f1.zip › 8213723.f1/supplementary data3 (1).pdf]

Supplementary Data3:genes with different frequency of CNVs between LRBM and HRBM cohort

Gene	CNV ratio of LRBM cohort	CNV ratio of HRBM cohort	Stat	Pvalue	adjPvalue
CSMD3	0.118644	0.409091	39.57687	3.15E-10	6.00E-06
TRMT12	0.088983	0.393939	47.60756	5.21E-12	9.91E-08
TAT	0.097458	0.295455	22.29626	2.34E-06	0.044456
TTPA	0.080508	0.30303	29.5054	5.58E-08	0.001061
ADAMTS18	0.127119	0.356061	25.45059	4.54E-07	0.008635
NAE1	0.059322	0.234848	22.69198	1.90E-06	0.03618
OPRK1	0.067797	0.310606	36.29438	1.70E-09	3.23E-05
MAF1	0.088983	0.356061	38.3313	5.97E-10	1.14E-05
MT1F	0.050847	0.242424	27.72176	1.40E-07	0.002665
MAPK15	0.088983	0.356061	38.3313	5.97E-10	1.14E-05
STAU2	0.084746	0.348485	38.23476	6.27E-10	1.19E-05
OSR2	0.09322	0.393939	45.79801	1.31E-11	2.49E-07
FA2H	0.110169	0.363636	32.29363	1.33E-08	0.000252
COQ9	0.050847	0.242424	27.72176	1.40E-07	0.002665
UBR5	0.09322	0.386364	43.92067	3.42E-11	6.51E-07
MROH6	0.088983	0.356061	38.3313	5.97E-10	1.14E-05
CYP7A1	0.080508	0.30303	29.5054	5.58E-08	0.001061
CTC-479C5.6	0.055085	0.242424	25.96278	3.48E-07	0.006622
GPIHBP1	0.088983	0.348485	36.55736	1.48E-09	2.82E-05
SLC7A6	0.059322	0.242424	24.30112	8.24E-07	0.015676
LONP2	0.029661	0.181818	23.47133	1.27E-06	0.024123
LRRCC1	0.101695	0.356061	33.56055	6.91E-09	0.000131
GOT2	0.055085	0.257576	29.36496	6.00E-08	0.001141
FAM96B	0.059322	0.234848	22.69198	1.90E-06	0.03618
DNAJC5B	0.084746	0.333333	34.70179	3.84E-09	7.31E-05
JPH1	0.088983	0.356061	38.3313	5.97E-10	1.14E-05
NUDT7	0.127119	0.356061	25.45059	4.54E-07	0.008635
OSGIN2	0.097458	0.371212	38.59102	5.23E-10	9.94E-06
NLRC5	0.050847	0.242424	27.72176	1.40E-07	0.002665
RAD21	0.101695	0.386364	40.53896	1.93E-10	3.67E-06
B3GNT9	0.055085	0.234848	24.30645	8.22E-07	0.015632
ATP6V1C1	0.09322	0.393939	45.79801	1.31E-11	2.49E-07
ZNF572	0.088983	0.393939	47.60756	5.21E-12	9.91E-08
BCAR1	0.114407	0.356061	29.2852	6.25E-08	0.001189
MTDH	0.09322	0.393939	45.79801	1.31E-11	2.49E-07
FAM110B	0.072034	0.30303	32.76502	1.04E-08	0.000198
ZFP41	0.088983	0.363636	40.13285	2.37E-10	4.51E-06
UBE2V2	0.072034	0.287879	29.35015	6.04E-08	0.001149
RRM2B	0.09322	0.386364	43.92067	3.42E-11	6.51E-07
ZBTB10	0.09322	0.356061	36.68223	1.39E-09	2.65E-05
LY6E	0.088983	0.363636	40.13285	2.37E-10	4.51E-06

**LYPD2**	0.088983	0.363636	40.13285	2.37E-10	4.51E-06
**SHARPIN**	0.088983	0.356061	38.3313	5.97E-10	1.14E-05
**HGH1**	0.088983	0.348485	36.55736	1.48E-09	2.82E-05
**LYNX1**	0.088983	0.363636	40.13285	2.37E-10	4.51E-06
**FNTA**	0.122881	0.333333	22.27248	2.37E-06	0.04501
**RFWD3**	0.105932	0.363636	33.75143	6.26E-09	0.000119
**WDR97**	0.088983	0.356061	38.3313	5.97E-10	1.14E-05
**TP53**	0.110169	0.318182	22.95415	1.66E-06	0.031566
**ZHX1**	0.088983	0.378788	43.81719	3.61E-11	6.86E-07
**TMEM71**	0.097458	0.393939	44.04758	3.20E-11	6.10E-07
**ZNRF1**	0.122881	0.356061	26.68283	2.40E-07	0.004561
**PSMB10**	0.055085	0.242424	25.96278	3.48E-07	0.006622
**CEBPD**	0.072034	0.280303	27.68668	1.43E-07	0.002714
**SULF1**	0.088983	0.348485	36.55736	1.48E-09	2.82E-05
**TRPS1**	0.097458	0.393939	44.04758	3.20E-11	6.10E-07
**C8orf44-SGK3**	0.084746	0.340909	36.45437	1.56E-09	2.97E-05
**LDHD**	0.118644	0.356061	27.96047	1.24E-07	0.002356
**TERF2IP**	0.122881	0.356061	26.68283	2.40E-07	0.004561
**DDX28**	0.055085	0.242424	25.96278	3.48E-07	0.006622
**CLEC3A**	0.131356	0.356061	24.2621	8.41E-07	0.015997
**RP11-26J3.4**	0.09322	0.356061	36.68223	1.39E-09	2.65E-05
**DRC3**	0.004237	0.121212	23.70053	1.13E-06	0.021414
**TERF1**	0.080508	0.356061	41.81861	1.00E-10	1.91E-06
**GNAO1**	0.050847	0.234848	26.01854	3.38E-07	0.006434
**PMP2**	0.088983	0.363636	40.13285	2.37E-10	4.51E-06
**PKHD1L1**	0.101695	0.401515	44.19669	2.97E-11	5.65E-07
**AC010287.1**	0.055085	0.257576	29.36496	6.00E-08	0.001141
**TCEA1**	0.067797	0.318182	38.10887	6.69E-10	1.27E-05
**ANXA13**	0.088983	0.378788	43.81719	3.61E-11	6.86E-07
**RSPO2**	0.097458	0.393939	44.04758	3.20E-11	6.10E-07
**SLC9A5**	0.055085	0.234848	24.30645	8.22E-07	0.015632
**TRHR**	0.105932	0.393939	40.71536	1.76E-10	3.35E-06
**PMFBP1**	0.110169	0.325758	24.43059	7.70E-07	0.014657
**RP11-240B13.2**	0.084746	0.393939	49.4786	2.01E-12	3.82E-08
**NPIPB15**	0.105932	0.348485	30.44512	3.43E-08	0.000653
**KBTBD11**	0.20339	0.454545	24.55565	7.22E-07	0.013736
**RRAD**	0.059322	0.234848	22.69198	1.90E-06	0.03618
**VCPIP1**	0.080508	0.340909	38.16239	6.51E-10	1.24E-05
**OC90**	0.084746	0.393939	49.4786	2.01E-12	3.82E-08
**GINS3**	0.050847	0.25	29.4537	5.73E-08	0.00109
**FABP4**	0.088983	0.363636	40.13285	2.37E-10	4.51E-06
**RABEP1**	0.050847	0.234848	26.01854	3.38E-07	0.006434
**KCNS2**	0.088983	0.401515	49.54176	1.94E-12	3.69E-08
**C8orf76**	0.088983	0.378788	43.81719	3.61E-11	6.86E-07

FABP5	0.088983	0.363636	40.13285	2.37E-10	4.51E-06
XKR9	0.084746	0.348485	38.23476	6.27E-10	1.19E-05
KIAA0196	0.088983	0.393939	47.60756	5.21E-12	9.91E-08
TSNARE1	0.088983	0.356061	38.3313	5.97E-10	1.14E-05
PSKH1	0.055085	0.242424	25.96278	3.48E-07	0.006622
MAFA	0.088983	0.363636	40.13285	2.37E-10	4.51E-06
HES7	0.067797	0.257576	24.37647	7.92E-07	0.015074
MLKL	0.110169	0.363636	32.29363	1.33E-08	0.000252
DPEP2	0.055085	0.242424	25.96278	3.48E-07	0.006622
FAM84B	0.088983	0.393939	47.60756	5.21E-12	9.91E-08
LRRC69	0.09322	0.363636	38.4505	5.62E-10	1.07E-05
ARL2BP	0.050847	0.242424	27.72176	1.40E-07	0.002665
PKD1L3	0.105932	0.310606	22.72338	1.87E-06	0.035593
NFATC3	0.055085	0.242424	25.96278	3.48E-07	0.006622
SMPD3	0.059322	0.242424	24.30112	8.24E-07	0.015676
CMTM3	0.059322	0.25	25.94183	3.52E-07	0.006695
GFOD2	0.055085	0.242424	25.96278	3.48E-07	0.006622
AC100821.2	0.067797	0.310606	36.29438	1.70E-09	3.23E-05
AGO2	0.088983	0.386364	45.69928	1.38E-11	2.62E-07
CHD7	0.09322	0.295455	23.59547	1.19E-06	0.022616
IMPAD1	0.072034	0.295455	31.04317	2.52E-08	0.00048
ESRP1	0.088983	0.371212	41.96161	9.31E-11	1.77E-06
SNAI2	0.072034	0.295455	31.04317	2.52E-08	0.00048
FBXO43	0.09322	0.393939	45.79801	1.31E-11	2.49E-07
KCNQ3	0.097458	0.393939	44.04758	3.20E-11	6.10E-07
E2F5	0.09322	0.356061	36.68223	1.39E-09	2.65E-05
SLC7A13	0.084746	0.363636	41.87726	9.72E-11	1.85E-06
GSDMC	0.084746	0.393939	49.4786	2.01E-12	3.82E-08
WWP1	0.088983	0.363636	40.13285	2.37E-10	4.51E-06
CMTM1	0.059322	0.25	25.94183	3.52E-07	0.006695
PCMTD1	0.067797	0.30303	34.5066	4.25E-09	8.08E-05
PER1	0.067797	0.25	22.79482	1.80E-06	0.034295
PARD6A	0.055085	0.234848	24.30645	8.22E-07	0.015632
NUTF2	0.055085	0.242424	25.96278	3.48E-07	0.006622
CDH17	0.09322	0.371212	40.24658	2.24E-10	4.26E-06
RAB2A	0.09322	0.295455	23.59547	1.19E-06	0.022616
ADCY8	0.084746	0.393939	49.4786	2.01E-12	3.82E-08
C16orf86	0.055085	0.234848	24.30645	8.22E-07	0.015632
CCDC79	0.059322	0.25	25.94183	3.52E-07	0.006695
ADAT1	0.118644	0.348485	26.42383	2.74E-07	0.005216
PTDSS1	0.09322	0.386364	43.92067	3.42E-11	6.51E-07
PSMD7	0.105932	0.348485	30.44512	3.43E-08	0.000653
ELP5	0.101695	0.30303	22.50389	2.10E-06	0.039901
MT1B	0.050847	0.242424	27.72176	1.40E-07	0.002665

**CCDC166**	0.088983	0.356061	38.3313	5.97E-10	1.14E-05
**CLVS1**	0.097458	0.30303	23.78495	1.08E-06	0.020495
**ABRA**	0.09322	0.401515	47.7018	4.96E-12	9.44E-08
**ACSF3**	0.122881	0.348485	25.17984	5.22E-07	0.009936
**BHLHE22**	0.076271	0.318182	34.55182	4.15E-09	7.90E-05
**ERICH5**	0.088983	0.393939	47.60756	5.21E-12	9.91E-08
**TNFRSF11B**	0.09322	0.386364	43.92067	3.42E-11	6.51E-07
**COLEC10**	0.097458	0.386364	42.20132	8.23E-11	1.57E-06
**CYP7B1**	0.080508	0.325758	34.6147	4.02E-09	7.65E-05
**MTBP**	0.09322	0.378788	42.07009	8.81E-11	1.68E-06
**ITFG1**	0.029661	0.181818	23.47133	1.27E-06	0.024123
**LYPLA1**	0.067797	0.310606	36.29438	1.70E-09	3.23E-05
**GID4**	0.004237	0.121212	23.70053	1.13E-06	0.021414
**NDRG1**	0.09322	0.393939	45.79801	1.31E-11	2.49E-07
**RB1CC1**	0.063559	0.318182	40.00423	2.53E-10	4.82E-06
**TMEM74**	0.101695	0.393939	42.35407	7.62E-11	1.45E-06
**LCAT**	0.055085	0.242424	25.96278	3.48E-07	0.006622
**INTS8**	0.088983	0.378788	43.81719	3.61E-11	6.86E-07
**TMEM170A**	0.118644	0.356061	27.96047	1.24E-07	0.002356
**RP11-403P17.5**	0.059322	0.25	25.94183	3.52E-07	0.006695
**USP6**	0.04661	0.227273	26.11359	3.22E-07	0.006125
**RP11-77K12.7**	0.118644	0.356061	27.96047	1.24E-07	0.002356
**STMN2**	0.09322	0.348485	34.94219	3.40E-09	6.46E-05
**TAF2**	0.097458	0.378788	40.38235	2.09E-10	3.97E-06
**LRRC36**	0.055085	0.234848	24.30645	8.22E-07	0.015632
**KARS**	0.122881	0.348485	25.17984	5.22E-07	0.009936
**PRDM14**	0.084746	0.356061	40.04253	2.48E-10	4.73E-06
**HP**	0.101695	0.318182	25.50461	4.41E-07	0.008397
**C8orf88**	0.09322	0.363636	38.4505	5.62E-10	1.07E-05
**CES3**	0.059322	0.234848	22.69198	1.90E-06	0.03618
**TRAPPC2L**	0.122881	0.348485	25.17984	5.22E-07	0.009936
**ZNF696**	0.088983	0.363636	40.13285	2.37E-10	4.51E-06
**SDCBP**	0.080508	0.30303	29.5054	5.58E-08	0.001061
**KATNB1**	0.050847	0.242424	27.72176	1.40E-07	0.002665
**CTRB2**	0.114407	0.356061	29.2852	6.25E-08	0.001189
**ENY2**	0.101695	0.393939	42.35407	7.62E-11	1.45E-06
**TPPP3**	0.055085	0.234848	24.30645	8.22E-07	0.015632
**C16orf70**	0.055085	0.234848	24.30645	8.22E-07	0.015632
**SCIMP**	0.04661	0.227273	26.11359	3.22E-07	0.006125
**CKLF**	0.059322	0.25	25.94183	3.52E-07	0.006695
**EDC4**	0.055085	0.242424	25.96278	3.48E-07	0.006622
**MOS**	0.067797	0.295455	32.74609	1.05E-08	0.0002
**FZD6**	0.09322	0.393939	45.79801	1.31E-11	2.49E-07
**SNTB1**	0.09322	0.378788	42.07009	8.81E-11	1.68E-06

BBS2	0.050847	0.25	29.4537	5.73E-08	0.00109
BEAN1	0.059322	0.25	25.94183	3.52E-07	0.006695
FAM92A1	0.09322	0.371212	40.24658	2.24E-10	4.26E-06
MT1M	0.050847	0.242424	27.72176	1.40E-07	0.002665
ZNF319	0.050847	0.242424	27.72176	1.40E-07	0.002665
TK2	0.059322	0.25	25.94183	3.52E-07	0.006695
PPP1R42	0.084746	0.340909	36.45437	1.56E-09	2.97E-05
TG	0.101695	0.393939	42.35407	7.62E-11	1.45E-06
DOK4	0.055085	0.242424	25.96278	3.48E-07	0.006622
HERPUD1	0.050847	0.242424	27.72176	1.40E-07	0.002665
ZNF469	0.148305	0.371212	22.6286	1.97E-06	0.037393
CMTM4	0.059322	0.25	25.94183	3.52E-07	0.006695
CHMP4C	0.088983	0.356061	38.3313	5.97E-10	1.14E-05
CHST5	0.118644	0.356061	27.96047	1.24E-07	0.002356
PSCA	0.088983	0.363636	40.13285	2.37E-10	4.51E-06
CHST4	0.097458	0.295455	22.29626	2.34E-06	0.044456
RPL7	0.084746	0.348485	38.23476	6.27E-10	1.19E-05
CHST6	0.118644	0.356061	27.96047	1.24E-07	0.002356
RGS20	0.067797	0.310606	36.29438	1.70E-09	3.23E-05
KLHL38	0.088983	0.378788	43.81719	3.61E-11	6.86E-07
VAT1L	0.131356	0.348485	22.82601	1.77E-06	0.033743
POU5F1B	0.088983	0.401515	49.54176	1.94E-12	3.69E-08
ATP6V0D1	0.055085	0.234848	24.30645	8.22E-07	0.015632
TMEM68	0.076271	0.30303	31.09919	2.45E-08	0.000466
RP11-1099M24.7	0.101695	0.310606	23.98776	9.70E-07	0.018446
PAG1	0.09322	0.363636	38.4505	5.62E-10	1.07E-05
FAM135B	0.101695	0.386364	40.53896	1.93E-10	3.67E-06
GGH	0.080508	0.30303	29.5054	5.58E-08	0.001061
RP1	0.072034	0.30303	32.76502	1.04E-08	0.000198
ENPP2	0.097458	0.386364	42.20132	8.23E-11	1.57E-06
TSTA3	0.088983	0.348485	36.55736	1.48E-09	2.82E-05
MT1X	0.050847	0.234848	26.01854	3.38E-07	0.006434
AC004158.1	0.110169	0.348485	29.05459	7.04E-08	0.001339
SDC2	0.088983	0.386364	45.69928	1.38E-11	2.62E-07
RNF166	0.122881	0.348485	25.17984	5.22E-07	0.009936
NOV	0.097458	0.386364	42.20132	8.23E-11	1.57E-06
UBE2W	0.084746	0.348485	38.23476	6.27E-10	1.19E-05
ARFGEF1	0.084746	0.340909	36.45437	1.56E-09	2.97E-05
DCAF13	0.09322	0.393939	45.79801	1.31E-11	2.49E-07
CCNE2	0.088983	0.378788	43.81719	3.61E-11	6.86E-07
RPL26	0.055085	0.227273	22.68139	1.91E-06	0.03638
RP11-849F2.7	0.055085	0.227273	22.68139	1.91E-06	0.03638
GALNS	0.122881	0.348485	25.17984	5.22E-07	0.009936
SPATC1	0.088983	0.356061	38.3313	5.97E-10	1.14E-05

**OTUD6B**	0.09322	0.363636	38.4505	5.62E-10	1.07E-05
**E2F4**	0.055085	0.242424	25.96278	3.48E-07	0.006622
**EIF3E**	0.105932	0.393939	40.71536	1.76E-10	3.35E-06
**ZNF623**	0.088983	0.356061	38.3313	5.97E-10	1.14E-05
**DHX38**	0.105932	0.325758	25.7159	3.96E-07	0.007526
**PENK**	0.072034	0.295455	31.04317	2.52E-08	0.00048
**PUF60**	0.088983	0.356061	38.3313	5.97E-10	1.14E-05
**MRPL15**	0.067797	0.310606	36.29438	1.70E-09	3.23E-05
**DCSTAMP**	0.09322	0.409091	49.63179	1.85E-12	3.53E-08
**FAM83A**	0.088983	0.378788	43.81719	3.61E-11	6.86E-07
**PDP1**	0.09322	0.371212	40.24658	2.24E-10	4.26E-06
**EYA1**	0.084746	0.348485	38.23476	6.27E-10	1.19E-05
**EXOSC4**	0.084746	0.356061	40.04253	2.48E-10	4.73E-06
**TEPP**	0.050847	0.242424	27.72176	1.40E-07	0.002665
**RP11-598P20.5**	0.122881	0.348485	25.17984	5.22E-07	0.009936
**MAF**	0.135593	0.356061	23.11585	1.53E-06	0.02902
**AC087350.1**	0.101695	0.386364	40.53896	1.93E-10	3.67E-06
**SLA**	0.097458	0.393939	44.04758	3.20E-11	6.10E-07
**RP11-343C2.11**	0.067797	0.257576	24.37647	7.92E-07	0.015074
**EPPK1**	0.088983	0.363636	40.13285	2.37E-10	4.51E-06
**FABP12**	0.088983	0.356061	38.3313	5.97E-10	1.14E-05
**MED30**	0.09322	0.393939	45.79801	1.31E-11	2.49E-07
**FAM192A**	0.050847	0.242424	27.72176	1.40E-07	0.002665
**ZFP90**	0.063559	0.242424	22.73077	1.86E-06	0.035457
**NDUFAF6**	0.088983	0.378788	43.81719	3.61E-11	6.86E-07
**KHDRBS3**	0.097458	0.393939	44.04758	3.20E-11	6.10E-07
**MAL2**	0.097458	0.386364	42.20132	8.23E-11	1.57E-06
**AC010547.9**	0.097458	0.295455	22.29626	2.34E-06	0.044456
**NECAB1**	0.09322	0.363636	38.4505	5.62E-10	1.07E-05
**COPS5**	0.084746	0.340909	36.45437	1.56E-09	2.97E-05
**ADGRG1**	0.050847	0.242424	27.72176	1.40E-07	0.002665
**PREX2**	0.097458	0.348485	33.3867	7.55E-09	0.000144
**AFAP1L2**	0.008475	0.128788	22.62863	1.97E-06	0.037393
**MT1E**	0.050847	0.242424	27.72176	1.40E-07	0.002665
**NAA38**	0.105932	0.310606	22.72338	1.87E-06	0.035593
**AZIN1**	0.09322	0.393939	45.79801	1.31E-11	2.49E-07
**EIF3H**	0.097458	0.386364	42.20132	8.23E-11	1.57E-06
**CSNK2A2**	0.050847	0.242424	27.72176	1.40E-07	0.002665
**PDE7A**	0.080508	0.333333	36.37475	1.63E-09	3.10E-05
**DERL2**	0.050847	0.219697	22.70249	1.89E-06	0.035982
**RANBP10**	0.055085	0.242424	25.96278	3.48E-07	0.006622
**ZHX1-C8orf76**	0.088983	0.378788	43.81719	3.61E-11	6.86E-07
**RP11-599B13.6**	0.067797	0.25	22.79482	1.80E-06	0.034295
**RNF139**	0.088983	0.386364	45.69928	1.38E-11	2.62E-07

**WDYHV1**	0.088983	0.378788	43.81719	3.61E-11	6.86E-07
**IMPA1**	0.088983	0.356061	38.3313	5.97E-10	1.14E-05
**NDRG4**	0.050847	0.257576	31.21344	2.31E-08	0.00044
**CPNE3**	0.09322	0.363636	38.4505	5.62E-10	1.07E-05
**KCNB2**	0.088983	0.356061	38.3313	5.97E-10	1.14E-05
**FAM83H**	0.088983	0.356061	38.3313	5.97E-10	1.14E-05
**CCL22**	0.055085	0.242424	25.96278	3.48E-07	0.006622
**JRK**	0.088983	0.363636	40.13285	2.37E-10	4.51E-06
**GDAP1**	0.088983	0.371212	41.96161	9.31E-11	1.77E-06
**CRH**	0.080508	0.340909	38.16239	6.51E-10	1.24E-05
**NAPRT**	0.088983	0.356061	38.3313	5.97E-10	1.14E-05
**RAP1GAP2**	0.042373	0.212121	24.54063	7.28E-07	0.013843
**TMEM107**	0.067797	0.25	22.79482	1.80E-06	0.034295
**TSNAXIP1**	0.055085	0.242424	25.96278	3.48E-07	0.006622
**MROH1**	0.088983	0.356061	38.3313	5.97E-10	1.14E-05
**TMEM65**	0.088983	0.393939	47.60756	5.21E-12	9.91E-08
**AC129492.1**	0.067797	0.25	22.79482	1.80E-06	0.034295
**NKAIN3**	0.084746	0.30303	27.98021	1.23E-07	0.002332
**NUDCD1**	0.101695	0.393939	42.35407	7.62E-11	1.45E-06
**KLF10**	0.09322	0.393939	45.79801	1.31E-11	2.49E-07
**UQCRB**	0.09322	0.386364	43.92067	3.42E-11	6.51E-07
**NSMCE2**	0.088983	0.401515	49.54176	1.94E-12	3.69E-08
**CX3CL1**	0.055085	0.242424	25.96278	3.48E-07	0.006622
**ZFP1**	0.114407	0.356061	29.2852	6.25E-08	0.001189
**KRBA2**	0.055085	0.227273	22.68139	1.91E-06	0.03638
**SNX31**	0.097458	0.393939	44.04758	3.20E-11	6.10E-07
**KIFC3**	0.050847	0.242424	27.72176	1.40E-07	0.002665
**RP11-343C2.9**	0.067797	0.257576	24.37647	7.92E-07	0.015074
**MYBL1**	0.080508	0.340909	38.16239	6.51E-10	1.24E-05
**SGK3**	0.084746	0.340909	36.45437	1.56E-09	2.97E-05
**LY96**	0.084746	0.348485	38.23476	6.27E-10	1.19E-05
**MATN2**	0.088983	0.393939	47.60756	5.21E-12	9.91E-08
**RPS20**	0.072034	0.295455	31.04317	2.52E-08	0.00048
**TMEM88**	0.105932	0.310606	22.72338	1.87E-06	0.035593
**PABPN1L**	0.122881	0.348485	25.17984	5.22E-07	0.009936
**EFR3A**	0.084746	0.401515	51.44303	7.37E-13	1.40E-08
**FTO**	0.055085	0.234848	24.30645	8.22E-07	0.015632
**NRBP2**	0.088983	0.363636	40.13285	2.37E-10	4.51E-06
**LYN**	0.067797	0.295455	32.74609	1.05E-08	0.0002
**RPL30**	0.088983	0.393939	47.60756	5.21E-12	9.91E-08
**NCALD**	0.088983	0.393939	47.60756	5.21E-12	9.91E-08
**MTFR1**	0.080508	0.325758	34.6147	4.02E-09	7.65E-05
**NDUFB9**	0.088983	0.393939	47.60756	5.21E-12	9.91E-08
**CTU2**	0.122881	0.348485	25.17984	5.22E-07	0.009936

C8orf46	0.084746	0.340909	36.45437	1.56E-09	2.97E-05
DHODH	0.101695	0.310606	23.98776	9.70E-07	0.018446
CFAP20	0.050847	0.242424	27.72176	1.40E-07	0.002665
CLDN7	0.101695	0.30303	22.50389	2.10E-06	0.039901
ADGRG3	0.050847	0.242424	27.72176	1.40E-07	0.002665
KCNV1	0.088983	0.409091	51.50162	7.15E-13	1.36E-08
SAMD12	0.110169	0.393939	39.12945	3.97E-10	7.55E-06
MRPS28	0.09322	0.356061	36.68223	1.39E-09	2.65E-05
AARD	0.101695	0.386364	40.53896	1.93E-10	3.67E-06
TOX3	0.059322	0.234848	22.69198	1.90E-06	0.03618
FSBP	0.09322	0.371212	40.24658	2.24E-10	4.26E-06
WDR59	0.122881	0.371212	29.7847	4.83E-08	0.000919
CES2	0.059322	0.234848	22.69198	1.90E-06	0.03618
DSCC1	0.097458	0.378788	40.38235	2.09E-10	3.97E-06
CA13	0.09322	0.356061	36.68223	1.39E-09	2.65E-05
WISP1	0.097458	0.393939	44.04758	3.20E-11	6.10E-07
RPAIN	0.050847	0.219697	22.70249	1.89E-06	0.035982
RP11-457D20.2	0.050847	0.265152	33.00016	9.22E-09	0.000175
DENND3	0.088983	0.378788	43.81719	3.61E-11	6.86E-07
NSMAF	0.080508	0.30303	29.5054	5.58E-08	0.001061
SCRIB	0.088983	0.356061	38.3313	5.97E-10	1.14E-05
GUCY2D	0.097458	0.295455	22.29626	2.34E-06	0.044456
XKR4	0.076271	0.30303	31.09919	2.45E-08	0.000466
RP11-463D19.2	0.084746	0.348485	38.23476	6.27E-10	1.19E-05
HNF4G	0.097458	0.356061	35.09278	3.14E-09	5.98E-05
CNOT1	0.055085	0.257576	29.36496	6.00E-08	0.001141
DYNLRB2	0.131356	0.348485	22.82601	1.77E-06	0.033743
IL17C	0.122881	0.348485	25.17984	5.22E-07	0.009936
CMTR2	0.097458	0.295455	22.29626	2.34E-06	0.044456
PKIA	0.09322	0.348485	34.94219	3.40E-09	6.46E-05
LAPTM4B	0.088983	0.393939	47.60756	5.21E-12	9.91E-08
C8orf22	0.067797	0.280303	29.30944	6.17E-08	0.001174
ASAP1	0.088983	0.401515	49.54176	1.94E-12	3.69E-08
CSPP1	0.084746	0.340909	36.45437	1.56E-09	2.97E-05
ARHGEF10	0.216102	0.469697	24.40775	7.80E-07	0.014832
NUP88	0.050847	0.227273	24.34508	8.05E-07	0.015322
SLC45A4	0.088983	0.378788	43.81719	3.61E-11	6.86E-07
VPS4A	0.067797	0.257576	24.37647	7.92E-07	0.015074
FAM49B	0.084746	0.393939	49.4786	2.01E-12	3.82E-08
BAALC	0.09322	0.393939	45.79801	1.31E-11	2.49E-07
CES4A	0.059322	0.234848	22.69198	1.90E-06	0.03618
CKLF-CMTM1	0.059322	0.25	25.94183	3.52E-07	0.006695
TCEB1	0.084746	0.348485	38.23476	6.27E-10	1.19E-05
TRADD	0.055085	0.234848	24.30645	8.22E-07	0.015632

**CHCHD7**	0.067797	0.295455	32.74609	1.05E-08	0.0002
**CRISPLD1**	0.084746	0.356061	40.04253	2.48E-10	4.73E-06
**LRRC6**	0.09322	0.393939	45.79801	1.31E-11	2.49E-07
**ZFHX4**	0.097458	0.363636	36.8277	1.29E-09	2.46E-05
**CFDP1**	0.127119	0.356061	25.45059	4.54E-07	0.008635
**ACD**	0.055085	0.234848	24.30645	8.22E-07	0.015632
**NIP7**	0.067797	0.257576	24.37647	7.92E-07	0.015074
**RUNX1T1**	0.09322	0.371212	40.24658	2.24E-10	4.26E-06
**ATPAF2**	0.004237	0.121212	23.70053	1.13E-06	0.021414
**CHRAC1**	0.088983	0.378788	43.81719	3.61E-11	6.86E-07
**MTSS1**	0.088983	0.401515	49.54176	1.94E-12	3.69E-08
**ARMC1**	0.080508	0.325758	34.6147	4.02E-09	7.65E-05
**DCAF4L2**	0.084746	0.356061	40.04253	2.48E-10	4.73E-06
**TATDN1**	0.088983	0.393939	47.60756	5.21E-12	9.91E-08
**MRPL13**	0.09322	0.378788	42.07009	8.81E-11	1.68E-06
**TMEM208**	0.055085	0.242424	25.96278	3.48E-07	0.006622
**RP11-58C22.1**	0.127119	0.363636	26.95268	2.08E-07	0.003967
**CLN8**	0.20339	0.454545	24.55565	7.22E-07	0.013736
**ZFP3**	0.042373	0.219697	26.2533	2.99E-07	0.005697
**FAM65A**	0.055085	0.234848	24.30645	8.22E-07	0.015632
**TRIB1**	0.088983	0.393939	47.60756	5.21E-12	9.91E-08
**CCDC113**	0.050847	0.242424	27.72176	1.40E-07	0.002665
**ALOX15B**	0.09322	0.295455	23.59547	1.19E-06	0.022616
**COX6C**	0.088983	0.393939	47.60756	5.21E-12	9.91E-08
**SLC12A4**	0.055085	0.242424	25.96278	3.48E-07	0.006622
**SLCO5A1**	0.09322	0.348485	34.94219	3.40E-09	6.46E-05
**ZNF707**	0.088983	0.356061	38.3313	5.97E-10	1.14E-05
**TMED6**	0.067797	0.257576	24.37647	7.92E-07	0.015074
**ZDHHC1**	0.055085	0.234848	24.30645	8.22E-07	0.015632
**LRP12**	0.09322	0.409091	49.63179	1.85E-12	3.53E-08
**NIPAL2**	0.088983	0.393939	47.60756	5.21E-12	9.91E-08
**TRAPPC9**	0.110169	0.378788	35.65305	2.36E-09	4.49E-05
**MT2A**	0.050847	0.242424	27.72176	1.40E-07	0.002665
**EMC2**	0.101695	0.393939	42.35407	7.62E-11	1.45E-06
**AC007906.1**	0.055085	0.227273	22.68139	1.91E-06	0.03638
**SLC22A31**	0.122881	0.348485	25.17984	5.22E-07	0.009936
**CYBA**	0.122881	0.348485	25.17984	5.22E-07	0.009936
**HSF4**	0.055085	0.234848	24.30645	8.22E-07	0.015632
**UBXN2B**	0.080508	0.30303	29.5054	5.58E-08	0.001061
**CTHRC1**	0.09322	0.401515	47.7018	4.96E-12	9.44E-08
**CA8**	0.084746	0.287879	24.80131	6.36E-07	0.012092
**YWHAZ**	0.09322	0.401515	47.7018	4.96E-12	9.44E-08
**CCDC102A**	0.050847	0.242424	27.72176	1.40E-07	0.002665
**HRSP12**	0.088983	0.393939	47.60756	5.21E-12	9.91E-08

**SETD6**	0.050847	0.257576	31.21344	2.31E-08	0.00044
**PTK2**	0.088983	0.378788	43.81719	3.61E-11	6.86E-07
**LPCAT2**	0.055085	0.234848	24.30645	8.22E-07	0.015632
**TMEM64**	0.088983	0.363636	40.13285	2.37E-10	4.51E-06
**RDH10**	0.084746	0.348485	38.23476	6.27E-10	1.19E-05
**LRRC29**	0.055085	0.242424	25.96278	3.48E-07	0.006622
**MT1H**	0.050847	0.242424	27.72176	1.40E-07	0.002665
**SLC6A2**	0.050847	0.227273	24.34508	8.05E-07	0.015322
**PYCRL**	0.088983	0.356061	38.3313	5.97E-10	1.14E-05
**CALB1**	0.097458	0.363636	36.8277	1.29E-09	2.46E-05
**PEX2**	0.097458	0.356061	35.09278	3.14E-09	5.98E-05
**CETP**	0.050847	0.242424	27.72176	1.40E-07	0.002665
**RNF19A**	0.09322	0.393939	45.79801	1.31E-11	2.49E-07
**GDF6**	0.097458	0.386364	42.20132	8.23E-11	1.57E-06
**GPAA1**	0.088983	0.356061	38.3313	5.97E-10	1.14E-05
**TCF24**	0.084746	0.340909	36.45437	1.56E-09	2.97E-05
**EEF1D**	0.088983	0.356061	38.3313	5.97E-10	1.14E-05
**ZNF23**	0.097458	0.295455	22.29626	2.34E-06	0.044456
**MT1A**	0.055085	0.25	27.64928	1.45E-07	0.002767
**AMFR**	0.050847	0.25	29.4537	5.73E-08	0.00109
**AC104581.1**	0.105932	0.310606	22.72338	1.87E-06	0.035593
**RHPN1**	0.09322	0.363636	38.4505	5.62E-10	1.07E-05
**KIAA1429**	0.09322	0.363636	38.4505	5.62E-10	1.07E-05
**ATAD2**	0.09322	0.378788	42.07009	8.81E-11	1.68E-06
**TRIM55**	0.080508	0.340909	38.16239	6.51E-10	1.24E-05
**IRX3**	0.050847	0.242424	27.72176	1.40E-07	0.002665
**DUS2**	0.055085	0.242424	25.96278	3.48E-07	0.006622
**FAM91A1**	0.088983	0.386364	45.69928	1.38E-11	2.62E-07
**GLG1**	0.110169	0.363636	32.29363	1.33E-08	0.000252
**MYC**	0.088983	0.401515	49.54176	1.94E-12	3.69E-08
**ASPH**	0.084746	0.30303	27.98021	1.23E-07	0.002332
**C8orf44**	0.080508	0.340909	38.16239	6.51E-10	1.24E-05
**ZC2HC1A**	0.09322	0.348485	34.94219	3.40E-09	6.46E-05
**ZNF594**	0.04661	0.227273	26.11359	3.22E-07	0.006125
**C8orf34**	0.084746	0.340909	36.45437	1.56E-09	2.97E-05
**FER1L6**	0.088983	0.393939	47.60756	5.21E-12	9.91E-08
**OPLAH**	0.088983	0.356061	38.3313	5.97E-10	1.14E-05
**MT1G**	0.050847	0.242424	27.72176	1.40E-07	0.002665
**GML**	0.088983	0.363636	40.13285	2.37E-10	4.51E-06
**ANKRD11**	0.122881	0.340909	23.70953	1.12E-06	0.021314
**STK3**	0.097458	0.393939	44.04758	3.20E-11	6.10E-07
**SYCE1L**	0.127119	0.356061	25.45059	4.54E-07	0.008635
**CTRL**	0.055085	0.242424	25.96278	3.48E-07	0.006622
**AGRP**	0.055085	0.234848	24.30645	8.22E-07	0.015632

**ALOX12B**	0.067797	0.265152	25.99009	3.43E-07	0.006529
**DRG2**	0.004237	0.121212	23.70053	1.13E-06	0.021414
**CNTROB**	0.101695	0.310606	23.98776	9.70E-07	0.018446
**TMEM70**	0.084746	0.348485	38.23476	6.27E-10	1.19E-05
**SLC38A7**	0.059322	0.257576	27.61304	1.48E-07	0.002819
**EFCAB1**	0.072034	0.287879	29.35015	6.04E-08	0.001149
**ZFPM2**	0.110169	0.393939	39.12945	3.97E-10	7.55E-06
**ABCC12**	0.029661	0.181818	23.47133	1.27E-06	0.024123
**ZHX2**	0.09322	0.386364	43.92067	3.42E-11	6.51E-07
**SNTB2**	0.072034	0.257576	22.88168	1.72E-06	0.032779
**CYP11B2**	0.088983	0.363636	40.13285	2.37E-10	4.51E-06
**OGFOD1**	0.050847	0.25	29.4537	5.73E-08	0.00109
**DEPTOR**	0.097458	0.378788	40.38235	2.09E-10	3.97E-06
**PLA2G15**	0.059322	0.242424	24.30112	8.24E-07	0.015676
**PHF23**	0.097458	0.295455	22.29626	2.34E-06	0.044456
**ATP6V0D2**	0.088983	0.363636	40.13285	2.37E-10	4.51E-06
**CNBD1**	0.09322	0.363636	38.4505	5.62E-10	1.07E-05
**MCM4**	0.072034	0.280303	27.68668	1.43E-07	0.002714
**TOP1MT**	0.088983	0.363636	40.13285	2.37E-10	4.51E-06
**VPS35**	0.029661	0.181818	23.47133	1.27E-06	0.024123
**LY6K**	0.088983	0.363636	40.13285	2.37E-10	4.51E-06
**SDR16C5**	0.067797	0.295455	32.74609	1.05E-08	0.0002
**CPNE2**	0.055085	0.242424	25.96278	3.48E-07	0.006622
**THEM6**	0.088983	0.363636	40.13285	2.37E-10	4.51E-06
**CDH15**	0.122881	0.348485	25.17984	5.22E-07	0.009936
**DERL1**	0.09322	0.378788	42.07009	8.81E-11	1.68E-06
**IRX5**	0.055085	0.227273	22.68139	1.91E-06	0.03638
**RP11-343C2.12**	0.067797	0.257576	24.37647	7.92E-07	0.015074
**SOX17**	0.067797	0.30303	34.5066	4.25E-09	8.08E-05
**VAMP2**	0.067797	0.25	22.79482	1.80E-06	0.034295
**ADGRG5**	0.050847	0.242424	27.72176	1.40E-07	0.002665
**TPD52**	0.097458	0.356061	35.09278	3.14E-09	5.98E-05
**PDF**	0.067797	0.257576	24.37647	7.92E-07	0.015074
**N4BP1**	0.029661	0.181818	23.47133	1.27E-06	0.024123
**RP11-249C24.12**	0.050847	0.242424	27.72176	1.40E-07	0.002665
**CDH16**	0.059322	0.234848	22.69198	1.90E-06	0.03618
**RP11-77K12.1**	0.131356	0.356061	24.2621	8.41E-07	0.015997
**RGS22**	0.097458	0.393939	44.04758	3.20E-11	6.10E-07
**SPIDR**	0.076271	0.287879	27.76286	1.37E-07	0.002609
**RNF222**	0.055085	0.227273	22.68139	1.91E-06	0.03638
**CYB5B**	0.072034	0.257576	22.88168	1.72E-06	0.032779
**CNGB3**	0.101695	0.363636	35.26208	2.88E-09	5.48E-05
**TRAPPC1**	0.101695	0.310606	23.98776	9.70E-07	0.018446
**SLURP1**	0.088983	0.363636	40.13285	2.37E-10	4.51E-06

RALYL	0.097458	0.371212	38.59102	5.23E-10	9.94E-06
ELMO3	0.055085	0.242424	25.96278	3.48E-07	0.006622
PLAG1	0.067797	0.30303	34.5066	4.25E-09	8.08E-05
ZNF232	0.04661	0.219697	24.42137	7.74E-07	0.014727
HSD11B2	0.055085	0.234848	24.30645	8.22E-07	0.015632
ZFAND1	0.088983	0.356061	38.3313	5.97E-10	1.14E-05
PHF20L1	0.101695	0.393939	42.35407	7.62E-11	1.45E-06
CNGB1	0.050847	0.242424	27.72176	1.40E-07	0.002665
ZBTB16	0.021186	0.159091	22.46305	2.14E-06	0.040759
YTHDF3	0.076271	0.30303	31.09919	2.45E-08	0.000466
PRMT7	0.059322	0.242424	24.30112	8.24E-07	0.015676
GSDMD	0.088983	0.356061	38.3313	5.97E-10	1.14E-05
FBXO32	0.088983	0.378788	43.81719	3.61E-11	6.86E-07
BORCS6	0.067797	0.25	22.79482	1.80E-06	0.034295
PLEKHF2	0.088983	0.378788	43.81719	3.61E-11	6.86E-07
CYB5D1	0.105932	0.310606	22.72338	1.87E-06	0.035593
DPEP3	0.055085	0.242424	25.96278	3.48E-07	0.006622
RP13-582O9.6	0.088983	0.363636	40.13285	2.37E-10	4.51E-06
CMTM2	0.059322	0.25	25.94183	3.52E-07	0.006695
ZC3H18	0.122881	0.348485	25.17984	5.22E-07	0.009936
EBAG9	0.097458	0.393939	44.04758	3.20E-11	6.10E-07
C8orf59	0.09322	0.356061	36.68223	1.39E-09	2.65E-05
PIEZO1	0.122881	0.348485	25.17984	5.22E-07	0.009936
RAD54B	0.09322	0.371212	40.24658	2.24E-10	4.26E-06
HEY1	0.09322	0.348485	34.94219	3.40E-09	6.46E-05
FBXL8	0.055085	0.234848	24.30645	8.22E-07	0.015632
CA3	0.09322	0.356061	36.68223	1.39E-09	2.65E-05
ZNF778	0.122881	0.356061	26.68283	2.40E-07	0.004561
HHLA1	0.088983	0.393939	47.60756	5.21E-12	9.91E-08
SNAI3	0.122881	0.348485	25.17984	5.22E-07	0.009936
ZNF704	0.09322	0.356061	36.68223	1.39E-09	2.65E-05
TRIQK	0.09322	0.371212	40.24658	2.24E-10	4.26E-06
MTERF3	0.09322	0.386364	43.92067	3.42E-11	6.51E-07
ATP6V1H	0.067797	0.318182	38.10887	6.69E-10	1.27E-05
CBFA2T3	0.122881	0.348485	25.17984	5.22E-07	0.009936
GEM	0.09322	0.371212	40.24658	2.24E-10	4.26E-06
POLR2C	0.050847	0.242424	27.72176	1.40E-07	0.002665
DRC7	0.050847	0.242424	27.72176	1.40E-07	0.002665
ESRP2	0.055085	0.242424	25.96278	3.48E-07	0.006622
MMP2	0.050847	0.227273	24.34508	8.05E-07	0.015322
HAS2	0.097458	0.378788	40.38235	2.09E-10	3.97E-06
SLC26A7	0.09322	0.371212	40.24658	2.24E-10	4.26E-06
C8orf37	0.09322	0.378788	42.07009	8.81E-11	1.68E-06
YBX2	0.105932	0.310606	22.72338	1.87E-06	0.035593

PLEKHG4	0.055085	0.234848	24.30645	8.22E-07	0.015632
EXT1	0.101695	0.393939	42.35407	7.62E-11	1.45E-06
MT3	0.050847	0.242424	27.72176	1.40E-07	0.002665
CLEC18B	0.105932	0.348485	30.44512	3.43E-08	0.000653
RMDN1	0.088983	0.363636	40.13285	2.37E-10	4.51E-06
RBM12B	0.09322	0.371212	40.24658	2.24E-10	4.26E-06
PSKH2	0.088983	0.363636	40.13285	2.37E-10	4.51E-06
KCNAB3	0.105932	0.310606	22.72338	1.87E-06	0.035593
KCNK9	0.097458	0.378788	40.38235	2.09E-10	3.97E-06
TXNL4B	0.105932	0.325758	25.7159	3.96E-07	0.007526
COL22A1	0.09322	0.378788	42.07009	8.81E-11	1.68E-06
ZC3H3	0.088983	0.363636	40.13285	2.37E-10	4.51E-06
TMEM55A	0.09322	0.363636	38.4505	5.62E-10	1.07E-05
AC105009.1	0.088983	0.378788	43.81719	3.61E-11	6.86E-07
DYNC1LI2	0.059322	0.25	25.94183	3.52E-07	0.006695
THAP11	0.055085	0.242424	25.96278	3.48E-07	0.006622
NRN1L	0.055085	0.242424	25.96278	3.48E-07	0.006622
ST18	0.063559	0.318182	40.00423	2.53E-10	4.82E-06
CES1	0.050847	0.234848	26.01854	3.38E-07	0.006434
PLLP	0.055085	0.242424	25.96278	3.48E-07	0.006622
CTRB1	0.114407	0.356061	29.2852	6.25E-08	0.001189
PXDNL	0.080508	0.310606	31.17944	2.35E-08	0.000448
ZFAT	0.097458	0.393939	44.04758	3.20E-11	6.10E-07
ST3GAL1	0.097458	0.393939	44.04758	3.20E-11	6.10E-07
TP53INP1	0.088983	0.378788	43.81719	3.61E-11	6.86E-07
COG8	0.067797	0.257576	24.37647	7.92E-07	0.015074
CENPT	0.055085	0.242424	25.96278	3.48E-07	0.006622
HYDIN	0.097458	0.295455	22.29626	2.34E-06	0.044456
TOX	0.084746	0.318182	31.28212	2.23E-08	0.000425
CDH5	0.055085	0.25	27.64928	1.45E-07	0.002767
PARP10	0.088983	0.363636	40.13285	2.37E-10	4.51E-06
APRT	0.122881	0.348485	25.17984	5.22E-07	0.009936
RRS1	0.084746	0.340909	36.45437	1.56E-09	2.97E-05
RIMS2	0.105932	0.401515	42.52727	6.97E-11	1.33E-06
CPQ	0.09322	0.393939	45.79801	1.31E-11	2.49E-07
MARVELD3	0.097458	0.295455	22.29626	2.34E-06	0.044456
ODF1	0.09322	0.393939	45.79801	1.31E-11	2.49E-07
VPS13B	0.09322	0.393939	45.79801	1.31E-11	2.49E-07
SBSPON	0.084746	0.348485	38.23476	6.27E-10	1.19E-05
PRSS54	0.050847	0.242424	27.72176	1.40E-07	0.002665
PLEC	0.088983	0.363636	40.13285	2.37E-10	4.51E-06
ANKRD46	0.09322	0.393939	45.79801	1.31E-11	2.49E-07
ERICH1	0.207627	0.454545	23.58762	1.19E-06	0.022708
CBFB	0.059322	0.234848	22.69198	1.90E-06	0.03618

**AC138028.1**	0.122881	0.348485	25.17984	5.22E-07	0.009936
**RP11-343C2.7**	0.067797	0.257576	24.37647	7.92E-07	0.015074
**SPAG1**	0.09322	0.393939	45.79801	1.31E-11	2.49E-07
**DPY19L4**	0.088983	0.371212	41.96161	9.31E-11	1.77E-06
**MVD**	0.122881	0.348485	25.17984	5.22E-07	0.009936
**ARC**	0.088983	0.363636	40.13285	2.37E-10	4.51E-06
**SNTG1**	0.084746	0.310606	29.61614	5.27E-08	0.001002
**SLC10A5**	0.088983	0.356061	38.3313	5.97E-10	1.14E-05
**SLC7A6OS**	0.059322	0.242424	24.30112	8.24E-07	0.015676
**DPYS**	0.09322	0.409091	49.63179	1.85E-12	3.53E-08
**CAPNS2**	0.050847	0.227273	24.34508	8.05E-07	0.015322
**RSPRY1**	0.050847	0.242424	27.72176	1.40E-07	0.002665
**PTP4A3**	0.088983	0.356061	38.3313	5.97E-10	1.14E-05
**AC108925.1**	0.088983	0.401515	49.54176	1.94E-12	3.69E-08
**TIGD5**	0.088983	0.356061	38.3313	5.97E-10	1.14E-05
**MON1B**	0.127119	0.356061	25.45059	4.54E-07	0.008635
**CTC-479C5.12**	0.055085	0.242424	25.96278	3.48E-07	0.006622
**CDH11**	0.059322	0.25	25.94183	3.52E-07	0.006695
**CDH8**	0.055085	0.265152	31.10892	2.44E-08	0.000464
**NCOA2**	0.084746	0.363636	41.87726	9.72E-11	1.85E-06
**AC026248.1**	0.088983	0.393939	47.60756	5.21E-12	9.91E-08
**POP1**	0.088983	0.393939	47.60756	5.21E-12	9.91E-08
**NUDT21**	0.050847	0.25	29.4537	5.73E-08	0.00109
**CA1**	0.09322	0.356061	36.68223	1.39E-09	2.65E-05
**LY6D**	0.088983	0.363636	40.13285	2.37E-10	4.51E-06
**AC090154.1**	0.080508	0.340909	38.16239	6.51E-10	1.24E-05
**CIAPIN1**	0.055085	0.242424	25.96278	3.48E-07	0.006622
**GLI4**	0.088983	0.363636	40.13285	2.37E-10	4.51E-06
**IL7**	0.09322	0.348485	34.94219	3.40E-09	6.46E-05
**RLTPR**	0.055085	0.234848	24.30645	8.22E-07	0.015632
**NPBWR1**	0.067797	0.310606	36.29438	1.70E-09	3.23E-05
**NETO2**	0.029661	0.181818	23.47133	1.27E-06	0.024123
**SLC25A32**	0.09322	0.393939	45.79801	1.31E-11	2.49E-07
**CA2**	0.088983	0.356061	38.3313	5.97E-10	1.14E-05
**SQLE**	0.088983	0.393939	47.60756	5.21E-12	9.91E-08
**CYC1**	0.088983	0.356061	38.3313	5.97E-10	1.14E-05
**PDP2**	0.059322	0.234848	22.69198	1.90E-06	0.03618
**PRKDC**	0.076271	0.287879	27.76286	1.37E-07	0.002609
**KCTD19**	0.059322	0.234848	22.69198	1.90E-06	0.03618
**TSPYL5**	0.088983	0.393939	47.60756	5.21E-12	9.91E-08
**NOL3**	0.055085	0.242424	25.96278	3.48E-07	0.006622
**ZFHX3**	0.127119	0.371212	28.48687	9.43E-08	0.001795
**MCMDC2**	0.084746	0.340909	36.45437	1.56E-09	2.97E-05
**FHOD1**	0.055085	0.242424	25.96278	3.48E-07	0.006622

TRAM1	0.084746	0.348485	38.23476	6.27E-10	1.19E-05
IST1	0.101695	0.30303	22.50389	2.10E-06	0.039901
SLC30A8	0.101695	0.393939	42.35407	7.62E-11	1.45E-06
ADGRB1	0.088983	0.363636	40.13285	2.37E-10	4.51E-06
GRHL2	0.09322	0.393939	45.79801	1.31E-11	2.49E-07
NUP93	0.050847	0.242424	27.72176	1.40E-07	0.002665
CPA6	0.09322	0.340909	33.23083	8.18E-09	0.000156
EXOC3L1	0.055085	0.242424	25.96278	3.48E-07	0.006622
UTP23	0.101695	0.386364	40.53896	1.93E-10	3.67E-06
GPR20	0.088983	0.356061	38.3313	5.97E-10	1.14E-05
RIPK2	0.097458	0.363636	36.8277	1.29E-09	2.46E-05
RP11-5A19.5	0.055085	0.234848	24.30645	8.22E-07	0.015632
CNTNAP4	0.127119	0.363636	26.95268	2.08E-07	0.003967
ANGPT1	0.088983	0.401515	49.54176	1.94E-12	3.69E-08
RP11-520P18.5	0.088983	0.363636	40.13285	2.37E-10	4.51E-06
FAM150A	0.063559	0.325758	41.88063	9.70E-11	1.85E-06
C8orf89	0.084746	0.348485	38.23476	6.27E-10	1.19E-05
CTCF	0.055085	0.234848	24.30645	8.22E-07	0.015632
MSC	0.080508	0.356061	41.81861	1.00E-10	1.91E-06
NBN	0.09322	0.363636	38.4505	5.62E-10	1.07E-05
LY6H	0.088983	0.363636	40.13285	2.37E-10	4.51E-06
CDT1	0.122881	0.348485	25.17984	5.22E-07	0.009936
RP1-4G17.5	0.101695	0.30303	22.50389	2.10E-06	0.039901
CHD3	0.105932	0.310606	22.72338	1.87E-06	0.035593
TERF2	0.072034	0.257576	22.88168	1.72E-06	0.032779
MT4	0.050847	0.242424	27.72176	1.40E-07	0.002665
CYP11B1	0.088983	0.363636	40.13285	2.37E-10	4.51E-06
ENKD1	0.055085	0.234848	24.30645	8.22E-07	0.015632
TBC1D31	0.09322	0.378788	42.07009	8.81E-11	1.68E-06
SLC12A3	0.050847	0.242424	27.72176	1.40E-07	0.002665
POLR2K	0.09322	0.393939	45.79801	1.31E-11	2.49E-07
TGS1	0.067797	0.30303	34.5066	4.25E-09	8.08E-05
ZNF706	0.09322	0.401515	47.7018	4.96E-12	9.44E-08
DECR1	0.09322	0.371212	40.24658	2.24E-10	4.26E-06
USB1	0.050847	0.242424	27.72176	1.40E-07	0.002665
GRINA	0.088983	0.363636	40.13285	2.37E-10	4.51E-06
MYOM2	0.207627	0.454545	23.58762	1.19E-06	0.022708
SYBU	0.101695	0.393939	42.35407	7.62E-11	1.45E-06
CA7	0.059322	0.234848	22.69198	1.90E-06	0.03618
SLC52A1	0.042373	0.204545	22.85774	1.74E-06	0.03319
TRPA1	0.084746	0.356061	40.04253	2.48E-10	4.73E-06
TMEM67	0.09322	0.371212	40.24658	2.24E-10	4.26E-06
MMP16	0.09322	0.371212	40.24658	2.24E-10	4.26E-06
GABARAPL2	0.118644	0.356061	27.96047	1.24E-07	0.002356

**CCL17**	0.055085	0.242424	25.96278	3.48E-07	0.006622
**PI15**	0.088983	0.363636	40.13285	2.37E-10	4.51E-06
**OXR1**	0.101695	0.401515	44.19669	2.97E-11	5.65E-07
**LACTB2**	0.080508	0.348485	39.97716	2.57E-10	4.89E-06
**MIS12**	0.050847	0.219697	22.70249	1.89E-06	0.035982
**TMEM231**	0.118644	0.356061	27.96047	1.24E-07	0.002356
**KIAA0895L**	0.055085	0.242424	25.96278	3.48E-07	0.006622
**MMP15**	0.050847	0.242424	27.72176	1.40E-07	0.002665
**C1QBP**	0.050847	0.219697	22.70249	1.89E-06	0.035982
**ALOXE3**	0.067797	0.265152	25.99009	3.43E-07	0.006529
**NDEL1**	0.055085	0.227273	22.68139	1.91E-06	0.03638
**COL14A1**	0.097458	0.378788	40.38235	2.09E-10	3.97E-06
**FABP9**	0.088983	0.363636	40.13285	2.37E-10	4.51E-06
**AC092384.1**	0.122881	0.348485	25.17984	5.22E-07	0.009936
**ADHFE1**	0.084746	0.340909	36.45437	1.56E-09	2.97E-05
**HPR**	0.101695	0.318182	25.50461	4.41E-07	0.008397
**PABPC1**	0.09322	0.401515	47.7018	4.96E-12	9.44E-08
**CES5A**	0.050847	0.242424	27.72176	1.40E-07	0.002665
**SNX16**	0.088983	0.356061	38.3313	5.97E-10	1.14E-05
