# Supplementary material for: An Integrative Multi-Omics Analysis Based on Nomogram for Predicting Prostate Cancer Bone Metastasis Incidence
Source: Genet Res (Camb). 2022 Sep 29;2022:8213723. doi: 10.1155/2022/8213723 (PMC9537037; doi:10.1155/2022/8213723)
Supplement: Supplementary Materials — Figure S1: associated with Figure 2. Figure S2: associated with Figure 5. Figure S3: associated with Figure 6. Data 1-1: SEER data baseline characteristics of patient with prostate cancer; Data 1-2: train group ROC and validation ROC, Delong's test; Data 1-3: clinical features of TCGA samples and predictive results. Data 2: TMB scores of TCGA samples. Data 3: genes with different frequencies of CNVs between LRBM and HRBM cohorts. Data 4-1: DElncRNAs; Data 4-2: DEmiRNAs; Data 4-3: DEmRNAs. Data 5: gene module. Data 6-1: 29 immune microenvironment signatures; Data-2: ssGSEA score of immune microenvironment signature; Data 6-3: stromal score, immune score, estimate score, and tumor purity of TCGA samples. [file 8213723.f1.zip › 8213723.f1/supplementary data4 (1).pdf]

Supplementary Data4-1:DElncRNAs					
	**gene**	**mean of LRBM group**	**mean of HRBM group**	**log2FoldChange**	**padj**
	AC018816.1|ENSG00000235978|antisense	34.75661	202.1529	2.540243	6.66E-30
	AC023301.1|ENSG00000265579|sense_intronic	6.909692	83.73592	3.082113	4.78E-19
	BHLHE40-AS1|ENSG00000235831|antisense	57.72646	160.2995	1.040438	4.76E-14
	AC245014.3|ENSG00000276216|lincRNA	10.27128	21.59696	1.067938	1.58E-13
	LINC01141|ENSG00000236963|lincRNA	2.117838	7.083595	1.718817	1.79E-13
	AC093904.4|ENSG00000261104|lincRNA	3.71679	15.53514	2.051828	8.80E-13
	AC084346.2|ENSG00000264448|lincRNA	3.390334	8.371495	1.303698	1.81E-12
	AC022098.3|ENSG00000267783|antisense	3.246762	7.251891	1.176909	5.31E-12
	NAALADL2-AS2|ENSG00000226779|antisense	9.918371	31.54555	2.09173	1.38E-11
	AC002456.1|ENSG00000223969|antisense	27.87736	57.18676	1.032316	1.38E-11
	AC104076.1|ENSG00000236153|antisense	3.291124	9.124494	1.476659	3.12E-11
	AC093001.1|ENSG00000244468|antisense	572.3692	529.9721	1.640703	1.10E-10
	AC117382.2|ENSG00000261758|antisense	3.25067	11.03319	1.218317	2.37E-10
	AC010186.1|ENSG00000256442|antisense	0.944529	2.987325	1.696759	2.62E-10
	AL353753.1|ENSG00000276759|lincRNA	8.824984	86.65824	3.57264	5.04E-10
	LINC01612|ENSG00000250266|lincRNA	5.270339	33.06912	2.650708	5.39E-10
	AC004233.4|ENSG00000274367|lincRNA	1.15924	2.946534	1.355113	5.46E-10
	AL109615.2|ENSG00000231881|lincRNA	0.456037	1.835784	1.925238	5.79E-10
	LINC02544|ENSG00000261039|lincRNA	1.660636	2.093742	1.592711	6.45E-10
	AL445490.1|ENSG00000225886|antisense	4.493652	13.41467	1.58039	6.83E-10

	TERC|ENSG00000270141|lincRNA	0.71504	2.446127	1.798436	1.09E-09
	BX284668.6|ENSG00000272426|lincRNA	3.293129	7.214394	1.136882	2.45E-09
	AC005041.3|ENSG00000272183|antisense	2.517535	5.443076	1.109396	3.20E-09
	AC098818.2|ENSG00000260278|antisense	1.233542	3.438975	1.505091	3.43E-09
	KRTAP5-AS1|ENSG00000233930|antisense	23.02781	46.13981	1.005365	4.63E-09
	NRIR|ENSG00000225964|antisense	4.546162	9.919155	1.134255	5.79E-09
	AC015849.5|ENSG00000270977|lincRNA	4.163002	9.096438	1.101872	7.12E-09
	LINC00482|ENSG00000185168|lincRNA	15.98309	36.82107	1.20551	1.19E-08
	AC017074.1|ENSG00000227359|lincRNA	36.3647	107.2414	1.06892	2.75E-08
	AL032819.1|ENSG00000260132|antisense	2.315696	5.208488	1.177126	2.75E-08
	LINC01614|ENSG00000230838|lincRNA	8.840563	8.555733	1.13946	2.84E-08
	AP001625.3|ENSG00000239930|sense_intronic	2.909893	7.547679	1.106394	2.89E-08
	AL645608.8|ENSG00000273443|lincRNA	2.042168	5.007897	1.29397	3.02E-08
	AC106028.5|ENSG00000275175|lincRNA	0.980378	2.748575	1.525764	3.02E-08
	LINC01833|ENSG00000259439|lincRNA	0.390021	1.698497	2.000266	3.26E-08
	AC091138.1|ENSG00000263958|lincRNA	0.525235	4.825675	2.575591	5.66E-08
	AC093904.2|ENSG00000242136|processed_transcript	1.027144	3.560857	1.777232	6.13E-08
	AP001476.1|ENSG00000224413|lincRNA	11.44329	28.20428	1.291792	9.21E-08
	AC004835.1|ENSG00000230392|lincRNA	0.446939	1.670109	1.863703	1.20E-07
	AC083843.1|ENSG00000254028|lincRNA	2.320131	4.792195	1.052736	1.22E-07
	AC125603.2|ENSG00000257883|lincRNA	2.562687	6.693219	1.374228	2.14E-07
	AL133163.3|ENSG00000259017|lincRNA	5.358478	14.05529	1.386963	2.40E-07

	AC105219.1|ENSG00000254548|antisense	2.458265	6.042513	1.319915	4.60E-07
	AL109615.3|ENSG00000237686|antisense	8.534421	17.14687	1.001996	4.95E-07
	LINC02512|ENSG00000251061|lincRNA	0.792019	7.431382	2.684898	6.13E-07
	AP005230.1|ENSG00000263745|lincRNA	1.747004	3.068884	1.304429	6.43E-07
	AC073316.1|ENSG00000217455|lincRNA	2.290347	8.103575	1.829307	6.43E-07
	AC093895.1|ENSG00000249001|antisense	1.235623	3.02226	2.26254	1.15E-06
	AC092535.3|ENSG00000251652|antisense	5.776653	12.04184	1.072123	1.17E-06
	AC009336.1|ENSG00000272729|antisense	1.700275	3.650186	1.106357	1.32E-06
	AC020907.3|ENSG00000269303|antisense	1.475215	2.924361	1.032764	1.76E-06
	AC008574.1|ENSG00000251293|lincRNA	0.322737	2.824159	2.223444	1.87E-06
	AL451065.1|ENSG00000223446|antisense	0.242986	3.029629	2.989464	2.33E-06
	AP006216.1|ENSG00000226645|antisense	0.862138	1.690322	1.020358	2.93E-06
	AL353803.4|ENSG00000261334|lincRNA	1.277491	6.298743	1.332763	3.14E-06
	FEZF1-AS1|ENSG00000230316|antisense	17.81723	36.82177	1.04677	4.50E-06
	AL391427.1|ENSG00000224251|antisense	1.064008	2.435334	1.176898	4.61E-06
	DSCR8|ENSG00000198054|lincRNA	0.493289	8.877932	2.95569	4.67E-06
	AP003472.1|ENSG00000253824|lincRNA	0.952918	2.248711	1.292831	9.22E-06
	KCNH1-IT1|ENSG00000234233|sense_intronic	0.656927	1.745627	1.407144	9.53E-06
	AC015813.3|ENSG00000266290|lincRNA	0.571443	1.837382	1.364229	1.37E-05
	LINC01497|ENSG00000237560|lincRNA	2.446534	5.043928	1.072253	1.81E-05
	AC010967.1|ENSG00000228033|lincRNA	1.134926	2.666425	1.236571	2.06E-05
	AC011352.3|ENSG00000251320|antisense	2.216404	5.299692	1.255581	2.18E-05

	AC011352.1|ENSG00000248362|antisense	1.356612	3.396567	1.324719	2.44E-05
	AC025575.2|ENSG00000258053|lincRNA	2.507681	5.410305	1.079021	2.54E-05
	LINC02100|ENSG00000248693|lincRNA	1.173963	2.579605	1.083735	3.08E-05
	AC010894.3|ENSG00000236449|antisense	8.801333	25.06289	1.294718	3.18E-05
	LINC02200|ENSG00000250358|lincRNA	1.323802	3.417358	1.356764	3.65E-05
	AC009478.1|ENSG00000225258|lincRNA	31.87299	68.73347	1.108774	3.85E-05
	AC078923.1|ENSG00000258077|lincRNA	0.508598	1.604277	1.691746	4.03E-05
	AC069228.1|ENSG00000258162|antisense	104.4757	316.2888	1.598722	4.73E-05
	AL391845.2|ENSG00000233542|antisense	1.697071	3.52261	1.063079	5.61E-05
	AC092894.1|ENSG00000243081|lincRNA	1.596991	12.04932	1.238907	6.28E-05
	AC061975.6|ENSG00000266830|lincRNA	1.206687	2.892653	1.230207	6.67E-05
	ELDR|ENSG00000280890|lincRNA	0.796079	1.669719	1.076253	8.03E-05
	AC061975.1|ENSG00000260777|lincRNA	0.935227	3.710201	1.006505	9.01E-05
	BARX1-DT|ENSG00000235601|lincRNA	0.318116	1.17068	1.742462	9.71E-05
	LINC01647|ENSG00000235643|lincRNA	0.567215	3.424641	1.60115	0.000102
	AC092598.1|ENSG00000223523|sense_overlapping	2.613606	8.20152	1.240417	0.000103
	SCHLAP1|ENSG00000281131|lincRNA	392.8384	981.1847	1.320598	0.000104
	LINC01551|ENSG00000186960|lincRNA	1.05174	4.193611	1.890539	0.000119
	AC110813.1|ENSG00000249690|antisense	1.644121	5.968172	1.055446	0.000156
	MIR2052HG|ENSG00000254349|lincRNA	58.80805	126.475	1.103155	0.000174
	AC026726.1|ENSG00000249483|lincRNA	0.914457	1.844853	1.045832	0.000243
	AC131902.1|ENSG00000253369|lincRNA	58.52327	104.6274	1.13092	0.000243

	AC116345.1|ENSG00000249743|lincRNA	1.765773	3.92809	1.15459	0.000276
	AC106772.2|ENSG00000250385|antisense	0.812825	2.595326	1.712578	0.000283
	LAMP5-AS1|ENSG00000225988|antisense	3.058746	7.562233	1.176344	0.000325
	LINC02201|ENSG00000251538|lincRNA	0.440446	1.139505	1.257308	0.000332
	AP002759.1|ENSG00000255178|lincRNA	0.328501	1.271815	1.849651	0.000365
	LINC02197|ENSG00000250387|lincRNA	8.9878	20.94546	1.218704	0.0004
	AL021395.1|ENSG00000233508|lincRNA	17.6817	40.69744	1.204973	0.000401
	AL121906.1|ENSG00000271803|antisense	0.482603	1.029283	1.114903	0.000415
	ZFPM2-AS1|ENSG00000251003|processed_transcript	1.506563	3.272954	1.05248	0.000503
	AC092920.1|ENSG00000232461|lincRNA	0.866729	1.892167	1.119301	0.000513
	AC010132.1|ENSG00000231539|lincRNA	0.767049	3.342699	2.164534	0.000537
	LINC00536|ENSG00000249917|lincRNA	0.469769	1.02928	1.11142	0.000578
	WASIR1|ENSG00000185203|antisense	1.675163	3.879355	1.198844	0.000613
	AC008109.1|ENSG00000266602|lincRNA	0.44807	1.272422	1.899328	0.000731
	KCNMB2-AS1|ENSG00000237978|antisense	16.92109	36.31916	1.097431	0.000808
	AC087379.2|ENSG00000254695|lincRNA	1.277437	6.662298	1.797145	0.000848
	AL596442.1|ENSG00000232197|antisense	1.00686	3.291548	1.697834	0.000871
	AC093673.2|ENSG00000272619|lincRNA	0.466353	1.749009	1.303243	0.000945
	AC009139.1|ENSG00000260922|antisense	0.542523	1.283472	1.280843	0.001005
	AC141424.1|ENSG00000241525|antisense	0.658086	1.302336	1.001179	0.001014
	AC008897.2|ENSG00000247372|antisense	0.581043	1.16863	1.024299	0.001099
	LINC01446|ENSG00000205628|lincRNA	3.168834	8.163633	1.694825	0.001119

	AC092131.1|ENSG00000259846|lincRNA	0.713509	1.512516	1.039322	0.001149
	AC017074.2|ENSG00000233479|lincRNA	0.319972	1.46787	2.062344	0.001153
	AC024559.2|ENSG00000276407|lincRNA	0.304632	0.762996	1.193162	0.00134
	AL022725.1|ENSG00000236336|antisense	0.34793	0.986533	1.362257	0.001345
	AC104564.4|ENSG00000264647|antisense	1.135951	2.345188	1.033182	0.001379
	AL591468.1|ENSG00000226004|lincRNA	0.523508	1.066902	1.060856	0.001622
	AL136537.2|ENSG00000261761|processed_transcript	1.040884	10.54705	2.406984	0.001669
	AL442224.1|ENSG00000231990|lincRNA	0.641586	1.51954	1.250652	0.001694
	AC040914.1|ENSG00000254372|antisense	0.451864	0.899099	1.03704	0.00173
	AC069277.1|ENSG00000189229|lincRNA	2.080556	5.308855	1.541657	0.00176
	PPFIA2-AS1|ENSG00000257467|lincRNA	14.1842	29.82897	1.07165	0.001786
	AC104350.1|ENSG00000253523|lincRNA	0.472803	1.035138	1.092637	0.002055
	GS1-594A7.3|ENSG00000225833|antisense	1.436066	2.268685	1.208306	0.002243
	AC020934.2|ENSG00000267735|antisense	0.340846	0.732796	1.033303	0.002358
	AC009908.1|ENSG00000253513|antisense	0.30401	0.744667	1.156941	0.002722
	CR392039.3|ENSG00000279501|lincRNA	0.439549	1.9246	1.692613	0.003179
	AC024132.3|ENSG00000251325|lincRNA	0.496985	1.682231	1.267552	0.003263
	AC092435.1|ENSG00000248346|lincRNA	0.24377	0.733256	1.282875	0.003485
	LINC02398|ENSG00000256287|lincRNA	0.822195	1.69705	1.070444	0.0042
	AC005786.2|ENSG00000267231|lincRNA	0.378605	0.772805	1.092034	0.004413
	AC078955.1|ENSG00000258026|antisense	0.500856	1.091392	1.068314	0.004445
	AC008554.1|ENSG00000269043|lincRNA	0.509949	1.109462	1.004373	0.004467

	AL596325.1|ENSG00000259788|antisense	0.309635	1.185439	1.222954	0.004952
	AC106712.1|ENSG00000241280|lincRNA	0.591426	1.276926	1.107401	0.005022
	AC022392.1|ENSG00000272988|lincRNA	0.537609	0.738664	1.150199	0.00517
	AC083801.2|ENSG00000260377|lincRNA	0.27766	0.684298	1.153363	0.006059
	AC099509.1|ENSG00000249746|lincRNA	0.451643	1.512606	1.281816	0.00644
	MIR137HG|ENSG00000225206|lincRNA	5.421167	39.04508	1.655922	0.007169
	DSCR4|ENSG00000184029|lincRNA	0.116279	1.136475	2.052426	0.008638
	AC007128.2|ENSG00000272328|lincRNA	1.384507	3.652707	1.383195	0.009048
	AL157778.1|ENSG00000281566|lincRNA	0.291009	1.501526	2.113233	0.009208
	AP000943.1|ENSG00000233536|lincRNA	0.675868	1.209258	1.184365	0.009215
	Z97206.2|ENSG00000237567|lincRNA	49.73662	74.94323	1.153517	0.010136
	AL122019.1|ENSG00000227579|lincRNA	0.724226	1.565383	1.099784	0.01045
	LINC02466|ENSG00000246876|lincRNA	0.904295	2.582322	1.49998	0.010575
	AC103843.1|ENSG00000255101|antisense	0.502981	1.089753	1.050555	0.01098
	AL157387.1|ENSG00000234918|lincRNA	11.51404	33.32962	1.684542	0.011706
	LINC02163|ENSG00000251026|lincRNA	3.818454	11.05466	1.533875	0.011825
	AC078820.1|ENSG00000258088|lincRNA	0.247192	0.755281	1.29673	0.012362
	AC112493.1|ENSG00000242021|lincRNA	1.625251	3.665025	1.15229	0.014532
	AC096734.1|ENSG00000251517|lincRNA	0.315943	1.008444	1.125227	0.014914
	AC107419.1|ENSG00000228933|lincRNA	5.122481	10.94769	1.088643	0.016186
	AP000769.3|ENSG00000279243|lincRNA	0.453668	0.984463	1.066421	0.017935
	TLX1NB|ENSG00000236311|lincRNA	0.24648	0.720146	1.167428	0.018301

	AL117382.1|ENSG00000226812|antisense	0.219496	0.620734	1.200498	0.018844
	AC010595.1|ENSG00000250874|lincRNA	0.248807	1.076083	1.734134	0.020617
	LINC01214|ENSG00000243550|lincRNA	0.328079	0.765471	1.06404	0.020774
	BX005019.1|ENSG00000259946|lincRNA	0.289252	1.361166	1.666793	0.020925
	AP003550.1|ENSG00000254236|antisense	0.128616	0.603728	1.455329	0.020972
	AC019103.1|ENSG00000250259|lincRNA	0.18692	0.738366	1.55238	0.024481
	Z82214.2|ENSG00000236272|antisense	0.247979	0.72868	1.275493	0.025127
	AP005121.1|ENSG00000263618|lincRNA	0.929192	1.159012	1.495331	0.026032
	AC128709.2|ENSG00000235126|lincRNA	0.154838	0.532751	1.196756	0.026599
	AC098934.4|ENSG00000260021|antisense	0.196401	0.813491	1.17912	0.034018
	LINC01632|ENSG00000277199|lincRNA	0.436626	1.583871	1.365521	0.0397
	AC104831.1|ENSG00000242790|lincRNA	1.221095	4.345699	1.069961	0.046468
	AC103808.6|ENSG00000266743|lincRNA	0.510027	1.193004	1.150721	0.04682
	AL354863.1|ENSG00000238276|processed_transcript	7.71649	12.86649	1.236258	0.046957
	AC020571.1|ENSG00000229056|antisense	1109.242	527.3623	-1.07286	1.79E-13
	AC010327.3|ENSG00000267577|antisense	25.59962	9.586386	-2.62403	1.47E-10
	RMDN2-AS1|ENSG00000235848|antisense	22.42528	9.515616	-1.23304	2.62E-10
	AL031663.3|ENSG00000277022|lincRNA	18.45962	7.749175	-1.24231	4.97E-10
	AGBL1-AS1|ENSG00000260125|antisense	6.906444	5.69764	-2.10579	5.25E-08
	FP671120.3|ENSG00000280800|lincRNA	16.10485	0.387636	-2.8461	2.34E-07
	AC147651.1|ENSG00000229380|antisense	6.029871	2.557739	-1.22474	3.70E-07
	LINC01698|ENSG00000231648|lincRNA	13.32582	10.09252	-1.5341	4.98E-07

	AC026469.1|ENSG00000275088|lincRNA	10.95408	4.1922	-1.07421	1.87E-06
	AL354714.1|ENSG00000198358|antisense	4.872881	3.17182	-1.01611	4.84E-06
	AC104137.1|ENSG00000248479|lincRNA	11.13705	5.355268	-1.05506	4.87E-06
	LINC02055|ENSG00000254101|lincRNA	6.569701	3.053603	-1.86353	1.06E-05
	AC116025.1|ENSG00000261978|antisense	6.4199	3.113259	-1.05774	1.77E-05
	AC007750.1|ENSG00000236841|antisense	148.6496	64.71382	-1.19953	1.96E-05
	AP002478.2|ENSG00000266513|sense_intronic	1.279814	0.440984	-1.55208	2.64E-05
	LINC02120|ENSG00000248279|lincRNA	22.40922	6.704046	-1.41957	2.94E-05
	AC018358.1|ENSG00000281160|antisense	3.597481	1.580827	-1.17971	4.45E-05
	AP001269.2|ENSG00000267079|lincRNA	1.861281	0.84653	-1.1158	5.03E-05
	AL449403.2|ENSG00000275465|lincRNA	14.42162	6.521651	-1.14293	6.57E-05
	AC113192.1|ENSG00000254619|lincRNA	1.306743	0.419279	-1.70828	7.45E-05
	AC246817.2|ENSG00000254319|lincRNA	28.07094	19.26867	-1.13376	9.71E-05
	AP001094.3|ENSG00000266149|antisense	20.43386	10.21921	-1.00176	0.000124
	AC084816.1|ENSG00000256995|lincRNA	1.412549	0.551978	-1.32521	0.000176
	AL138881.1|ENSG00000228793|lincRNA	36.16108	17.20624	-1.16974	0.000271
	LINC01187|ENSG00000249601|lincRNA	10.37152	6.845305	-1.28066	0.000296
	C17orf77|ENSG00000182352|antisense	9.561159	6.567937	-1.44085	0.000415
	AC113192.4|ENSG00000255169|lincRNA	2.665437	1.087269	-1.31341	0.000452
	LINC01139|ENSG00000215808|lincRNA	23.78963	11.11883	-1.09643	0.000494
	U95743.1|ENSG00000262267|lincRNA	5.207668	2.196374	-1.23131	0.000597
	LINC02064|ENSG00000248391|lincRNA	1.896448	0.777163	-1.25063	0.000599

	LINC01344|ENSG00000228918|lincRNA	2.124751	1.036982	-1.0448	0.000685
	LINC02199|ENSG00000250198|lincRNA	0.977953	0.708383	-1.56998	0.00089
	LINC00336|ENSG00000197251|lincRNA	1.150679	0.55006	-1.08443	0.001159
	AL158066.1|ENSG00000217576|processed_transcript	1.2502	0.59452	-1.2113	0.001177
	LINC01343|ENSG00000237290|lincRNA	0.766622	0.344418	-1.10595	0.001531
	AC093496.1|ENSG00000255021|lincRNA	30.45638	3.704583	-1.64847	0.002421
	C5orf17|ENSG00000248874|lincRNA	38.54037	19.63957	-1.2739	0.002613
	MIR1-1HG|ENSG00000174407|antisense	23.48873	8.743009	-1.00227	0.002839
	AC097713.1|ENSG00000228162|lincRNA	1.597669	0.731093	-1.11449	0.00369
	AC015878.1|ENSG00000265751|antisense	4.392856	1.531044	-2.38225	0.004412
	LINC01661|ENSG00000237460|lincRNA	0.79767	0.387757	-1.03229	0.006121
	AC121342.1|ENSG00000233403|lincRNA	4.164669	1.640698	-1.12918	0.006589
	PCAT5|ENSG00000280719|lincRNA	41.3794	19.20383	-1.10597	0.007802
	AC098936.1|ENSG00000224717|lincRNA	0.754084	0.32494	-1.00337	0.009208
	AL356277.3|ENSG00000272243|lincRNA	6.642004	2.712351	-1.29295	0.01192
	AC009142.1|ENSG00000261410|antisense	1.216266	0.550181	-1.06947	0.014504
	AL157414.1|ENSG00000203266|lincRNA	0.863688	0.404731	-1.03572	0.016186
	LINC02270|ENSG00000251210|lincRNA	0.648485	0.175739	-1.24702	0.023368
	AL161431.1|ENSG00000275216|lincRNA	1.784306	0.590855	-1.14493	0.02393
	LINC01581|ENSG00000259724|lincRNA	1.491329	0.611203	-1.08724	0.027237
	AL355596.1|ENSG00000259828|lincRNA	1.827645	1.312202	-1.02781	0.032676
	LINC01405|ENSG00000185847|lincRNA	29.28531	12.18328	-1.35348	0.038296

	AL391704.1|ENSG00000224750|lincRNA	1.387418	1.358059	-1.02264	0.04607

**Supplementary Data4-2:DEmiRNAs**					
	**gene**	**mean of LRBM group**	**mean of HRBM group**	**log2FoldChange**	**padj**
	hsa-mir-210	261.2413	689.144	1.399289	8.62E-17
	hsa-mir-592	5.584297	22.13319	1.331864	8.83E-11
	hsa-mir-653	7.418493	11.73756	0.664891	7.88E-10
	hsa-mir-5680	2.816552	5.569482	0.991199	7.38E-09
	hsa-mir-137	7.194244	25.86411	2.121851	1.63E-08
	hsa-mir-3614	2.251066	3.466262	0.63135	0.000459
	hsa-mir-7156	0.696134	1.472423	1.090436	0.000578
	hsa-mir-4664	0.509845	0.95362	0.930596	0.002506
	hsa-mir-767	1.240424	2.226141	1.269525	0.004606
	hsa-mir-449a	5.466919	9.52474	1.162333	0.005975
	hsa-mir-4501	0.844114	1.382606	0.769029	0.017915
	hsa-mir-612	0.91548	1.189156	1.075234	0.020103
	hsa-mir-4636	0.394895	0.649005	0.591864	0.035013
	hsa-mir-105-1	0.786305	2.100403	0.928557	0.03736
	hsa-mir-3662	0.29997	0.628479	0.686214	0.04795
	hsa-mir-5002	0.350238	0.586783	0.642352	0.049071
	hsa-mir-4766	0.469455	0.769493	0.629902	0.049951
	hsa-mir-184	9.460701	6.212005	-1.48424	5.94E-09
	hsa-mir-133b	97.28803	54.896	-0.82524	3.68E-08
	hsa-mir-133a-1	312.1982	194.4605	-0.68289	6.91E-08
	hsa-mir-133a-2	280.4872	178.0972	-0.65511	2.36E-07
	hsa-mir-508	179.5153	9.037174	-0.98947	2.10E-06
	hsa-mir-6510	26.67336	13.66561	-0.96154	2.74E-06
	hsa-mir-23c	2.928975	1.335848	-1.12683	5.76E-06
	hsa-mir-7641-	1.276956	0.367302	-1.75107	2.19E-05

	1				
	hsa-mir-6715b	8.748858	3.686533	-1.0408	6.84E-05
	hsa-mir-891a	20.74194	12.63247	-0.71985	6.84E-05
	hsa-mir-7641-2	0.88773	0.285565	-1.44272	0.000534
	hsa-mir-514a-1	13.38529	1.025652	-1.09355	0.000747
	hsa-mir-1258	5.779094	3.686174	-0.61435	0.001214
	hsa-mir-378d-1	1.165537	0.749523	-0.67395	0.003631
	hsa-mir-509-1	9.281989	0.698164	-0.92454	0.008238
	hsa-mir-3622a	1.003318	0.635903	-0.6416	0.010377
	hsa-mir-514a-3	13.50021	1.226487	-0.76929	0.01278
	hsa-mir-514a-2	13.68334	1.358583	-0.74592	0.013026
	hsa-mir-499a	3.163858	2.701792	-0.69831	0.013241
	hsa-mir-509-3	11.26123	0.764107	-0.86811	0.015366
	hsa-mir-509-2	9.179771	0.737556	-0.88285	0.019274

**Supplementary Data4-3:DEmRNAs**					
	**gene**	**mean of LRBM group**	**mean of HRBM group**	**log2FoldChange**	**padj**
	NETO1|ENSG00000166342|protein_coding	27.15553362	304.4266401	3.39337	6.71E-30
	UBE2C|ENSG00000175063|protein_coding	170.1506608	510.1268994	1.584716	6.11E-29
	TICRR|ENSG00000140534|protein_coding	68.78937596	138.2248842	1.004883	4.43E-27
	MYBL2|ENSG00000101057|protein_coding	248.2171393	667.9170427	1.427815	2.84E-26
	CENPA|ENSG00000115163|protein_coding	47.49012856	123.0165576	1.372467	2.91E-25
	ORC6|ENSG00000091651|protein_coding	78.8260329	158.0682171	1.004785	2.91E-25
	KIF18B|ENSG00000186185|protein_codi	61.886546	157.359111	1.344587	4.21E-

	ng	54	6		25
	RAD54L|ENSG00000085999|protein_coding	53.54047146	117.0030017	1.125885	1.68E-24
	CDC20|ENSG00000117399|protein_coding	209.9495882	489.7098139	1.221758	5.55E-24
	SHCBP1|ENSG00000171241|protein_coding	71.23556821	158.6929225	1.154959	7.65E-24
	PKMYT1|ENSG00000127564|protein_coding	153.5820711	318.9604388	1.054183	1.37E-23
	PLK1|ENSG00000166851|protein_coding	180.9160403	376.5091575	1.056918	2.79E-23
	TPX2|ENSG00000088325|protein_coding	376.0229466	832.8294228	1.146879	2.79E-23
	TROAP|ENSG00000135451|protein_coding	110.8512914	252.7883854	1.189564	7.19E-23
	UTS2B|ENSG00000188958|protein_coding	28.21694414	141.9021165	2.095441	1.07E-22
	POLQ|ENSG00000051341|protein_coding	49.23379832	112.0158303	1.182658	2.07E-22
	NUF2|ENSG00000143228|protein_coding	63.45344636	135.4787743	1.093786	2.32E-22
	PTPRR|ENSG00000153233|protein_coding	42.66492278	351.2890524	2.005415	4.94E-22
	MCM10|ENSG00000065328|protein_coding	44.08995242	102.7311374	1.216905	5.03E-22
	CDKN3|ENSG00000100526|protein_coding	83.33538227	172.2731209	1.046928	7.23E-22
	EXO1|ENSG00000174371|protein_coding	56.18046798	124.0383575	1.13859	1.06E-21
	SUCNR1|ENSG00000198829|protein_coding	51.80892836	430.8736002	2.05773	2.73E-21
	KIF15|ENSG00000163808|protein_coding	102.3524725	213.9613303	1.062386	4.86E-21
	MELK|ENSG00000165304|protein_coding	111.9288611	249.2078146	1.153372	4.86E-21
	INSM2|ENSG00000168348|protein_coding	2.235753544	21.98545493	2.783232	5.21E-21
	CDK1|ENSG00000170312|protein_coding	317.2413161	684.5634118	1.10938	6.08E-21
	KIF4A|ENSG00000090889|protein_coding	171.0679592	388.1306993	1.181324	6.08E-21
	UHRF1|ENSG00000276043|protein_coding	120.3950211	256.7186579	1.090804	1.03E-20

	ESPL1|ENSG00000135476|protein_coding	99.71199492	211.851972	1.085404	1.45E-20
	NEK2|ENSG00000117650|protein_coding	84.39167807	198.6083704	1.233607	1.47E-20
	BIRC5|ENSG00000089685|protein_coding	291.1705751	640.8928543	1.138329	3.10E-20
	ASF1B|ENSG00000105011|protein_coding	200.4636169	408.0338171	1.02457	3.40E-20
	FAM72B|ENSG00000188610|protein_coding	9.585061807	20.4172371	1.075957	3.40E-20
	SGO1|ENSG00000129810|protein_coding	36.13915244	80.21942772	1.148073	6.03E-20
	DLGAP5|ENSG00000126787|protein_coding	98.10358003	216.9948557	1.144025	1.19E-19
	EFNA3|ENSG00000143590|protein_coding	167.5430742	348.9844078	1.059713	1.74E-19
	BUB1|ENSG00000169679|protein_coding	161.4451886	341.6848051	1.080451	1.86E-19
	COL11A1|ENSG00000060718|protein_coding	100.6922355	133.2059591	1.891483	2.39E-19
	IQGAP3|ENSG00000183856|protein_coding	165.9229413	351.9630384	1.084429	2.72E-19
	CBX2|ENSG00000173894|protein_coding	212.0243516	462.4560852	1.125108	2.81E-19
	RRM2|ENSG00000171848|protein_coding	354.756884	776.2249808	1.129205	3.05E-19
	SYT4|ENSG00000132872|protein_coding	116.4260089	517.5414346	2.44689	4.95E-19
	NUSAP1|ENSG00000137804|protein_coding	325.3788802	660.874498	1.02168	6.29E-19
	MTFR2|ENSG00000146410|protein_coding	21.37343419	43.09334906	1.00562	1.37E-18
	NCAPG|ENSG00000109805|protein_coding	112.6870927	237.0773391	1.071995	3.04E-18
	KIF20A|ENSG00000112984|protein_coding	165.4435001	359.3774421	1.118746	3.72E-18
	CKAP2L|ENSG00000169607|protein_coding	62.59353613	136.3941355	1.121124	4.29E-18
	PNMA5|ENSG00000198883|protein_coding	2.376691582	53.95010454	2.38516	5.21E-18
	LIPF|ENSG00000182333|protein_coding	2.934990394	1565.715082	5.037251	8.23E-18
	HOXB5|ENSG00000120075|protein_coding	17.50194917	31.02549784	1.603758	1.06E-17

	ANLN|ENSG00000011426|protein_coding	193.2785863	408.949242	1.080329	1.18E-17
	NCAPH|ENSG00000121152|protein_coding	111.0796271	224.811448	1.01588	2.10E-17
	CENPF|ENSG00000117724|protein_coding	428.6132485	890.0473507	1.05383	2.35E-17
	HOXD1|ENSG00000128645|protein_coding	19.90912907	69.38620597	1.980592	2.58E-17
	CEP55|ENSG00000138180|protein_coding	105.8285185	223.1587978	1.073763	3.35E-17
	TOP2A|ENSG00000131747|protein_coding	699.8175403	1526.760724	1.125223	3.93E-17
	NEIL3|ENSG00000109674|protein_coding	19.33802505	43.55579859	1.165385	9.26E-17
	SERPINI1|ENSG00000163536|protein_coding	489.2070087	1167.849835	1.25535	9.84E-17
	NCAM2|ENSG00000154654|protein_coding	269.6563626	668.2987104	1.514659	1.21E-16
	GTSE1|ENSG00000075218|protein_coding	109.5203877	221.5761032	1.016109	1.53E-16
	CDC25C|ENSG00000158402|protein_coding	49.49097027	99.24381684	1.00406	2.07E-16
	KLK14|ENSG00000129437|protein_coding	160.3737285	462.4439725	1.781653	2.30E-16
	FAM72D|ENSG00000215784|protein_coding	4.519289203	10.21458859	1.192124	3.30E-16
	KIF14|ENSG00000118193|protein_coding	47.41849769	99.57402252	1.067599	1.06E-15
	THBS2|ENSG00000186340|protein_coding	1709.775493	2888.208559	1.025935	2.96E-15
	TUBB3|ENSG00000258947|protein_coding	35.84164571	82.21696207	1.19578	3.57E-15
	EFNA2|ENSG00000099617|protein_coding	7.280486342	23.41073109	1.690546	4.18E-15
	KRT75|ENSG00000170454|protein_coding	15.53551938	47.0820355	2.126786	4.58E-15
	ASPM|ENSG00000066279|protein_coding	126.7887162	264.406398	1.058856	6.22E-15
	HJURP|ENSG00000123485|protein_coding	178.1488793	367.1515987	1.043037	6.24E-15
	PBK|ENSG00000168078|protein_coding	80.28501012	169.0648404	1.073109	6.74E-15
	SGK2|ENSG00000101049|protein_coding	48.92088677	125.1915838	1.355807	9.77E-15

	DEPDC1B|ENSG00000035499|protein_coding	74.36828132	153.0511106	1.040034	6.44E-14
	SSTR1|ENSG00000139874|protein_coding	762.2074702	2026.157839	1.410517	1.22E-13
	CTHRC1|ENSG00000164932|protein_coding	278.6066037	442.723946	1.010921	1.97E-13
	RDM1|ENSG00000278023|protein_coding	10.22573368	21.29616862	1.061828	2.51E-13
	PLEKHD1|ENSG00000175985|protein_coding	19.78704153	41.1822533	1.061277	4.16E-13
	AKR1C3|ENSG00000196139|protein_coding	240.4155667	518.3709178	1.108767	6.93E-13
	BMP6|ENSG00000153162|protein_coding	370.8620688	783.3268508	1.078948	8.51E-13
	CAMKV|ENSG00000164076|protein_coding	4.745831218	30.2571365	1.886628	1.23E-12
	E2F8|ENSG00000129173|protein_coding	33.67394131	68.61767363	1.023113	1.43E-12
	DNAH8|ENSG00000124721|protein_coding	1475.247515	3433.710147	1.523547	3.57E-12
	UGT2B15|ENSG00000196620|protein_coding	52.45212123	131.2621319	1.803824	4.53E-12
	SCRT1|ENSG00000261678|protein_coding	3.841716967	23.98900963	1.197497	9.35E-12
	MYT1|ENSG00000196132|protein_coding	49.35823376	158.0544178	1.678535	1.16E-11
	MUC1|ENSG00000185499|protein_coding	1242.155985	2937.868383	1.241927	1.26E-11
	PTK6|ENSG00000101213|protein_coding	614.8531393	1295.920662	1.075653	3.00E-11
	EOMES|ENSG00000163508|protein_coding	43.19780976	96.39115045	1.155663	3.11E-11
	COL10A1|ENSG00000123500|protein_coding	453.9047021	759.116208	1.320095	3.81E-11
	IFI44L|ENSG00000137959|protein_coding	460.4996621	938.7982605	1.027903	4.64E-11
	CELSR3|ENSG00000008300|protein_coding	300.916533	618.2586197	1.039042	9.57E-11
	S100P|ENSG00000163993|protein_coding	726.9955498	1618.352579	1.520938	9.61E-11
	OMP|ENSG00000254550|protein_coding	4.67261063	11.15725314	1.248435	1.01E-10
	HTR2A|ENSG00000102468|protein_coding	11.72012019	25.88493452	1.143982	1.64E-10

	HOXB6|ENSG00000108511|protein_coding	34.40838945	56.6334882	1.018765	1.92E-10
	CST2|ENSG00000170369|protein_coding	718.3465453	1632.081965	1.470239	2.14E-10
	PDIA2|ENSG00000185615|protein_coding	96.35176931	288.2029444	1.581974	2.36E-10
	FNDC1|ENSG00000164694|protein_coding	107.7344583	153.5520622	1.118859	3.06E-10
	MAGEA8|ENSG00000156009|protein_coding	7.842683696	30.97650529	1.668845	3.06E-10
	FGB|ENSG00000171564|protein_coding	15.86237548	134.3820205	3.15592	3.18E-10
	LHX2|ENSG00000106689|protein_coding	5.669855772	13.5385162	1.267422	3.99E-10
	ZBBX|ENSG00000169064|protein_coding	12.98785696	36.21470582	1.798234	4.89E-10
	FAM72C|ENSG00000263513|protein_coding	2.817033615	5.910498521	1.094672	4.95E-10
	LRTM2|ENSG00000166159|protein_coding	4.3720224	15.33975593	1.421352	1.31E-09
	MAB21L2|ENSG00000181541|protein_coding	8.46093593	18.25121112	1.12601	1.36E-09
	ZPLD1|ENSG00000170044|protein_coding	1.322770979	3.530349158	1.449131	2.41E-09
	TTC29|ENSG00000137473|protein_coding	14.79242137	34.1944224	1.520848	2.51E-09
	CDC20B|ENSG00000164287|protein_coding	50.77245127	483.6451572	1.729127	3.77E-09
	A1CF|ENSG00000148584|protein_coding	18.86801044	48.70315339	1.639357	6.59E-09
	ODF3L2|ENSG00000181781|protein_coding	15.52562915	32.42101452	1.064747	6.92E-09
	DHRS2|ENSG00000100867|protein_coding	104.3925647	547.6246756	1.311062	6.96E-09
	C6orf10|ENSG00000204296|protein_coding	4.129058247	32.47222065	1.751499	7.25E-09
	HTR1F|ENSG00000179097|protein_coding	5.979435654	12.44271495	1.04891	8.03E-09
	BARX1|ENSG00000131668|protein_coding	10.45066846	26.57431845	1.350808	8.48E-09
	DIRAS2|ENSG00000165023|protein_coding	65.56205534	173.7028985	1.405485	9.70E-09
	RAB3C|ENSG00000152932|protein_coding	18.95989505	38.88793522	1.036568	2.31E-08

	SI|ENSG00000090402|protein_coding	354.2966557	929.5965287	1.391539	2.43E-08
	DQX1|ENSG00000144045|protein_coding	10.3007988	27.07765783	1.105081	3.05E-08
	PCDHGA5|ENSG00000253485|protein_coding	326.6614556	673.3254174	1.043436	3.19E-08
	ARHGDIG|ENSG00000242173|protein_coding	138.9181691	293.3847196	1.08093	3.58E-08
	DMBX1|ENSG00000197587|protein_coding	3.344948563	7.445152673	1.149339	5.17E-08
	GRIK1|ENSG00000171189|protein_coding	152.4237445	353.3016055	1.212819	6.01E-08
	ACY3|ENSG00000132744|protein_coding	57.40363795	440.3301713	1.286269	7.90E-08
	TUNAR|ENSG00000250366|protein_coding	38.46562351	111.0731132	1.770529	9.00E-08
	CA1|ENSG00000133742|protein_coding	4.582838903	10.22448419	1.749662	9.89E-08
	CCDC194|ENSG00000269720|protein_coding	0.995184105	5.986367478	1.528257	1.10E-07
	TEX19|ENSG00000182459|protein_coding	1.019521599	2.42676782	1.202358	1.67E-07
	SLC9A3|ENSG00000066230|protein_coding	100.4358579	209.2067083	1.057969	2.33E-07
	FAM135B|ENSG00000147724|protein_coding	40.50337111	99.16844219	1.292561	2.43E-07
	AKR1D1|ENSG00000122787|protein_coding	4.630258566	9.60348768	1.349796	3.05E-07
	HIST1H3B|ENSG00000274267|protein_coding	2.756117059	6.696680512	1.28093	3.18E-07
	IL36RN|ENSG00000136695|protein_coding	10.60717067	27.66429071	1.387746	3.18E-07
	WFDC5|ENSG00000175121|protein_coding	50.25068263	212.3805826	1.45405	3.94E-07
	HIST1H3C|ENSG00000278272|protein_coding	3.417190215	7.502142568	1.137322	4.04E-07
	CST5|ENSG00000170367|protein_coding	6.371963379	11.92059986	1.16521	4.46E-07
	BRS3|ENSG00000102239|protein_coding	0.492803493	1.522274437	1.717721	4.70E-07
	MATN4|ENSG00000124159|protein_coding	9.198508289	24.55234146	1.080706	6.49E-07
	FOXG1|ENSG00000176165|protein_coding	7.007541225	13.08637713	2.379006	6.85E-07

	MAGEA1|ENSG00000198681|protein_coding	1.816198245	35.99456507	3.461184	7.47E-07
	OPRK1|ENSG00000082556|protein_coding	419.1943196	842.9052192	1.007591	7.47E-07
	ASZ1|ENSG00000154438|protein_coding	2.353525008	19.54261083	1.621493	1.10E-06
	NKX6-1|ENSG00000163623|protein_coding	32.90444412	89.17425124	1.43761	1.38E-06
	ANO3|ENSG00000134343|protein_coding	32.82203637	36.35669198	1.100239	1.82E-06
	VWA5B1|ENSG00000158816|protein_coding	62.17154805	221.1171698	1.531688	1.82E-06
	C5orf46|ENSG00000178776|protein_coding	16.27791753	35.58417531	1.13049	1.83E-06
	CCDC144NL|ENSG00000205212|protein_coding	14.94152924	43.47109418	1.228983	2.48E-06
	RFX6|ENSG00000185002|protein_coding	82.945683	186.4445286	1.168548	2.87E-06
	TYRP1|ENSG00000107165|protein_coding	40.48628205	86.30692453	1.09255	4.82E-06
	DGKK|ENSG00000274588|protein_coding	7.058782018	28.95307244	1.081806	5.78E-06
	ZNF556|ENSG00000172000|protein_coding	34.40526279	86.06196258	1.321238	5.97E-06
	KCNQ2|ENSG00000075043|protein_coding	125.8608435	343.4536683	1.200541	6.52E-06
	AVPR1B|ENSG00000198049|protein_coding	3.086727596	11.08243159	1.101606	7.95E-06
	OTOR|ENSG00000125879|protein_coding	2.064396372	10.38930071	1.876689	1.72E-05
	HAND1|ENSG00000113196|protein_coding	0.945855137	3.215906139	1.771626	1.79E-05
	AP002373.1|ENSG00000255663|protein_coding	0.583488364	1.445583568	1.331538	1.89E-05
	TRPC5OS|ENSG00000204025|protein_coding	0.404611826	0.915342492	1.143546	1.92E-05
	MUC6|ENSG00000184956|protein_coding	901.9215107	1881.274976	1.583262	1.93E-05
	TUBA4B|ENSG00000243910|protein_coding	23.1771235	23.9616762	1.234232	2.01E-05
	CLRN3|ENSG00000180745|protein_coding	1.653029137	7.692290912	1.228037	2.04E-05
	ADAM18|ENSG00000168619|protein_coding	1.604581943	3.588764605	1.555583	2.15E-05

	CALML5|ENSG00000178372|protein_coding	3.94936597	143.3264271	1.176178	2.30E-05
	TM4SF20|ENSG00000168955|protein_coding	1.714362768	15.70553242	1.327753	2.38E-05
	SOX11|ENSG00000176887|protein_coding	7.82146957	13.51815258	1.141241	2.69E-05
	STMN2|ENSG00000104435|protein_coding	85.41023695	170.7980044	1.000067	3.10E-05
	CTAG2|ENSG00000126890|protein_coding	28.3641054	33.02625191	2.972305	3.12E-05
	ATP12A|ENSG00000075673|protein_coding	127.2561816	804.0908348	1.223573	3.19E-05
	SLC17A4|ENSG00000146039|protein_coding	29.70293551	71.76673499	1.611312	3.25E-05
	KCNK4|ENSG00000182450|protein_coding	0.445615278	1.054929458	1.219146	3.36E-05
	KHDC1L|ENSG00000256980|protein_coding	7.709907134	26.49770586	1.135921	3.36E-05
	PAEP|ENSG00000122133|protein_coding	2.152418403	5.717115147	1.52631	3.63E-05
	ASIC2|ENSG00000108684|protein_coding	55.77603816	123.5523609	1.147163	4.37E-05
	CER1|ENSG00000147869|protein_coding	0.887034189	1.672919602	1.356887	4.38E-05
	FCRL2|ENSG00000132704|protein_coding	18.83686457	25.44743377	1.014591	4.63E-05
	HIST1H2AJ|ENSG00000276368|protein_coding	0.967572939	1.991942237	1.085918	4.87E-05
	PPFIA2|ENSG00000139220|protein_coding	481.0711157	1029.000932	1.096883	6.28E-05
	IBSP|ENSG00000029559|protein_coding	3.675471268	6.812618331	1.553161	7.65E-05
	HIST1H4A|ENSG00000278637|protein_coding	0.47745762	1.213962048	1.348654	7.82E-05
	AL928654.3|ENSG00000257341|protein_coding	0.701651778	1.480322279	1.0708	8.20E-05
	DDX53|ENSG00000184735|protein_coding	0.806755876	3.467968173	2.291538	8.62E-05
	OTOP3|ENSG00000182938|protein_coding	3.599132312	12.44214553	1.345604	0.000101
	UGT1A1|ENSG00000241635|protein_coding	12.98410829	46.91576673	1.522706	0.000116
	UGT2B17|ENSG00000197888|protein_coding	9.092144507	17.36089317	1.154037	0.000134

	ABCC11|ENSG00000121270|protein_coding	612.7897259	972.4698512	1.022437	0.000141
	SCRT2|ENSG00000215397|protein_coding	0.290477693	9.025470505	2.121507	0.000214
	ANKRD30B|ENSG00000180777|protein_coding	13.53889086	18.9886804	1.017868	0.00023
	TLX1|ENSG00000107807|protein_coding	2.257885925	5.981935213	1.066541	0.000263
	TLX2|ENSG00000115297|protein_coding	1.308411064	2.610243724	1.033827	0.000296
	OR51D1|ENSG00000197428|protein_coding	2.920398252	6.405892059	1.14756	0.000297
	PSCA|ENSG00000167653|protein_coding	7733.1153	16119.15048	1.059666	0.000419
	HIST1H3I|ENSG00000275379|protein_coding	0.601360207	3.259474327	1.51786	0.000429
	LRRTM3|ENSG00000198739|protein_coding	6.651276311	33.6916746	1.055989	0.000439
	CARD18|ENSG00000255501|protein_coding	0.315887122	10.89041387	3.138526	0.000507
	TGM6|ENSG00000166948|protein_coding	1.758196089	2.532490419	1.056457	0.000531
	ADGB|ENSG00000118492|protein_coding	6.773775065	8.099581556	1.023455	0.000572
	HIST1H3F|ENSG00000277775|protein_coding	1.289411693	2.645032917	1.024478	0.000573
	PLAC1|ENSG00000170965|protein_coding	1.66324769	3.389720705	1.050852	0.000681
	RP1|ENSG00000104237|protein_coding	2.158738222	3.795483563	1.119381	0.000698
	C9orf135|ENSG00000204711|protein_coding	2.955773224	6.195641557	1.084103	0.000894
	ANKRD34B|ENSG00000189127|protein_coding	377.2573853	823.9382616	1.12696	0.000925
	CCDC166|ENSG00000255181|protein_coding	0.512391747	1.107018955	1.091715	0.001093
	CHAT|ENSG00000070748|protein_coding	3.000920514	32.7100541	1.395798	0.00134
	OR1Q1|ENSG00000165202|protein_coding	0.409479518	1.034700275	1.157107	0.002175
	OR1J1|ENSG00000136834|protein_coding	0.381791068	0.901484714	1.140166	0.002326
	LHX9|ENSG00000143355|protein_coding	9.042210941	24.33526657	1.027462	0.002502

	CRISP3|ENSG00000096006|protein_coding	7842.244656	16311.42259	1.056543	0.002611
	ANKRD30A|ENSG00000148513|protein_coding	41.92723925	93.63940313	1.337625	0.002686
	SERPINA4|ENSG00000100665|protein_coding	85.05830033	89.81011418	1.115328	0.002888
	GPR149|ENSG00000174948|protein_coding	3.768578485	7.955490455	1.730461	0.003191
	CLPSL1|ENSG00000204140|protein_coding	1.301025278	2.612442586	1.40674	0.003257
	IAPP|ENSG00000121351|protein_coding	27.4523073	31.01489851	1.233546	0.003311
	LHFPL5|ENSG00000197753|protein_coding	0.629802595	1.383703782	1.121535	0.003388
	B3GNT6|ENSG00000198488|protein_coding	531.2110062	1085.183366	1.030501	0.003998
	C16orf92|ENSG00000167194|protein_coding	0.238663266	0.700118247	1.272965	0.004234
	MAGEA12|ENSG00000213401|protein_coding	5.34147082	11.95462276	1.229727	0.00505
	KLHL1|ENSG00000150361|protein_coding	10.79917182	32.5829923	1.035677	0.00518
	SLC13A1|ENSG00000081800|protein_coding	0.605887349	1.310081564	1.088826	0.00557
	FDCSP|ENSG00000181617|protein_coding	16.08770245	25.42384894	1.167052	0.00557
	CSAG2|ENSG00000268902|protein_coding	1.102275719	1.647537999	1.000313	0.006411
	SIM1|ENSG00000112246|protein_coding	1.561402609	6.517429629	1.119748	0.006499
	DYTN|ENSG00000232125|protein_coding	27.35588225	57.76317913	1.079343	0.008531
	PLA2G2E|ENSG00000188784|protein_coding	0.834453457	1.945395978	1.236242	0.009904
	SLC17A1|ENSG00000124568|protein_coding	1.244916647	2.549702622	1.097167	0.011641
	CT45A10|ENSG00000269586|protein_coding	0.119802569	10.22385892	3.067674	0.012609
	REG1A|ENSG00000115386|protein_coding	10.61963215	21.40554538	1.197719	0.013831
	MAGEA6|ENSG00000197172|protein_coding	8.093728173	18.32006368	2.034949	0.014617
	UGT2A3|ENSG00000135220|protein_coding	0.780726231	6.611336347	1.524122	0.018595

	APOH|ENSG00000091583|protein_coding	22.30775308	57.82441037	1.329214	0.020076
	DCAF4L2|ENSG00000176566|protein_coding	2.389995928	7.027840399	2.345518	0.020658
	CPN1|ENSG00000120054|protein_coding	2.013116707	4.278660022	1.179894	0.023129
	FGA|ENSG00000171560|protein_coding	19.33134206	35.36733038	1.787218	0.026852
	SPRR2F|ENSG00000244094|protein_coding	0.941306052	2.165465937	1.210606	0.027787
	ASIC5|ENSG00000256394|protein_coding	4.595709637	7.312950034	1.222933	0.032246
	C3orf30|ENSG00000163424|protein_coding	0.928427758	3.232051142	1.383048	0.032316
	IFNL3|ENSG00000197110|protein_coding	0.219013501	0.654344007	1.282685	0.032919
	AKR1C4|ENSG00000198610|protein_coding	1.230094615	1.976567025	1.041173	0.033473
	HRG|ENSG00000113905|protein_coding	3.245466087	3.755477239	1.077301	0.036557
	APOA2|ENSG00000158874|protein_coding	17.70761012	44.97787907	1.497373	0.037169
	ZNF280A|ENSG00000169548|protein_coding	0.22866832	0.592914862	1.101173	0.039035
	TRIM49B|ENSG00000182053|protein_coding	0.377307279	1.994111303	1.223879	0.040085
	SLC18A3|ENSG00000187714|protein_coding	13.54654393	48.17690044	1.005844	0.044434
	PAH|ENSG00000171759|protein_coding	212.2538632	43.74090043	-2.8991	2.48E-23
	PROK1|ENSG00000143125|protein_coding	231.8181409	80.30976022	-1.52928	1.27E-15
	FMOD|ENSG00000122176|protein_coding	15898.7405	7917.205683	-1.00586	2.31E-13
	CHI3L2|ENSG00000064886|protein_coding	573.8972866	178.2515231	-1.6869	3.42E-13
	CHRNA2|ENSG00000120903|protein_coding	9460.848073	4599.241112	-1.04059	8.16E-12
	CDC42EP5|ENSG00000167617|protein_coding	2176.388791	1011.224608	-1.10581	1.81E-11
	CA14|ENSG00000118298|protein_coding	116.7423807	58.15671704	-1.00531	1.81E-11
	CPNE6|ENSG00000100884|protein_coding	166.359627	61.36188108	-1.43822	2.08E-11

	RPE65|ENSG00000116745|protein_coding	47.31579773	15.89098367	-1.57447	2.82E-11
	SCGB3A1|ENSG00000161055|protein_coding	240.0993377	152.9084395	-1.42024	1.48E-10
	CD38|ENSG00000004468|protein_coding	3618.286695	1422.528531	-1.34684	3.98E-10
	SAA2|ENSG00000134339|protein_coding	261.2713305	105.0963787	-1.65738	4.60E-10
	IGSF1|ENSG00000147255|protein_coding	122.0917554	53.65197093	-1.18516	5.10E-10
	PHYHD1|ENSG00000175287|protein_coding	2029.505762	994.4860385	-1.02905	1.29E-09
	SLC5A8|ENSG00000256870|protein_coding	118.8836569	43.2515031	-1.45718	1.33E-09
	AL049839.2|ENSG00000273259|protein_coding	45.23110469	24.40884042	-1.26046	1.44E-09
	TG|ENSG00000042832|protein_coding	1729.514954	305.2280415	-1.30603	1.60E-09
	RAMACL|ENSG00000235272|protein_coding	8.384769968	4.098970946	-1.62767	1.77E-09
	LY6G6F-LY6G6D|ENSG00000250641|protein_coding	12.2102142	10.42559572	-1.28534	3.12E-09
	SLC18A2|ENSG00000165646|protein_coding	139.0643671	63.91862005	-1.1207	4.95E-09
	GFAP|ENSG00000131095|protein_coding	84.97054922	58.36063347	-1.35268	4.97E-09
	LY6G6D|ENSG00000244355|protein_coding	11.99064355	9.74760828	-1.25434	1.37E-08
	INSYN2B|ENSG00000204767|protein_coding	10.91492027	4.375502118	-1.3142	2.23E-08
	NPR3|ENSG00000113389|protein_coding	3971.871291	1747.324996	-1.18468	3.15E-08
	IP6K3|ENSG00000161896|protein_coding	140.9029883	67.16882186	-1.06755	3.44E-08
	ORM1|ENSG00000229314|protein_coding	6886.633853	4465.874095	-2.23701	3.94E-08
	LCN2|ENSG00000148346|protein_coding	3600.681074	1457.620794	-1.30469	4.04E-08
	FAM3D|ENSG00000198643|protein_coding	2277.328522	1114.023621	-1.03152	6.76E-08
	PEBP4|ENSG00000134020|protein_coding	2007.733812	943.232648	-1.08995	6.85E-08
	SLC7A14|ENSG00000013293|protein_coding	12.52553364	9.019873192	-1.06413	1.14E-07

	MAB21L1|ENSG00000180660|protein_coding	136.952063	52.79801229	-1.2371	1.63E-07
	ANPEP|ENSG00000166825|protein_coding	27862.6639	11227.60992	-1.31128	1.75E-07
	CLCA2|ENSG00000137975|protein_coding	137.9429572	67.82325713	-1.34971	1.80E-07
	GABRQ|ENSG00000268089|protein_coding	79.55508425	38.5857124	-1.0427	1.92E-07
	TMEM213|ENSG00000214128|protein_coding	19.42977689	10.48475978	-1.33068	2.09E-07
	CXCL5|ENSG00000163735|protein_coding	140.5227733	63.85481741	-1.38199	2.10E-07
	EDN3|ENSG00000124205|protein_coding	109.7523056	87.6828868	-1.06484	2.31E-07
	KCTD8|ENSG00000183783|protein_coding	45.00198108	21.68149094	-1.05665	2.59E-07
	LRTM1|ENSG00000144771|protein_coding	16.8743918	3.486405057	-2.47322	2.98E-07
	CKM|ENSG00000104879|protein_coding	2136.636615	685.5543647	-1.34474	5.00E-07
	CST4|ENSG00000101441|protein_coding	49.08227609	85.37104137	-1.26948	5.14E-07
	SLC7A4|ENSG00000099960|protein_coding	408.7607077	186.0424316	-1.13547	7.91E-07
	MGAM2|ENSG00000257743|protein_coding	15.58605713	7.014883467	-1.14885	9.88E-07
	ITLN2|ENSG00000158764|protein_coding	72.22674445	38.59050026	-1.00773	1.88E-06
	CRYBA4|ENSG00000196431|protein_coding	23.64276729	13.3361026	-1.78776	2.91E-06
	ARSF|ENSG00000062096|protein_coding	4.823620567	2.151419695	-1.15849	3.39E-06
	GPX5|ENSG00000224586|protein_coding	21.56406375	10.6507571	-1.39353	5.21E-06
	SLC1A6|ENSG00000105143|protein_coding	6.436020861	3.208578549	-1.00652	6.18E-06
	GRP|ENSG00000134443|protein_coding	126.0880585	29.35698312	-1.02678	7.24E-06
	PON1|ENSG00000005421|protein_coding	230.2983854	148.0976369	-1.49985	8.72E-06
	SERPINA3|ENSG00000196136|protein_coding	326.1700055	158.7072784	-1.03916	9.36E-06
	VGLL3|ENSG00000206538|protein_coding	6276.393297	2723.913516	-1.20424	9.43E-06

	PCARE|ENSG00000179270|protein_coding	7.951417216	3.72998934	-1.0711	1.01E-05
	ZNF648|ENSG00000179930|protein_coding	2.175493853	0.875036071	-1.32054	1.15E-05
	CLEC4M|ENSG00000104938|protein_coding	2.642978871	1.06467621	-1.26992	1.27E-05
	CDH18|ENSG00000145526|protein_coding	9.731659525	4.63772106	-1.0633	1.61E-05
	CCDC169|ENSG00000242715|protein_coding	311.8613863	154.0441397	-1.01724	1.67E-05
	ACTA1|ENSG00000143632|protein_coding	5365.125219	1394.117146	-1.35455	2.11E-05
	NRAP|ENSG00000197893|protein_coding	1631.027923	511.1379276	-1.6091	2.15E-05
	OLFM4|ENSG00000102837|protein_coding	8881.471054	3773.516552	-1.2349	2.32E-05
	KRT9|ENSG00000171403|protein_coding	3.758238807	1.717007351	-1.11802	2.54E-05
	RLN1|ENSG00000107018|protein_coding	5364.891497	2010.533849	-1.41598	3.77E-05
	CYP4F2|ENSG00000186115|protein_coding	309.3446644	488.0610562	-1.31562	4.38E-05
	SAA2-SAA4|ENSG00000255071|protein_coding	28.65822492	11.06668552	-1.3894	4.48E-05
	CARTPT|ENSG00000164326|protein_coding	150.713089	55.12515412	-1.06908	4.93E-05
	SLC39A2|ENSG00000165794|protein_coding	93.6852979	73.72114726	-1.1586	7.32E-05
	TECTB|ENSG00000119913|protein_coding	14.11410998	18.6855278	-1.10169	7.89E-05
	SAA1|ENSG00000173432|protein_coding	727.3825565	321.5009027	-1.05816	9.15E-05
	KCNG4|ENSG00000168418|protein_coding	1.107736888	0.430225977	-1.21604	9.15E-05
	ATP6V0A4|ENSG00000105929|protein_coding	38.01498318	23.24280486	-1.02766	0.000104
	SOHLH2|ENSG00000120669|protein_coding	76.58939058	37.43480831	-1.0322	0.000108
	SMCO1|ENSG00000214097|protein_coding	10.51623179	3.68422124	-1.18461	0.000143
	UNC45B|ENSG00000141161|protein_coding	85.66893047	38.60252523	-1.14755	0.000157
	CCK|ENSG00000187094|protein_coding	487.2643207	236.2525866	-1.04426	0.000162

	PADI3|ENSG00000142619|protein_coding	23.00382365	13.21944642	-1.21891	0.00017
	AL645922.1|ENSG00000244255|protein_coding	1.229628224	0.499431152	-1.24265	0.000179
	SMR3B|ENSG00000171201|protein_coding	8.113622766	1.253961016	-2.55959	0.000179
	MT1B|ENSG00000169688|protein_coding	5.91281192	2.874779366	-1.03093	0.000209
	SYNPO2L|ENSG00000166317|protein_coding	210.8570592	83.90491712	-1.13823	0.000238
	MYOZ2|ENSG00000172399|protein_coding	187.6661135	56.46269316	-1.07962	0.000242
	GPR101|ENSG00000165370|protein_coding	1.121478525	0.676456587	-1.12937	0.0003
	FOXI1|ENSG00000168269|protein_coding	97.08138605	60.6769044	-1.17504	0.000411
	GCG|ENSG00000115263|protein_coding	62.38311399	31.16127426	-1.00294	0.000413
	TMPRSS11A|ENSG00000187054|protein_coding	12.92899992	6.609850358	-1.16014	0.000989
	LTF|ENSG00000012223|protein_coding	53258.25965	26478.99387	-1.00816	0.000994
	KRT76|ENSG00000185069|protein_coding	1.124908297	0.223242363	-1.89764	0.001043
	AQP2|ENSG00000167580|protein_coding	15.1401635	27.38073868	-1.16017	0.001295
	CCDC169-SOHLH2|ENSG00000250709|protein_coding	10.1949359	5.060205381	-1.00992	0.001463
	PRB2|ENSG00000121335|protein_coding	3.034004886	2.351427613	-1.15953	0.001485
	XIRP1|ENSG00000168334|protein_coding	407.8698325	130.7846201	-1.13502	0.001579
	MUC21|ENSG00000204544|protein_coding	6.042221701	2.701674889	-1.17082	0.003069
	MYH7|ENSG00000092054|protein_coding	9037.346818	2264.53185	-1.54054	0.003158
	CFC1B|ENSG00000152093|protein_coding	7.112607503	1.509607433	-1.23282	0.003182
	COL6A5|ENSG00000172752|protein_coding	6.10821019	1.61349633	-1.17752	0.003241
	ATP1B4|ENSG00000101892|protein_coding	35.15788383	14.78485786	-1.26883	0.003945
	AGBL1|ENSG00000273540|protein_coding	6.323147273	2.800885132	-1.24167	0.004337

	TRDN|ENSG00000186439|protein_coding	431.9605221	118.022926	-1.1465	0.004815
	HJV|ENSG00000168509|protein_coding	33.68809191	11.00055225	-1.01048	0.005489
	MT4|ENSG00000102891|protein_coding	7.044827694	0.736210661	-2.45756	0.005586
	ASB10|ENSG00000146926|protein_coding	10.99816741	3.872547751	-1.33213	0.005918
	MYL2|ENSG00000111245|protein_coding	3405.787571	908.3121277	-1.59721	0.006515
	FBXO40|ENSG00000163833|protein_coding	52.79586364	21.67732631	-1.27171	0.006742
	C10orf71|ENSG00000177354|protein_coding	103.1343892	37.98173709	-1.13185	0.009313
	DHRS7C|ENSG00000184544|protein_coding	4.263257001	1.684705228	-1.59992	0.009503
	CCDC63|ENSG00000173093|protein_coding	2.525748552	0.916000917	-1.21202	0.009872
	MYL1|ENSG00000168530|protein_coding	1608.964058	465.820773	-1.81765	0.0102
	LINC02203|ENSG00000280709|protein_coding	1.615150367	0.724133182	-1.16565	0.013417
	XIRP2|ENSG00000163092|protein_coding	782.9509806	250.8833846	-1.73862	0.015205
	PRDM13|ENSG00000112238|protein_coding	0.875138597	0.356388349	-1.24274	0.015351
	SPRR3|ENSG00000163209|protein_coding	6.993878582	2.64995672	-1.11434	0.017151
	SBK2|ENSG00000187550|protein_coding	1.27510555	0.355635153	-1.13642	0.019303
	KLHL40|ENSG00000157119|protein_coding	101.1023557	35.01172228	-1.17705	0.019811
	LMOD2|ENSG00000170807|protein_coding	388.0128118	105.735601	-1.13463	0.022634
	PPP1R3A|ENSG00000154415|protein_coding	21.77228018	7.325828463	-2.20773	0.030958
	DUPD1|ENSG00000188716|protein_coding	8.150130053	2.781372617	-1.51904	0.041382
	OR10H2|ENSG00000171942|protein_coding	0.651935575	0.216054679	-1.16703	0.042047
	SPAG11A|ENSG00000178287|protein_coding	1.625139204	0.392717327	-1.34003	0.045724
	OR1N2|ENSG00000171501|protein_coding	6.781910745	2.104023259	-1.89915	0.047502
