# Supplementary material for: An Integrative Multi-Omics Analysis Based on Nomogram for Predicting Prostate Cancer Bone Metastasis Incidence
Source: Genet Res (Camb). 2022 Sep 29;2022:8213723. doi: 10.1155/2022/8213723 (PMC9537037; doi:10.1155/2022/8213723)
Supplement: Supplementary Materials — Figure S1: associated with Figure 2. Figure S2: associated with Figure 5. Figure S3: associated with Figure 6. Data 1-1: SEER data baseline characteristics of patient with prostate cancer; Data 1-2: train group ROC and validation ROC, Delong's test; Data 1-3: clinical features of TCGA samples and predictive results. Data 2: TMB scores of TCGA samples. Data 3: genes with different frequencies of CNVs between LRBM and HRBM cohorts. Data 4-1: DElncRNAs; Data 4-2: DEmiRNAs; Data 4-3: DEmRNAs. Data 5: gene module. Data 6-1: 29 immune microenvironment signatures; Data-2: ssGSEA score of immune microenvironment signature; Data 6-3: stromal score, immune score, estimate score, and tumor purity of TCGA samples. [file 8213723.f1.zip › 8213723.f1/supplementary data5 (1).pdf]

**Supplementary Data5:gene module**						
	**datSummary**	**colorNEW**	**kMEblue**	**kMEbrown**	**kMEgrey**	**kMEturquoise**
	**FAM72D|ENSG00000215784|protein_coding**	grey	0.708538	0.130757	0.74014	-0.03295
	**SAA2|ENSG00000134339|protein_coding**	grey	-0.02689	-0.016215	-0.06813	0.068339
	**SYNPO2L|ENSG00000166317|protein_coding**	turquoise	-0.04079	0.00295	-0.05209	0.980131
	**OR10H2|ENSG00000171942|protein_coding**	grey	0.020286	-0.024189	0.0031	0.012115
	**GPR101|ENSG00000165370|protein_coding**	grey	-0.0145	0.012201	-0.01935	-0.0198
	**MATN4|ENSG00000124159|protein_coding**	grey	0.137382	0.032197	0.179573	-0.02499
	**CLEC4M|ENSG00000104938|protein_coding**	grey	-0.05894	-0.008131	-0.09525	0.013686
	**PTK6|ENSG00000101213|protein_coding**	grey	0.190749	0.135568	0.273276	-0.01964
	**PLAC1|ENSG00000170965|protein_coding**	grey	0.113541	0.023664	0.155201	-0.02709
	**LHX2|ENSG00000106689|protein_coding**	grey	0.412536	0.075592	0.435859	-0.03519
	**LINC02203|ENSG00000280709|protein_coding**	grey	-0.05995	-0.059543	-0.06311	-0.00978
	**MYT1|ENSG00000196132|protein_coding**	grey	0.21321	0.0548	0.276522	-0.02923
	**BARX1|ENSG00000131668|protein_coding**	grey	0.198373	0.091532	0.28465	-0.03957
	**MGAM2|ENSG00000257743|protein_coding**	grey	0.036477	-0.011993	-0.0297	0.006753
	**UBE2C|ENSG00000175063|protein_coding**	grey	0.696829	0.137121	0.669936	-0.02347
	**SAA2-SAA4|ENSG00000255071|protein_coding**	grey	-0.02489	-0.013635	-0.06717	0.072517
	**CCDC169-SOHLH2|ENSG00000250709|protein_coding**	grey	0.010032	-0.02318	-0.04838	0.028994
	**OPRK1|ENSG00000082556|protein_coding**	grey	0.147786	0.051215	0.206227	-0.03618
	**CCDC63|ENSG00000173093|protein_coding**	turquoise	-0.03536	-0.007094	-0.03784	0.98056
	**SYT4|ENSG00000132872|protein_coding**	grey	0.411353	-0.004869	0.415204	-0.02723

<table>
<tr><td></td><td>WFDC5|ENSG00000175121|protein_coding</td><td>grey</td><td>0.417262</td><td>0.084452</td><td>0.403188</td><td>0.002522</td></tr>
<tr><td></td><td>OR51D1|ENSG00000197428|protein_coding</td><td>grey</td><td>0.083567</td><td>-0.025599</td><td>0.134769</td><td>-0.04136</td></tr>
<tr><td></td><td>RP1|ENSG00000104237|protein_coding</td><td>grey</td><td>0.170711</td><td>0.197935</td><td>0.21314</td><td>0.0005</td></tr>
<tr><td></td><td>NCAM2|ENSG00000154654|protein_coding</td><td>grey</td><td>0.083729</td><td>0.04519</td><td>0.121752</td><td>0.009838</td></tr>
<tr><td></td><td>XIRP2|ENSG00000163092|protein_coding</td><td>turquoise</td><td>-0.03731</td><td>0.003567</td><td>-0.04594</td><td>0.979414</td></tr>
<tr><td></td><td>PBK|ENSG00000168078|protein_coding</td><td>grey</td><td>0.855127</td><td>0.089476</td><td>0.828201</td><td>-0.05342</td></tr>
<tr><td></td><td>HOXB5|ENSG00000120075|protein_coding</td><td>grey</td><td>0.077946</td><td>0.061968</td><td>0.103341</td><td>0.020719</td></tr>
<tr><td></td><td>CENPF|ENSG00000117724|protein_coding</td><td>blue</td><td>0.968444</td><td>0.215681</td><td>0.918528</td><td>-0.04272</td></tr>
<tr><td></td><td>AGBL1|ENSG00000273540|protein_coding</td><td>turquoise</td><td>-0.06081</td><td>-0.008704</td><td>-0.06117</td><td>0.95484</td></tr>
<tr><td></td><td>CD38|ENSG00000004468|protein_coding</td><td>grey</td><td>-0.13826</td><td>-0.083584</td><td>-0.23631</td><td>-0.04821</td></tr>
<tr><td></td><td>MYBL2|ENSG00000101057|protein_coding</td><td>grey</td><td>0.873483</td><td>0.211091</td><td>0.893683</td><td>-0.03777</td></tr>
<tr><td></td><td>KIF14|ENSG00000118193|protein_coding</td><td>blue</td><td>0.961224</td><td>0.180317</td><td>0.899674</td><td>-0.0341</td></tr>
<tr><td></td><td>MAGEA1|ENSG00000198681|protein_coding</td><td>grey</td><td>-0.02489</td><td>-0.005069</td><td>-0.00457</td><td>-0.01444</td></tr>
<tr><td></td><td>TLX1|ENSG00000107807|protein_coding</td><td>grey</td><td>0.163426</td><td>0.040813</td><td>0.236071</td><td>-0.02364</td></tr>
<tr><td></td><td>CDK1|ENSG00000170312|protein_coding</td><td>grey</td><td>0.851966</td><td>0.16795</td><td>0.814787</td><td>-0.03922</td></tr>
<tr><td></td><td>SPRR3|ENSG00000163209|protein_coding</td><td>grey</td><td>-0.03676</td><td>-0.013636</td><td>-0.06744</td><td>-0.00085</td></tr>
<tr><td></td><td>IL36RN|ENSG00000136695|protein_coding</td><td>grey</td><td>0.4655</td><td>0.08981</td><td>0.492637</td><td>-0.02474</td></tr>
<tr><td></td><td>CARD18|ENSG00000255501|protein_coding</td><td>grey</td><td>-0.00095</td><td>0.018374</td><td>0.015416</td><td>-0.01087</td></tr>
<tr><td></td><td>SAA1|ENSG00000173432|protein_coding</td><td>grey</td><td>-0.04396</td><td>-0.021841</td><td>-0.08525</td><td>0.059488</td></tr>
<tr><td></td><td>ESPL1|ENSG00000135476|protein_coding</td><td>blue</td><td>0.95722</td><td>0.18191</td><td>0.918147</td><td>-0.04512</td></tr>
<tr><td></td><td>CST2|ENSG00000170369|protein_coding</td><td>grey</td><td>0.00796</td><td>0.023809</td><td>0.044999</td><td>-0.01983</td></tr>
<tr><td></td><td>HJURP|ENSG00000123485|protein_coding</td><td>grey</td><td>0.84462</td><td>0.168602</td><td>0.85183</td><td>-0.04279</td></tr>
</table>

	**SERPINA4|ENSG00000100665|protein_coding**	grey	-0.01022	-0.03137	0.028228	-0.02859
	**POLQ|ENSG00000051341|protein_coding**	grey	0.921509	0.203037	0.906684	-0.04798
	**TICRR|ENSG00000140534|protein_coding**	grey	0.893099	0.199134	0.869845	-0.05358
	**SCGB3A1|ENSG00000161055|protein_coding**	grey	-0.08938	-0.041125	-0.11336	-0.01793
	**DGKK|ENSG00000274588|protein_coding**	grey	0.212273	0.001726	0.200031	-0.01552
	**KIF15|ENSG00000163808|protein_coding**	grey	0.901807	0.178059	0.875054	-0.05769
	**TTC29|ENSG00000137473|protein_coding**	grey	0.125814	0.061689	0.193026	-0.02887
	**AL645922.1|ENSG00000244255|protein_coding**	grey	-0.00752	0.008677	-0.07837	0.041142
	**ASF1B|ENSG00000105011|protein_coding**	grey	0.894014	0.157184	0.904168	-0.05795
	**SLC18A2|ENSG00000165646|protein_coding**	grey	-0.08333	-0.042052	-0.17661	-0.03323
	**OTOP3|ENSG00000182938|protein_coding**	grey	0.02678	0.00381	0.073428	-0.01252
	**TOP2A|ENSG00000131747|protein_coding**	blue	0.973786	0.196228	0.907167	-0.04434
	**TG|ENSG00000042832|protein_coding**	grey	-0.01793	0.017026	-0.0288	-0.00989
	**GPR149|ENSG00000174948|protein_coding**	grey	0.122607	0.01528	0.14095	-0.00849
	**KIF18B|ENSG00000186185|protein_coding**	grey	0.934042	0.154176	0.938413	-0.03887
	**NRAP|ENSG00000197893|protein_coding**	turquoise	-0.04943	-0.001148	-0.0593	0.994551
	**SBK2|ENSG00000187550|protein_coding**	grey	-0.0353	-0.015049	-0.04528	0.407534
	**AL928654.3|ENSG00000257341|protein_coding**	grey	0.097383	0.063926	0.135174	0.064835
	**MYL2|ENSG00000111245|protein_coding**	turquoise	-0.04548	-0.00412	-0.04855	0.990211
	**IFI44L|ENSG00000137959|protein_coding**	grey	0.267554	0.207362	0.336605	-0.02725
	**TGM6|ENSG00000166948|protein_coding**	grey	0.121361	0.010676	0.110427	0.004149
	**UHRF1|ENSG00000276043|protein_coding**	grey	0.905331	0.172366	0.889679	-0.0481

	**KRT9|ENSG00000171403|protein_coding**	grey	-0.15486	-0.0672	-0.21914	0.009507
	**SLC17A1|ENSG00000124568|protein_coding**	grey	0.055087	-0.032692	0.120819	-0.02849
	**OR1N2|ENSG00000171501|protein_coding**	grey	-0.01668	-0.017087	-0.01195	0.004853
	**KCNK4|ENSG00000182450|protein_coding**	grey	0.109006	0.096813	0.202559	-0.0155
	**DEPDC1B|ENSG00000035499|protein_coding**	grey	0.539815	0.096577	0.533996	-0.04188
	**SERPINI1|ENSG00000163536|protein_coding**	grey	0.063722	0.085572	0.154145	-0.01364
	**RAMACL|ENSG00000235272|protein_coding**	grey	-0.00203	-0.003917	-0.03258	0.081519
	**STMN2|ENSG00000104435|protein_coding**	grey	0.160832	0.538057	0.191203	-0.02438
	**CDKN3|ENSG00000100526|protein_coding**	grey	0.919004	0.18494	0.925067	-0.02731
	**ODF3L2|ENSG00000181781|protein_coding**	grey	0.147451	0.035137	0.234618	-0.02196
	**SOX11|ENSG00000176887|protein_coding**	grey	0.377182	0.650935	0.393464	0.020068
	**PRB2|ENSG00000121335|protein_coding**	grey	0.004867	-0.016391	-0.00277	-0.01572
	**AKR1C4|ENSG00000198610|protein_coding**	grey	0.064403	-0.002128	0.137104	-0.02227
	**KRT75|ENSG00000170454|protein_coding**	grey	0.181045	0.637762	0.241309	-0.02421
	**CRISP3|ENSG00000096006|protein_coding**	grey	0.052625	0.021676	0.069283	-0.04431
	**SERPINA3|ENSG00000196136|protein_coding**	grey	-0.05393	-0.023237	-0.09422	0.101524
	**GRIK1|ENSG00000171189|protein_coding**	grey	0.082945	-0.002262	0.126186	-0.01593
	**VGLL3|ENSG00000206538|protein_coding**	grey	-0.0346	-0.024528	-0.0758	0.037684
	**GFAP|ENSG00000131095|protein_coding**	grey	0.028779	0.029377	0.030355	0.022865
	**NEK2|ENSG00000117650|protein_coding**	blue	0.949871	0.18728	0.932826	-0.05233
	**ADAM18|ENSG00000168619|protein_coding**	grey	0.343062	0.091019	0.389996	-0.04126
	**PCARE|ENSG00000179270|protein_coding**	grey	-0.12075	-0.031477	-0.16507	0.800459

	**COL10A1|ENSG00000123500|protein_coding**	brown	0.155687	0.980714	0.188752	-0.01704
	**GCG|ENSG00000115263|protein_coding**	grey	-0.05735	-0.030207	-0.07317	-0.03077
	**GABRQ|ENSG00000268089|protein_coding**	grey	-0.1696	-0.063881	-0.25829	-0.04272
	**ACTA1|ENSG00000143632|protein_coding**	turquoise	-0.049	-0.004692	-0.05121	0.98172
	**ADGB|ENSG00000118492|protein_coding**	grey	0.113286	0.061245	0.151561	-0.02668
	**RPE65|ENSG00000116745|protein_coding**	grey	-0.17768	-0.09973	-0.30298	-0.05371
	**UGT2B17|ENSG00000197888|protein_coding**	grey	0.201595	0.100638	0.269552	-0.0317
	**SLC7A14|ENSG00000013293|protein_coding**	grey	-0.04475	-0.013951	-0.09424	-0.01842
	**TPX2|ENSG00000088325|protein_coding**	blue	0.977578	0.203211	0.945894	-0.0413
	**ATP1B4|ENSG00000101892|protein_coding**	turquoise	-0.04409	0.006045	-0.05565	0.924055
	**CRYBA4|ENSG00000196431|protein_coding**	grey	-0.03729	0.015893	-0.04086	0.053883
	**CDC25C|ENSG00000158402|protein_coding**	grey	0.8884	0.14687	0.882626	-0.04553
	**DNAH8|ENSG00000124721|protein_coding**	grey	0.119943	0.014765	0.156316	-0.0232
	**PLEKHD1|ENSG00000175985|protein_coding**	grey	0.271735	0.055787	0.391603	-0.03313
	**ZBBX|ENSG00000169064|protein_coding**	grey	0.139037	0.069963	0.22803	-0.0336
	**SMR3B|ENSG00000171201|protein_coding**	grey	-0.01238	-0.023858	-0.03018	-0.00701
	**SLC1A6|ENSG00000105143|protein_coding**	grey	-0.12813	-0.047182	-0.19258	-0.03415
	**RAD54L|ENSG00000085999|protein_coding**	grey	0.935096	0.170306	0.911579	-0.04282
	**GTSE1|ENSG00000075218|protein_coding**	grey	0.899273	0.165303	0.879132	-0.05266
	**IAPP|ENSG00000121351|protein_coding**	grey	0.033002	0.003837	0.067857	-0.0211
	**C6orf10|ENSG00000204296|protein_coding**	grey	0.166274	0.062127	0.195446	-0.01271
	**MT4|ENSG00000102891|protein_coding**	grey	-0.0182	0.006024	-0.00392	-0.00179

	**PDIA2|ENSG00000185615|protein_coding**	grey	0.090125	0.031526	0.183816	0.00121
	**CENPA|ENSG00000115163|protein_coding**	grey	0.9227	0.238402	0.936682	-0.04129
	**BRS3|ENSG00000102239|protein_coding**	grey	0.37952	0.254822	0.458989	0.003061
	**TUBA4B|ENSG00000243910|protein_coding**	grey	0.060203	0.03414	0.109826	-0.02777
	**MELK|ENSG00000165304|protein_coding**	grey	0.941839	0.185121	0.928574	-0.05819
	**NUF2|ENSG00000143228|protein_coding**	grey	0.921289	0.167968	0.916058	-0.04817
	**ORC6|ENSG00000091651|protein_coding**	grey	0.890594	0.165333	0.892514	-0.01843
	**MAGEA6|ENSG00000197172|protein_coding**	grey	0.30395	0.086053	0.299409	-0.01457
	**C16orf92|ENSG00000167194|protein_coding**	grey	0.219099	0.008705	0.291118	-0.01639
	**ANLN|ENSG00000011426|protein_coding**	grey	0.846243	0.195099	0.824143	-0.04134
	**GPX5|ENSG00000224586|protein_coding**	grey	-0.01539	0.001584	-0.05283	0.050304
	**CCDC169|ENSG00000242715|protein_coding**	grey	-0.02775	-0.058883	-0.07209	0.020796
	**FCRL2|ENSG00000132704|protein_coding**	grey	0.15349	0.127723	0.143955	-0.01703
	**TM4SF20|ENSG00000168955|protein_coding**	grey	0.07718	-0.000259	0.149507	-0.01278
	**CST4|ENSG00000101441|protein_coding**	grey	0.010049	0.055802	0.045829	-0.01622
	**DHRS7C|ENSG00000184544|protein_coding**	turquoise	-0.05142	-0.006688	-0.05396	0.988488
	**CFC1B|ENSG00000152093|protein_coding**	grey	-0.02428	-0.021585	-0.02401	-0.01139
	**SCRT2|ENSG00000215397|protein_coding**	grey	0.011094	-6.36E-05	0.062476	-0.00335
	**FAM3D|ENSG00000198643|protein_coding**	grey	-0.07355	0.025434	-0.08679	-0.01435
	**SHCBP1|ENSG00000171241|protein_coding**	grey	0.894021	0.219479	0.862249	-0.01818
	**TUNAR|ENSG00000250366|protein_coding**	grey	0.517255	0.038918	0.455629	-0.02359
	**PNMA5|ENSG00000198883|protein_coding**	grey	0.019029	-0.010944	0.04405	-0.00836

	**ASPM|ENSG00000066279|protein_coding**	blue	0.971849	0.208505	0.89995	-0.03785
	**NKX6-1|ENSG00000163623|protein_coding**	grey	0.159841	0.005951	0.241697	-0.04104
	**TRPC5OS|ENSG00000204025|protein_coding**	grey	0.29757	0.093718	0.343124	-0.06101
	**PHYHD1|ENSG00000175287|protein_coding**	grey	-0.23371	-0.121393	-0.30077	0.078901
	**LRTM1|ENSG00000144771|protein_coding**	turquoise	-0.0597	-0.009214	-0.06979	0.901007
	**KLK14|ENSG00000129437|protein_coding**	grey	0.124204	0.002032	0.15129	-0.03054
	**ZNF648|ENSG00000179930|protein_coding**	grey	0.023088	-0.010453	-0.02359	0.051982
	**LY6G6F-LY6G6D|ENSG00000250641|protein_coding**	grey	-0.04523	0.051538	-0.04857	-0.00379
	**KCNG4|ENSG00000168418|protein_coding**	grey	-0.10726	-0.066779	-0.16311	0.000665
	**RDM1|ENSG00000278023|protein_coding**	grey	0.760011	0.12009	0.779553	-0.03804
	**AP002373.1|ENSG00000255663|protein_coding**	grey	0.173329	0.090362	0.268834	-0.04479
	**DHRS2|ENSG00000100867|protein_coding**	grey	0.042282	0.047303	0.073245	-0.00775
	**SLC17A4|ENSG00000146039|protein_coding**	grey	0.112228	-0.015027	0.174076	-0.03174
	**MT1B|ENSG00000169688|protein_coding**	grey	-0.13623	-0.074628	-0.18367	0.026196
	**OLFM4|ENSG00000102837|protein_coding**	grey	-0.03756	-0.043621	-0.09372	-0.01859
	**HOXD1|ENSG00000128645|protein_coding**	grey	0.310006	0.040793	0.352186	-0.02553
	**HJV|ENSG00000168509|protein_coding**	turquoise	-0.04709	-0.005024	-0.0485	0.934069
	**MUC6|ENSG00000184956|protein_coding**	grey	0.114141	0.056024	0.152238	-0.02274
	**MUC21|ENSG00000204544|protein_coding**	grey	-0.0826	-0.028155	-0.11922	-0.02218
	**PEBP4|ENSG00000134020|protein_coding**	grey	-0.23883	-0.118482	-0.30079	0.007713
	**RLN1|ENSG00000107018|protein_coding**	grey	-0.09799	-0.03596	-0.11209	-0.03073
	**CEP55|ENSG00000138180|protein_coding**	grey	0.898462	0.179698	0.86967	-0.03958

	**SLC18A3|ENSG00000187714|protein_coding**	grey	0.113132	0.01025	0.193473	-0.03098
	**HIST1H2AJ|ENSG00000276368|protein_coding**	grey	0.334259	0.090274	0.390295	-0.03536
	**FGB|ENSG00000171564|protein_coding**	grey	-0.00945	-0.019269	0.009013	-0.01368
	**MUC1|ENSG00000185499|protein_coding**	grey	0.125197	0.071202	0.177486	-0.02559
	**TECTB|ENSG00000119913|protein_coding**	grey	0.024014	-0.024185	0.025764	0.018497
	**FNDC1|ENSG00000164694|protein_coding**	brown	0.255026	0.952797	0.267988	0.015937
	**ITLN2|ENSG00000158764|protein_coding**	grey	-0.02064	0.009224	-0.0338	0.008105
	**CT45A10|ENSG00000269586|protein_coding**	grey	0.017205	-0.010237	0.073774	-0.01118
	**ASIC5|ENSG00000256394|protein_coding**	grey	0.027581	0.058756	0.061125	-0.00877
	**HOXB6|ENSG00000108511|protein_coding**	grey	0.141393	0.091146	0.17651	0.018939
	**C5orf46|ENSG00000178776|protein_coding**	grey	0.051822	0.192751	0.078533	-0.02808
	**IP6K3|ENSG00000161896|protein_coding**	grey	-0.18741	-0.065703	-0.27394	0.647938
	**ANPEP|ENSG00000166825|protein_coding**	grey	-0.14138	-0.083093	-0.20736	0.057442
	**LRRTM3|ENSG00000198739|protein_coding**	grey	0.36167	-0.014931	0.320821	-0.01049
	**XIRP1|ENSG00000168334|protein_coding**	turquoise	-0.04162	0.012655	-0.04353	0.968984
	**MAB21L2|ENSG00000181541|protein_coding**	grey	0.019247	0.034318	0.064919	-0.0093
	**CBX2|ENSG00000173894|protein_coding**	grey	0.467163	0.174715	0.548219	-0.02465
	**LIPF|ENSG00000182333|protein_coding**	grey	0.075306	0.016555	0.126332	-0.0075
	**PAEP|ENSG00000122133|protein_coding**	grey	0.037025	0.047819	0.02507	-0.01869
	**TEX19|ENSG00000182459|protein_coding**	grey	0.519131	0.108942	0.557572	-0.0108
	**DCAF4L2|ENSG00000176566|protein_coding**	grey	0.107683	0.02783	0.124235	-0.01431
	**DMBX1|ENSG00000197587|protein_coding**	grey	0.468513	0.208027	0.543045	-0.05383

	**IFNL3|ENSG00000197110|protein_coding**	grey	0.229892	0.014863	0.289172	-0.02658
	**NPR3|ENSG00000113389|protein_coding**	grey	-0.03386	-0.006839	-0.08249	0.030137
	**VWA5B1|ENSG00000158816|protein_coding**	grey	0.032444	0.00651	0.051992	-0.01692
	**REG1A|ENSG00000115386|protein_coding**	grey	-0.03626	-0.008455	-0.0191	-0.00815
	**TMEM213|ENSG00000214128|protein_coding**	grey	-0.09625	-0.048176	-0.15828	-0.02227
	**UGT2A3|ENSG00000135220|protein_coding**	grey	0.125863	0.039767	0.191906	-0.0075
	**ASB10|ENSG00000146926|protein_coding**	turquoise	-0.04778	-0.004591	-0.05369	0.993419
	**CARTPT|ENSG00000164326|protein_coding**	grey	-0.08787	-0.056803	-0.11827	-0.02642
	**CTAG2|ENSG00000126890|protein_coding**	grey	-0.03312	-0.010752	-0.03316	-0.01293
	**CYP4F2|ENSG00000186115|protein_coding**	grey	-0.0022	-0.030728	0.005791	-0.01415
	**SLC13A1|ENSG00000081800|protein_coding**	grey	0.068633	0.016059	0.098595	0.003967
	**AVPR1B|ENSG00000198049|protein_coding**	grey	0.055075	0.031301	0.134055	-0.02512
	**PKMYT1|ENSG00000127564|protein_coding**	grey	0.847265	0.109311	0.890955	-0.05759
	**HIST1H4A|ENSG00000278637|protein_coding**	grey	0.166865	0.08194	0.2171	-0.01438
	**PCDHGA5|ENSG00000253485|protein_coding**	grey	0.248916	0.104124	0.30075	-0.02536
	**CKAP2L|ENSG00000169607|protein_coding**	blue	0.975909	0.191879	0.912704	-0.04393
	**NCAPH|ENSG00000121152|protein_coding**	grey	0.832457	0.181358	0.804378	-0.01365
	**MAGEA8|ENSG00000156009|protein_coding**	grey	-0.02003	-0.001635	0.00206	0.003044
	**RRM2|ENSG00000171848|protein_coding**	grey	0.907141	0.235137	0.891903	-0.04912
	**BMP6|ENSG00000153162|protein_coding**	grey	0.552007	0.184363	0.60896	-0.00329
	**APOH|ENSG00000091583|protein_coding**	grey	0.010418	-0.004739	0.090699	-0.02809
	**SUCNR1|ENSG00000198829|protein_coding**	grey	0.242416	0.107411	0.264211	-0.009

	**HIST1H3B|ENSG00000274267|protein_coding**	grey	0.420343	0.152025	0.471298	-0.02979
	**CDC20|ENSG00000117399|protein_coding**	grey	0.922412	0.160581	0.930861	-0.05462
	**AQP2|ENSG00000167580|protein_coding**	grey	0.017543	0.009114	-0.00303	-0.01154
	**COL11A1|ENSG00000060718|protein_coding**	brown	0.122211	0.971073	0.150882	-0.0018
	**CLRN3|ENSG00000180745|protein_coding**	grey	0.282411	0.03142	0.321156	-0.00864
	**TYRP1|ENSG00000107165|protein_coding**	grey	0.0794	0.096144	0.094923	0.07727
	**GRP|ENSG00000134443|protein_coding**	grey	-0.05204	0.228773	-0.06143	-0.02533
	**AKR1C3|ENSG00000196139|protein_coding**	grey	0.250094	0.087398	0.323391	0.022589
	**PAH|ENSG00000171759|protein_coding**	grey	-0.10996	-0.045367	-0.14755	0.137559
	**EXO1|ENSG00000174371|protein_coding**	grey	0.921691	0.217105	0.914987	-0.04228
	**CCDC144NL|ENSG00000205212|protein_coding**	grey	0.082748	0.036299	0.102641	-0.01693
	**CHAT|ENSG00000070748|protein_coding**	grey	0.051318	-0.003903	0.090255	-0.01423
	**PTPRR|ENSG00000153233|protein_coding**	grey	0.057034	0.021078	0.094641	-0.01373
	**FBXO40|ENSG00000163833|protein_coding**	turquoise	-0.03336	0.007121	-0.04728	0.964601
	**ANKRD30A|ENSG00000148513|protein_coding**	grey	0.035843	0.140651	0.065643	-0.02079
	**OR1Q1|ENSG00000165202|protein_coding**	grey	0.195064	0.095292	0.242564	-0.00723
	**KCTD8|ENSG00000183783|protein_coding**	grey	-0.00287	-0.022726	-0.03846	-0.03206
	**DDX53|ENSG00000184735|protein_coding**	grey	0.072563	0.025007	0.133603	-0.00823
	**CSAG2|ENSG00000268902|protein_coding**	grey	0.044247	0.0083	0.088857	-0.0239
	**C9orf135|ENSG00000204711|protein_coding**	grey	0.107775	0.054969	0.147711	0.007744
	**OMP|ENSG00000254550|protein_coding**	grey	0.325076	0.01836	0.391539	-0.04466
	**SI|ENSG00000090402|protein_coding**	grey	0.1474	0.036765	0.199495	-0.02804

<table>
<tr><th></th><th></th><th></th><th></th><th></th><th></th><th></th></tr>
<tr><td></td><td>KIF20A|ENSG00000112984|protein_coding</td><td>grey</td><td>0.948616</td><td>0.191278</td><td>0.922511</td><td>-0.04745</td></tr>
<tr><td></td><td>PRDM13|ENSG00000112238|protein_coding</td><td>grey</td><td>-0.03441</td><td>-0.010887</td><td>-0.03775</td><td>-0.02796</td></tr>
<tr><td></td><td>EFNA3|ENSG00000143590|protein_coding</td><td>grey</td><td>0.150273</td><td>0.078731</td><td>0.268819</td><td>-0.03828</td></tr>
<tr><td></td><td>HTR2A|ENSG00000102468|protein_coding</td><td>grey</td><td>0.112247</td><td>0.170928</td><td>0.16355</td><td>0.044631</td></tr>
<tr><td></td><td>PSCA|ENSG00000167653|protein_coding</td><td>grey</td><td>0.059055</td><td>0.171377</td><td>0.087069</td><td>-0.01937</td></tr>
<tr><td></td><td>CTHRC1|ENSG00000164932|protein_coding</td><td>brown</td><td>0.186752</td><td>0.966083</td><td>0.233438</td><td>-0.01545</td></tr>
<tr><td></td><td>TLX2|ENSG00000115297|protein_coding</td><td>grey</td><td>0.184234</td><td>0.055686</td><td>0.221014</td><td>-0.03788</td></tr>
<tr><td></td><td>PPFIA2|ENSG00000139220|protein_coding</td><td>grey</td><td>0.124257</td><td>0.081146</td><td>0.181984</td><td>-0.04767</td></tr>
<tr><td></td><td>INSM2|ENSG00000168348|protein_coding</td><td>grey</td><td>0.119918</td><td>0.064807</td><td>0.174381</td><td>-0.00666</td></tr>
<tr><td></td><td>KIF4A|ENSG00000090889|protein_coding</td><td>blue</td><td>0.965518</td><td>0.179901</td><td>0.943374</td><td>-0.04545</td></tr>
<tr><td></td><td>LHFPL5|ENSG00000197753|protein_coding</td><td>grey</td><td>0.103252</td><td>0.04664</td><td>0.178472</td><td>0.013466</td></tr>
<tr><td></td><td>DIRAS2|ENSG00000165023|protein_coding</td><td>grey</td><td>0.227488</td><td>0.033277</td><td>0.287163</td><td>-0.0462</td></tr>
<tr><td></td><td>ACY3|ENSG00000132744|protein_coding</td><td>grey</td><td>0.00191</td><td>-0.011454</td><td>0.041902</td><td>-0.011</td></tr>
<tr><td></td><td>LCN2|ENSG00000148346|protein_coding</td><td>grey</td><td>-0.08302</td><td>-0.046866</td><td>-0.13333</td><td>0.034796</td></tr>
<tr><td></td><td>FAM135B|ENSG00000147724|protein_coding</td><td>grey</td><td>0.368854</td><td>-0.005487</td><td>0.350268</td><td>-0.0184</td></tr>
<tr><td></td><td>FGA|ENSG00000171560|protein_coding</td><td>grey</td><td>-0.0628</td><td>-0.025686</td><td>-0.0347</td><td>-0.02424</td></tr>
<tr><td></td><td>CCDC166|ENSG00000255181|protein_coding</td><td>grey</td><td>0.371234</td><td>0.016389</td><td>0.401939</td><td>-0.06392</td></tr>
<tr><td></td><td>IQGAP3|ENSG00000183856|protein_coding</td><td>grey</td><td>0.91237</td><td>0.16588</td><td>0.909725</td><td>-0.03855</td></tr>
<tr><td></td><td>PLK1|ENSG00000166851|protein_coding</td><td>grey</td><td>0.953754</td><td>0.167215</td><td>0.940635</td><td>-0.03872</td></tr>
<tr><td></td><td>FOXG1|ENSG00000176165|protein_coding</td><td>grey</td><td>0.055339</td><td>0.012625</td><td>0.073039</td><td>-0.01444</td></tr>
<tr><td></td><td>CA14|ENSG00000118298|protein_coding</td><td>grey</td><td>-0.21752</td><td>-0.105046</td><td>-0.33564</td><td>-0.02759</td></tr>
<tr><td></td><td>SLC5A8|ENSG00000256870|protein_coding</td><td>grey</td><td>-0.1358</td><td>-0.072435</td><td>-0.22584</td><td>-0.00642</td></tr>
</table>

	**CLPSL1|ENSG00000204140|protein_coding**	grey	0.109973	0.01392	0.173208	0.054152
	**ABCC11|ENSG00000121270|protein_coding**	grey	0.055681	0.00446	0.090409	-0.02034
	**SPAG11A|ENSG00000178287|protein_coding**	grey	0.01862	-0.017602	0.002366	-0.01834
	**SGK2|ENSG00000101049|protein_coding**	grey	0.231081	0.007359	0.302885	-0.02291
	**MYL1|ENSG00000168530|protein_coding**	turquoise	-0.04979	-0.002645	-0.05229	0.986103
	**DYTN|ENSG00000232125|protein_coding**	grey	0.166142	0.098615	0.249538	-0.01748
	**ASIC2|ENSG00000108684|protein_coding**	grey	0.190867	0.048219	0.261062	-0.03924
	**HIST1H3C|ENSG00000278272|protein_coding**	grey	0.179284	0.024981	0.200933	-0.02547
	**SSTR1|ENSG00000139874|protein_coding**	grey	0.526849	0.076205	0.580923	-0.03893
	**ANO3|ENSG00000134343|protein_coding**	grey	0.028071	0.013725	0.047044	-0.01241
	**SCRT1|ENSG00000261678|protein_coding**	grey	0.015591	-0.00341	0.075854	0.001402
	**DUPD1|ENSG00000188716|protein_coding**	turquoise	-0.0364	-0.000907	-0.04165	0.988356
	**SLC7A4|ENSG00000099960|protein_coding**	grey	-0.18	-0.105526	-0.25152	-0.03251
	**MCM10|ENSG00000065328|protein_coding**	grey	0.925688	0.171993	0.888073	-0.04604
	**ASZ1|ENSG00000154438|protein_coding**	grey	0.099365	0.000779	0.135885	-0.01602
	**HAND1|ENSG00000113196|protein_coding**	grey	0.109512	0.066464	0.146552	-0.00873
	**CPN1|ENSG00000120054|protein_coding**	grey	-0.01706	-0.0213	0.03401	-0.01089
	**HIST1H3F|ENSG00000277775|protein_coding**	grey	0.259859	0.15158	0.277721	-0.02472
	**PPP1R3A|ENSG00000154415|protein_coding**	turquoise	-0.05205	0.004932	-0.06239	0.890376
	**CCK|ENSG00000187094|protein_coding**	grey	-0.17102	-0.09138	-0.24273	-0.02686
	**HRG|ENSG00000113905|protein_coding**	grey	-0.05436	-0.018216	-0.02385	-0.02159
	**NUSAP1|ENSG00000137804|protein_coding**	blue	0.965284	0.18525	0.931187	-0.05283

<table>
<tr><td></td><td>LY6G6D|ENSG00000244355|protein_coding</td><td>grey</td><td>-0.05839</td><td>0.04996</td><td>-0.06784</td><td>-0.00766</td></tr>
<tr><td></td><td>SOHLH2|ENSG00000120669|protein_coding</td><td>grey</td><td>-0.07174</td><td>-0.04376</td><td>-0.10011</td><td>0.057149</td></tr>
<tr><td></td><td>EFNA2|ENSG00000099617|protein_coding</td><td>grey</td><td>0.297705</td><td>0.047143</td><td>0.397517</td><td>-0.03544</td></tr>
<tr><td></td><td>SIM1|ENSG00000112246|protein_coding</td><td>grey</td><td>0.108469</td><td>0.064873</td><td>0.137229</td><td>0.011826</td></tr>
<tr><td></td><td>PROK1|ENSG00000143125|protein_coding</td><td>grey</td><td>-0.14123</td><td>-0.046676</td><td>-0.21939</td><td>-0.00304</td></tr>
<tr><td></td><td>CALML5|ENSG00000178372|protein_coding</td><td>grey</td><td>0.050493</td><td>0.094992</td><td>0.080192</td><td>-0.00787</td></tr>
<tr><td></td><td>FOXI1|ENSG00000168269|protein_coding</td><td>grey</td><td>-0.11643</td><td>-0.053445</td><td>-0.16217</td><td>-0.02722</td></tr>
<tr><td></td><td>APOA2|ENSG00000158874|protein_coding</td><td>grey</td><td>0.044342</td><td>0.030085</td><td>0.049626</td><td>0.045525</td></tr>
<tr><td></td><td>CER1|ENSG00000147869|protein_coding</td><td>grey</td><td>0.370478</td><td>0.076035</td><td>0.349558</td><td>-0.02113</td></tr>
<tr><td></td><td>C3orf30|ENSG00000163424|protein_coding</td><td>grey</td><td>0.094023</td><td>0.021823</td><td>0.110369</td><td>-0.01961</td></tr>
<tr><td></td><td>MAB21L1|ENSG00000180660|protein_coding</td><td>grey</td><td>-0.00842</td><td>-0.006316</td><td>-0.05531</td><td>0.009831</td></tr>
<tr><td></td><td>INSYN2B|ENSG00000204767|protein_coding</td><td>grey</td><td>-0.04745</td><td>0.003937</td><td>-0.14273</td><td>-0.01779</td></tr>
<tr><td></td><td>CELSR3|ENSG00000008300|protein_coding</td><td>grey</td><td>0.316077</td><td>0.137233</td><td>0.415442</td><td>-0.0311</td></tr>
<tr><td></td><td>CDC42EP5|ENSG00000167617|protein_coding</td><td>grey</td><td>-0.2697</td><td>-0.110955</td><td>-0.33555</td><td>0.010271</td></tr>
<tr><td></td><td>TRDN|ENSG00000186439|protein_coding</td><td>turquoise</td><td>-0.04986</td><td>-0.00168</td><td>-0.05489</td><td>0.992665</td></tr>
<tr><td></td><td>SGO1|ENSG00000129810|protein_coding</td><td>blue</td><td>0.971808</td><td>0.206447</td><td>0.923022</td><td>-0.06089</td></tr>
<tr><td></td><td>UTS2B|ENSG00000188958|protein_coding</td><td>grey</td><td>0.261438</td><td>0.025834</td><td>0.329704</td><td>-0.01633</td></tr>
<tr><td></td><td>MAGEA12|ENSG00000213401|protein_coding</td><td>grey</td><td>0.20498</td><td>0.05813</td><td>0.217188</td><td>-0.01457</td></tr>
<tr><td></td><td>BUB1|ENSG00000169679|protein_coding</td><td>blue</td><td>0.971597</td><td>0.243065</td><td>0.927153</td><td>-0.04633</td></tr>
<tr><td></td><td>OTOR|ENSG00000125879|protein_coding</td><td>grey</td><td>0.041583</td><td>0.14905</td><td>0.106026</td><td>-0.01491</td></tr>
<tr><td></td><td>TMPRSS11A|ENSG00000187054|protein_coding</td><td>grey</td><td>-0.0472</td><td>-0.051439</td><td>-0.09334</td><td>-0.03736</td></tr>
<tr><td></td><td>RFX6|ENSG00000185002|protein_coding</td><td>grey</td><td>0.127606</td><td>0.007489</td><td>0.176015</td><td>-0.02779</td></tr>
</table>

	**NETO1|ENSG00000166342|protein_coding**	grey	0.063269	0.009027	0.088998	-0.0122
	**KCNQ2|ENSG00000075043|protein_coding**	grey	0.021778	-0.007025	0.088211	-0.01839
	**PADI3|ENSG00000142619|protein_coding**	grey	-0.07755	-0.031166	-0.10667	-0.0219
	**B3GNT6|ENSG00000198488|protein_coding**	grey	0.192394	0.062837	0.236679	-0.04329
	**FMOD|ENSG00000122176|protein_coding**	grey	-0.11596	-0.034102	-0.20183	0.002663
	**RAB3C|ENSG00000152932|protein_coding**	grey	0.133008	0.226846	0.167861	-0.00149
	**PON1|ENSG00000005421|protein_coding**	grey	-0.01154	-0.0417	0.019435	-0.00863
	**CKM|ENSG00000104879|protein_coding**	turquoise	-0.05396	-0.004611	-0.06049	0.995363
	**FAM72C|ENSG00000263513|protein_coding**	grey	0.732323	0.101004	0.721337	0.002783
	**IGSF1|ENSG00000147255|protein_coding**	grey	-0.17378	-0.064292	-0.27873	0.303583
	**TRIM49B|ENSG00000182053|protein_coding**	grey	0.064277	0.012001	0.092152	0.000569
	**ZNF280A|ENSG00000169548|protein_coding**	grey	0.11741	0.089801	0.175658	-0.01799
	**A1CF|ENSG00000148584|protein_coding**	grey	-0.00497	-0.017377	0.014565	-0.02926
	**MYH7|ENSG00000092054|protein_coding**	turquoise	-0.05017	-0.004509	-0.0562	0.997003
	**UGT2B15|ENSG00000196620|protein_coding**	grey	0.21821	0.224596	0.283772	-0.03699
	**HTR1F|ENSG00000179097|protein_coding**	grey	0.199939	0.391827	0.230958	-0.0309
	**ARHGDIG|ENSG00000242173|protein_coding**	grey	0.236218	0.03631	0.349217	0.030347
	**CXCL5|ENSG00000163735|protein_coding**	grey	0.001969	-0.011099	-0.05236	0.061718
	**KLHL1|ENSG00000150361|protein_coding**	grey	0.138205	-0.002723	0.139809	-0.0192
	**CHRNA2|ENSG00000120903|protein_coding**	grey	-0.11981	-0.119754	-0.20145	0.012993
	**EDN3|ENSG00000124205|protein_coding**	grey	-0.06556	0.0385	-0.09729	0.007855
	**ANKRD30B|ENSG00000180777|protein_coding**	grey	0.107205	0.065949	0.12069	-0.02388

	**PLA2G2E|ENSG00000188784|protein_coding**	grey	-0.01755	-0.013789	0.04	-0.03456
	**CHI3L2|ENSG00000064886|protein_coding**	grey	-0.03317	-0.007843	-0.08577	0.034558
	**SMCO1|ENSG00000214097|protein_coding**	turquoise	-0.051	-0.005388	-0.05354	0.985038
	**FDCSP|ENSG00000181617|protein_coding**	grey	0.137198	0.164692	0.142938	-0.02122
	**KLHL40|ENSG00000157119|protein_coding**	turquoise	-0.04578	0.001243	-0.05365	0.996874
	**SLC39A2|ENSG00000165794|protein_coding**	grey	-0.0141	-0.026438	-0.04378	-0.02523
	**CDH18|ENSG00000145526|protein_coding**	grey	-0.10668	-0.059146	-0.16379	0.006527
	**UNC45B|ENSG00000141161|protein_coding**	turquoise	-0.04179	0.005775	-0.05211	0.98597
	**KRT76|ENSG00000185069|protein_coding**	grey	-0.07026	-0.026752	-0.07193	0.036877
	**BIRC5|ENSG00000089685|protein_coding**	grey	0.899967	0.157362	0.903207	-0.05135
	**DLGAP5|ENSG00000126787|protein_coding**	blue	0.980176	0.196004	0.932488	-0.04627
	**CDC20B|ENSG00000164287|protein_coding**	grey	0.013553	-0.008549	0.038055	-0.00896
	**KHDC1L|ENSG00000256980|protein_coding**	grey	0.084982	0.061796	0.108796	-0.00762
	**ORM1|ENSG00000229314|protein_coding**	grey	0.011727	-0.020802	0.006656	-0.01444
	**LTF|ENSG00000012223|protein_coding**	grey	-0.01338	-0.012683	-0.07202	-0.00296
	**FAM72B|ENSG00000188610|protein_coding**	grey	0.86937	0.167508	0.852518	-0.02956
	**LHX9|ENSG00000143355|protein_coding**	grey	0.0202	0.021571	0.065115	-0.01939
	**LRTM2|ENSG00000166159|protein_coding**	grey	0.025626	0.037052	0.127148	-0.02612
	**NEIL3|ENSG00000109674|protein_coding**	grey	0.932933	0.144973	0.899398	-0.05732
	**ZPLD1|ENSG00000170044|protein_coding**	grey	0.335527	0.209674	0.419761	-0.01763
	**OR1J1|ENSG00000136834|protein_coding**	grey	0.293381	0.142175	0.350451	0.013162
	**COL6A5|ENSG00000172752|protein_coding**	grey	-0.07685	-0.015731	-0.11194	-0.01073

	**ZNF556|ENSG00000172000|protein_coding**	grey	0.312624	0.048207	0.362386	0.014497
	**THBS2|ENSG00000186340|protein_coding**	brown	0.264724	0.954298	0.282292	0.014461
	**SPRR2F|ENSG00000244094|protein_coding**	grey	0.153776	0.157465	0.175368	-0.03228
	**CAMKV|ENSG00000164076|protein_coding**	grey	0.094345	0.025426	0.132722	0.009257
	**AKR1D1|ENSG00000122787|protein_coding**	grey	0.197722	0.075705	0.255107	-0.01904
	**ARSF|ENSG00000062096|protein_coding**	grey	-0.08514	0.137723	-0.13573	-0.03701
	**C10orf71|ENSG00000177354|protein_coding**	turquoise	-0.037	0.003269	-0.04743	0.993067
	**SLC9A3|ENSG00000066230|protein_coding**	grey	0.104376	0.029087	0.114921	-0.03628
	**CLCA2|ENSG00000137975|protein_coding**	grey	-0.06238	-0.011185	-0.10796	-0.02299
	**EOMES|ENSG00000163508|protein_coding**	grey	0.180161	0.059181	0.205574	-0.02532
	**S100P|ENSG00000163993|protein_coding**	grey	0.085094	0.023934	0.084922	0.035721
	**ANKRD34B|ENSG00000189127|protein_coding**	grey	0.249815	0.075505	0.343143	-0.04837
	**ATP6V0A4|ENSG00000105929|protein_coding**	grey	-0.09356	-0.044659	-0.14868	-0.02528
	**CA1|ENSG00000133742|protein_coding**	grey	0.126813	0.250312	0.203538	-0.00647
	**NCAPG|ENSG00000109805|protein_coding**	blue	0.94377	0.176833	0.87706	-0.04766
	**DQX1|ENSG00000144045|protein_coding**	grey	0.355501	0.018007	0.342886	-0.02028
	**IBSP|ENSG00000029559|protein_coding**	grey	0.345592	0.505695	0.389246	0.012788
	**AL049839.2|ENSG00000273259|protein_coding**	grey	-0.05466	-0.019448	-0.09762	0.057762
	**LMOD2|ENSG00000170807|protein_coding**	turquoise	-0.04407	-0.00071	-0.0492	0.992093
	**ATP12A|ENSG00000075673|protein_coding**	grey	0.008746	0.018283	0.091976	-0.01868
	**HIST1H3I|ENSG00000275379|protein_coding**	grey	0.013563	-0.000123	0.043233	-0.01148
	**CST5|ENSG00000170367|protein_coding**	grey	0.021015	0.047707	0.062486	-0.01529

	**TUBB3|ENSG00000258947|protein_coding**	grey	0.329749	0.19566	0.436921	-0.051
	**CPNE6|ENSG00000100884|protein_coding**	grey	-0.17584	-0.080271	-0.2627	-0.00012
	**CCDC194|ENSG00000269720|protein_coding**	grey	0.349677	0.046018	0.422258	-0.01492
	**MYOZ2|ENSG00000172399|protein_coding**	turquoise	-0.04707	0.000478	-0.05478	0.992231
	**E2F8|ENSG00000129173|protein_coding**	grey	0.821285	0.24732	0.802781	0.303977
	**MTFR2|ENSG00000146410|protein_coding**	grey	0.893281	0.18905	0.897605	-0.0395
	**TROAP|ENSG00000135451|protein_coding**	grey	0.891478	0.196221	0.919151	-0.0442
	**UGT1A1|ENSG00000241635|protein_coding**	grey	0.23564	0.045761	0.291395	-0.01601
