# Supplementary material for: An Integrative Multi-Omics Analysis Based on Nomogram for Predicting Prostate Cancer Bone Metastasis Incidence
Source: Genet Res (Camb). 2022 Sep 29;2022:8213723. doi: 10.1155/2022/8213723 (PMC9537037; doi:10.1155/2022/8213723)
Supplement: Supplementary Materials — Figure S1: associated with Figure 2. Figure S2: associated with Figure 5. Figure S3: associated with Figure 6. Data 1-1: SEER data baseline characteristics of patient with prostate cancer; Data 1-2: train group ROC and validation ROC, Delong's test; Data 1-3: clinical features of TCGA samples and predictive results. Data 2: TMB scores of TCGA samples. Data 3: genes with different frequencies of CNVs between LRBM and HRBM cohorts. Data 4-1: DElncRNAs; Data 4-2: DEmiRNAs; Data 4-3: DEmRNAs. Data 5: gene module. Data 6-1: 29 immune microenvironment signatures; Data-2: ssGSEA score of immune microenvironment signature; Data 6-3: stromal score, immune score, estimate score, and tumor purity of TCGA samples. [file 8213723.f1.zip › 8213723.f1/supplementary data6 (1).pdf]

Supplementary Data 6-1：29 immune microenvironment sginatures

signature

aDCs：CD83 LAMP3 CCL1

APC_co_inhibition： C10orf54 CD274 LGALS9 PDCD1LG2 PVRL3

APC_co_stimulation：CD40 CD58 CD70 ICOSLG SLAMF1 TNFSF14 TNFSF15 TNFSF18 TNFSF4 TNFSF8 TNFSF9

B_cells：BACH2 BANK1 BLK BTLA CD79A CD79B FCRL1 FCRL3 HVCN1 RALGPS2

CCR：CCL16 TPO TGFBR2 CXCL2 CCL14 TGFBR3 IL11RA CCL11 IL4I1 IL33 CXCL12 CXCL10 BMPER BMP8A CXCL11 IL21R IL17B TNFRSF9 ILF2 CX3CR1 CCR8 TNFSF12 CSF3 TNFSF4 BMP3 CX3CL1 BMP5 CXCR2 TNFRSF10D BMP2 CXCL14 CCL28 CXCL3 BMP6 CCL21 CXCL9 CCL23 IL6 TNFRSF18 IL17RD IL17D IL27 CCL7 IL1R1 CXCR4 CXCR2P1 TGFB1I1 IFNGR1 IL9R IL1RAPL1 IL11 CSF1 IL20RA IL25 TNFRSF4 IL18 ILF3 CCL20 TNFRSF12A IL6ST CXCL13 IL12B TNFRSF8 IL6R BMPR2 IFNE IL1RAPL2 IL3RA BMP4 CCL24 TNFSF13B CCR4 IL2RA IL32 TNFRSF10C IL22RA1 BMPR1A CXCR5 CXCR3 IFNA8 IL17REL IFNB1 IFNAR1 TNFRSF1B CCL17 IFNL1 IL16 IL1RL1 ILK CCL25 ILDR2 CXCR1 IL36RN IL34 TGFB1 IFNG IL19 ILKAP BMP2K CCR10 ILDR1 EPO CCR7 IL17C IL23A CCR5 IL7 EPOR CCL13 IL2RG IL31RA TNFAIP6 IFNL2 BMP1 IL12RB1 TNFAIP8 IL4R TNFRSF6B TNFAIP8L1 TNFRSF10B IFNL3 CCL5 CXCL6 CXCL1 CCR3 TNFSF11 CSF1R IL21 IL1RAP IL12RB2 CCL1 IL17RA CCR1 IL1RN TNFRSF11B TNFRSF14 IL13 IL2RB BMP8B CCL2 IL24 IL18RAP TGFBI TNFSF10 TNFRSF11A CXCL5 IL5RA TNFSF9 IL1RL2 TNFRSF13C IL36G IL15RA TNFRSF21 CXCL8 IL22RA2 TNFAIP8L2 IL18R1 IFNLR1 CXCR6 CCL3L3 TNFRSF1A IL17RE IFNGR2 IL17RC TNFAIP8L3 ILVBL TGFBRAP1 CCL4L1 CSF2RA CCRN4L CCL26 TNFAIP1 CCRL2 IFNA10 TNFRSF17 IFNA13 IL20 IL18BP CCL3L1 TNFSF12-TNFSF13 IL5 IL23R IL26 TNF TGFA CSF2 IL1F10 CXCL17 TNFSF13 IFNA4 IL37 IL12A IL7R IFNA1 IL1A IL4 IL2 CCL22 CSF3R IL10 IFNK TGFB2 IL1R2 IL1B IL17F IL27RA IL15 TNFSF8 IL36B XCL1 CXCL16 TNFRSF19 IL3 CCL3 IFNA2 BMPR1B IFNA21 TNFSF18 CCL8 IL17RB TNFRSF25 IL22 IL10RB IFNAR2 CCL18 IFNA16 CSF2RB IL36A TNFAIP3 IL13RA2 IL13RA1 CCR9 TNFRSF10A IFNA7 IFNW1 XCL2 TNFSF14 CCR2 BMP15 BMP10 CCL15-CCL14 TGFBR1 IFNA5 BMP7 IFNA14 IL20RB IL10RA IFNA17 CCR6 TGFB3 CCL15 CCL4 CCL27 TNFRSF13B TNFAIP2 IL31 IL17A TNFSF15 CCL19 IFNA6 IL9

CD8+_T_cells：CD8A

Check-point：IDO1 LAG3 CTLA4 TNFRSF9 ICOS CD80 PDCD1LG2 TIGIT CD70 TNFSF9 ICOSLG KIR3DL1 CD86 PDCD1 LAIR1 TNFRSF8 TNFSF15 TNFRSF14 IDO2 CD276 CD40 TNFRSF4 TNFSF14 HHLA2 CD244 CD274 HAVCR2 CD27 BTLA LGALS9 TMIGD2 CD28 CD48 TNFRSF25 CD40LG ADORA2A VTCN1 CD160

CD44 TNFSF18 TNFRSF18 BTNL2 C10orf54 CD200R1 TNFSF4 CD200 NRP1

Cytolytic_activity：PRF1 GZMA

DCs：CCL17 CCL22 CD209 CCL13

HLA：HLA-E HLA-DPB2 HLA-C HLA-J HLA-DQB1 HLA-DQB2 HLA-DQA2 HLA-DQA1 HLA-A HLA-DMA HLA-DOB HLA-DRB1 HLA-H HLA-B HLA-DRB5 HLA-DOA HLA-DPB1 HLA-DRA HLA-DRB6 HLA-L HLA-F HLA-G HLA-DMB HLA-DPA1

iDCs：CD1A CD1E

Inflammation-promoting：CCL5 CD19 CD8B CXCL10 CXCL13 CXCL9 GNLY GZMB IFNG IL12A IL12B IRF1 PRF1 STAT1 TBX21

Macrophages：C11orf45 CD68 CLEC5A CYBB FUCA1 GPNMB HS3ST2 LGMN MMP9 TM4SF19

Mast_cells：CMA1 MS4A2 TPSAB1

MHC_class_I：B2M HLA-A TAP1

Neutrophils：EVI2B HSD17B11 KDM6B MEGF9 MNDA NLRP12 PADI4 SELL TRANK1 VNN3

NK_cells：KLRC1 KLRF1

Parainflammation：CXCL10 PLAT CCND1 LGMN PLAUR AIM2 MMP7 ICAM1 MX2 CXCL9 ANXA1 TLR2 PLA2G2D ITGA2 MX1 HMOX1 CD276 TIRAP IL33 PTGES TNFRSF12A SCARB1 CD14 BLNK IFIT3 RETNLB IFIT2 ISG15 OAS2 REL OAS3 CD44 PPARG BST2 OAS1 NOX1 PLA2G2A IFIT1 IFITM3 IL1RN

pDCs：CLEC4C CXCR3 GZMB IL3RA IRF7 IRF8 LILRA4 PHEX PLD4 PTCRA

T_cell_co-inhibition：BTLA C10orf54 CD160 CD244 CD274 CTLA4 HAVCR2 LAG3 LAIR1 TIGIT

T_cell_co-stimulation：CD2 CD226 CD27 CD28 CD40LG ICOS SLAMF1 TNFRSF18 TNFRSF25 TNFRSF4 TNFRSF8 TNFRSF9 TNFSF14

T_helper_cells：CD4

Tfh：PDCD1 CXCL13 CXCR5

Th1_cells：IFNG TBX21 CTLA4 STAT4 CD38 IL12RB2 LTA CSF2

Th2_cells：PMCH LAIR2 SMAD2 CXCR6 GATA3 IL26

TIL：ITM2C CD38 THEMIS2 GLYR1 ICOS F5 TIGIT KLRD1 IRF4 PRKCQ FCRL5 SIRPG LPXN IL2RG CCL5 LCK TRAF3IP3 CD86 MAL LILRB1 DOK2 CD6 PAG1 LAX1 PLEK PIK3CD SLAMF1 XCL1 GPR171 XCL2 TBX21 CD2 CD53 KLHL6 SLAMF6 CD40 SIT1 TNFRSF4 CD79A CD247 LCP2 CD3D CD27 SH2D1A FYB ARHGAP30 ACAP1 CST7 CD3G IL2RB CD3E FCRL3 CORO1A ITK TCL1A CYBB CSF2RB IKZF1 NCF4 DOCK2 CCR2 PTPRC PLAC8 NCKAP1L IL7R 6-Sep CD28 STAT4 CD8A LY9 CD48 HCST PTPRCAP SASH3 ARHGAP25 LAT TRAT1 IL10RA PAX5 CCR7 DOCK11 PARVG SPNS1 CD52 HCLS1 ARHGAP9 GIMAP6 PRKCB MS4A1 GPR18 TBC1D10C GVINP1 P2RY8 EVI2B VAMP5 KLRK1 SELL MPEG1

MS4A6A ARHGAP15 MFNG GZMK SELPLG TARP GIMAP7 FAM65B INPP5D ITGA4 MZB1 GPSM3 STK10 CLEC2D IL16 NLRC3 GIMAP5 GIMAP4 IFFO1 CFH PVRIG CFHR1

Treg：IL12RB2 TMPRSS6 CTSC LAPTM4B TFRC RNF145 NETO2 ADAT2 CHST2 CTLA4 NFE2L3 LIMA1 IL1R2 ICOS HSDL2 HTATIP2 FKBP1A TIGIT CCR8 LTA SLC35F2 IL21R AHCYL1 SOCS2 ETV7 BCL2L1 RRAGB ACSL4 CHRNA6 BATF LAX1 ADPRH TNFRSF4 ANKRD10 CD274 CASP1 LY75 NPTN SSTR3 GRSF1 CSF2RB TMEM184C NDFIP2 ZBTB38 ERI1 TRAF3 NAB1 HS3ST3B1 LAYN JAK1 VDR LEPROT GCNT1 PTPRJ IKZF2 CSF1 ENTPD1 TNFRSF18 METTL7A KSR1 SSH1 CADM1 IL1R1 ACP5 CHST7 THADA CD177 NFAT5 ZNF282 MAGEH1

Type_I_IFN_Reponse：DDX4 IFIT1 IFIT2 IFIT3 IRF7 ISG20 MX1 MX2 RSAD2 TNFSF10

Type_II_IFN_Reponse：GPR146 SELP AHR

Supplementary Data 6-2:stromal score, immune score, estimate score, tumor purtiy of TCGA samples

	ID	StromalScore	ImmuneScore	ESTIMATEScore	TumorPurity
	**TCGA-KK-A6E5**	-550.919	-252.873	-803.793	0.883741
	**TCGA-EJ-A65E**	-1180.92	-694.705	-1875.62	0.94615
	**TCGA-EJ-7785**	-55.1382	197.5854	142.4472	0.810438
	**TCGA-SU-A7E7**	-586.069	91.52555	-494.543	0.861594
	**TCGA-VN-A88Q**	-1194.96	-646.745	-1841.7	0.944526
	**TCGA-EJ-8472**	-727.43	-794.506	-1521.94	0.928076
	**TCGA-HC-8262**	-1083.66	-736.556	-1820.22	0.943486
	**TCGA-HC-7747**	-518.929	402.9644	-115.964	0.832071
	**TCGA-QU-A6IM**	-839.45	558.0319	-281.418	0.845296
	**TCGA-HC-7819**	-1349.71	-930.08	-2279.79	0.96368
	**TCGA-EJ-5510**	-513.076	69.56141	-443.514	0.857768
	**TCGA-HC-A76X**	-1318.14	-1159.26	-2477.4	0.97102
	**TCGA-J4-A67N**	-867.235	185.5835	-681.652	0.875209
	**TCGA-EJ-A7NH**	-647.578	-536.732	-1184.31	0.908488
	**TCGA-G9-6365**	5.6383	-228.961	-223.323	0.840708
	**TCGA-XA-A8JR**	-593.903	-386.188	-980.09	0.895554
	**TCGA-KC-A4BL**	-436.235	71.14078	-365.094	0.851794
	**TCGA-CH-5737**	-1201.39	-726.694	-1928.09	0.948615
	**TCGA-XK-AAK1**	-1037.84	-346.009	-1383.85	0.920337
	**TCGA-XK-AAIR**	-482.949	-1.34205	-484.291	0.860829
	**TCGA-HC-7210**	155.8637	247.7469	403.6107	0.78739
	**TCGA-G9-6361**	-191.049	-624.053	-815.102	0.884517
	**TCGA-YL-A8HO**	430.1321	994.4227	1424.555	0.686526
	**TCGA-CH-5765**	-1080.24	-954.542	-2034.78	0.953454

	**TCGA-EJ-A65G**	-1039.41	-891.546	-1930.96	0.948748
	**TCGA-HC-7742**	-429.711	-673.735	-1103.45	0.903464
	**TCGA-CH-5763**	-64.211	-120.983	-185.194	0.837665
	**TCGA-J4-A6G3**	-375.692	716.244	340.5515	0.793062
	**TCGA-HC-8216**	-481.47	165.0773	-316.393	0.848027
	**TCGA-2A-A8W1**	-1670.59	-884.165	-2554.76	0.973671
	**TCGA-G9-6362**	-567.704	-343.178	-910.881	0.890988
	**TCGA-EJ-7782**	-483.682	-358.448	-842.13	0.88636
	**TCGA-CH-5743**	-688.072	794.6426	106.5705	0.813512
	**TCGA-EJ-5501**	-877.725	-482.554	-1360.28	0.918979
	**TCGA-EJ-A7NG**	-433.525	-324.051	-757.576	0.880546
	**TCGA-VP-A876**	-1328.38	-967.589	-2295.97	0.964311
	**TCGA-J4-A83L**	-503.126	-602.716	-1105.84	0.903614
	**TCGA-VN-A943**	-1925.26	-1339.23	-3264.49	0.992098
	**TCGA-YL-A8SH**	-666.68	-474.493	-1141.17	0.905824
	**TCGA-EJ-7315**	210.2827	922.3812	1132.664	0.717041
	**TCGA-EJ-5508**	-405.102	298.4548	-106.648	0.831312
	**TCGA-KK-A7AV**	-282.044	1065.023	782.9797	0.751859
	**TCGA-V1-A8MU**	1.836886	455.7875	457.6244	0.782478
	**TCGA-QU-A6IL**	-1059.14	-225.058	-1284.2	0.914518
	**TCGA-J4-A67O**	-112.713	161.6042	48.89076	0.818407
	**TCGA-EJ-7781**	-529.027	-56.4493	-585.477	0.868293
	**TCGA-G9-6348**	-124.904	66.18476	-58.7193	0.827381
	**TCGA-XK-AAJU**	-884.257	-149.079	-1033.34	0.899004
	**TCGA-J4-A83J**	-887.83	139.9681	-747.862	0.879869
	**TCGA-WW-A8ZI**	-936.194	-405.62	-1341.81	0.917907
	**TCGA-EJ-5503**	-472.702	32.4511	-440.25	0.857522
	**TCGA-HC-7211**	-915.959	-92.594	-1008.55	0.897405
	**TCGA-CH-5750**	-777.322	358.3619	-418.96	0.85591
	**TCGA-HI-7170**	253.5063	346.1311	599.6374	0.769328
	**TCGA-KK-A6E3**	-176.616	580.5962	403.9799	0.787357
	**TCGA-CH-5746**	-1240.23	-902.968	-2143.19	0.958132
	**TCGA-EJ-7123**	-817.021	-687.724	-1504.74	0.927133
	**TCGA-J4-A67L**	-471.71	499.0426	27.33273	0.820221
	**TCGA-EJ-A7NJ**	-1051.78	-107.68	-1159.46	0.906958
	**TCGA-KC-A7F6**	-842.022	-382.215	-1224.24	0.910922
	**TCGA-V1-A9ZR**	272.7534	707.4098	980.1633	0.732463
	**TCGA-KK-A6DY**	-808.604	-17.5541	-826.158	0.885272
	**TCGA-4L-AA1F**	1771.63	1646.975	3418.604	0.447527
	**TCGA-XK-AAJT**	-100.447	15.52334	-84.9236	0.829535
	**TCGA-HC-8260**	-579.914	-350.13	-930.045	0.892261
	**TCGA-V1-A8MK**	-622.472	-405.778	-1028.25	0.898677
	**TCGA-HI-7169**	-221.315	-436.674	-657.989	0.873523

	**TCGA-KC-A4BN**	-1475.86	-742.934	-2218.79	0.96125
	**TCGA-V1-A8ML**	-387.152	-653.661	-1040.81	0.899485
	**TCGA-EJ-A7NK**	-843.467	-534.119	-1377.59	0.919977
	**TCGA-G9-6384**	-342.08	-240.536	-582.616	0.868084
	**TCGA-J4-A83I**	-525.209	-199.495	-724.704	0.878249
	**TCGA-VP-A87C**	514.4221	2018.131	2532.553	0.55973
	**TCGA-EJ-A65M**	-566.609	102.4297	-464.179	0.859323
	**TCGA-EJ-7797**	-924.779	-279.896	-1204.67	0.909733
	**TCGA-EJ-AB27**	-938.185	283.6279	-654.557	0.873278
	**TCGA-KK-A8I6**	-1001.77	-455.576	-1457.35	0.924504
	**TCGA-2A-A8VV**	-922.085	-996.459	-1918.54	0.948171
	**TCGA-VN-A88I**	670.0433	333.7929	1003.836	0.730093
	**TCGA-2A-A8VX**	-1072.42	-1325.51	-2397.93	0.968166
	**TCGA-EJ-A8FN**	-417.349	131.6857	-285.663	0.845628
	**TCGA-G9-6379**	-304.06	1045.94	741.8798	0.755823
	**TCGA-EJ-7788**	-111.931	-291.294	-403.225	0.854713
	**TCGA-HC-7740**	-189.717	1.474221	-188.243	0.837909
	**TCGA-KC-A7F3**	-914.035	-428.331	-1342.37	0.917939
	**TCGA-EJ-7792**	397.2586	586.6029	983.8614	0.732093
	**TCGA-EJ-7317**	-742.128	-884.632	-1626.76	0.933696
	**TCGA-EJ-5498**	152.096	180.6568	332.7528	0.793759
	**TCGA-G9-6369**	-747.353	-778.112	-1525.46	0.928268
	**TCGA-QU-A6IO**	-886.222	257.8784	-628.343	0.871397
	**TCGA-EJ-7125**	-407.43	-154.75	-562.18	0.866591
	**TCGA-J4-A6G1**	104.5695	1996.209	2100.779	0.611092
	**TCGA-KK-A6E4**	-1099.23	-370.478	-1469.71	0.925194
	**TCGA-EJ-7314**	-1273.26	-696.916	-1970.18	0.950552
	**TCGA-YL-A8SA**	-1408.14	-812.249	-2220.39	0.961315
	**TCGA-EJ-7330**	-317.347	-430.128	-747.475	0.879842
	**TCGA-EJ-7325**	-867.187	-491.675	-1358.86	0.918897
	**TCGA-EJ-7791**	225.2806	-82.7696	142.511	0.810433
	**TCGA-VN-A88R**	-334.065	-328.302	-662.367	0.873836
	**TCGA-XK-AAJP**	-682.404	-268.546	-950.951	0.893643
	**TCGA-2A-A8VL**	-1095.46	-795.652	-1891.11	0.946883
	**TCGA-KK-A8IG**	173.8688	-95.9687	77.90019	0.815952
	**TCGA-QU-A6IN**	-1388.39	-814.06	-2202.45	0.960586
	**TCGA-EJ-8468**	164.2736	345.3649	509.6384	0.777701
	**TCGA-G9-7510**	-98.1399	-54.8702	-153.01	0.835075
	**TCGA-EJ-5522**	-439.508	-24.1621	-463.67	0.859285
	**TCGA-EJ-A46H**	86.66863	415.293	501.9616	0.778409
	**TCGA-HC-7230**	-1262.98	-893.243	-2156.23	0.958678
	**TCGA-KK-A8IF**	-950.925	-937.141	-1888.07	0.94674
	**TCGA-HC-7737**	-10.6869	510.894	500.2071	0.77857

	**TCGA-EJ-8474**	-253.088	-458.511	-711.599	0.877327
	**TCGA-G9-6370**	-324.237	-126.386	-450.623	0.858304
	**TCGA-HC-7744**	-1095.9	-790.393	-1886.29	0.946656
	**TCGA-EJ-5527**	43.44897	129.4965	172.9455	0.807807
	**TCGA-EJ-5509**	-180.462	390.5378	210.0759	0.804583
	**TCGA-TP-A8TV**	-1128.93	-899.914	-2028.84	0.953191
	**TCGA-G9-6364**	-123.666	-530.704	-654.37	0.873265
	**TCGA-G9-A9S7**	-1360.39	-495.443	-1855.83	0.945206
	**TCGA-CH-5767**	-915.409	-841.629	-1757.04	0.940372
	**TCGA-EJ-5502**	-391.004	230.4506	-160.554	0.835684
	**TCGA-V1-A8WL**	-424.239	295.8255	-128.413	0.833083
	**TCGA-CH-5791**	-597.718	-313.266	-910.984	0.890995
	**TCGA-FC-A66V**	-858.991	-36.6675	-895.658	0.889971
	**TCGA-EJ-5497**	-500.087	-234.355	-734.442	0.878931
	**TCGA-HC-7078**	-528.631	-14.8158	-543.447	0.865216
	**TCGA-KK-A7AQ**	-491.99	-334.082	-826.072	0.885267
	**TCGA-G9-6332**	-1316.66	-930.309	-2246.97	0.962382
	**TCGA-CH-5740**	-854.275	-264.437	-1118.71	0.904422
	**TCGA-G9-6338**	-205.782	-440.907	-646.689	0.872715
	**TCGA-EJ-5496**	-1266.12	-791.693	-2057.82	0.954468
	**TCGA-KK-A59Z**	165.8213	37.56932	203.3906	0.805165
	**TCGA-J4-A83M**	-835.576	-285.64	-1121.22	0.904579
	**TCGA-XK-AAJR**	-513.191	-552.966	-1066.16	0.901104
	**TCGA-KK-A6E2**	-1168.55	-532.419	-1700.97	0.93754
	**TCGA-VN-A88L**	-326.818	-684.691	-1011.51	0.897597
	**TCGA-G9-7522**	-404.052	-253.725	-657.777	0.873508
	**TCGA-EJ-5516**	238.4179	187.9474	426.3653	0.785327
	**TCGA-G9-6363**	-478.376	-169.923	-648.299	0.87283
	**TCGA-QU-A6IP**	-827.574	-577.998	-1405.57	0.92158
	**TCGA-J4-A67R**	-470.042	522.2483	52.20659	0.818127
	**TCGA-EJ-7784**	-996.316	-572.426	-1568.74	0.930612
	**TCGA-CH-5794**	-1123.11	-590.107	-1713.22	0.938165
	**TCGA-HC-A8D1**	-247.154	-33.856	-281.01	0.845264
	**TCGA-HC-A76W**	-27.798	-121.011	-148.809	0.834736
	**TCGA-KK-A8I5**	-489.28	-546.721	-1036	0.899176
	**TCGA-J4-AAU2**	-798.852	-434.392	-1233.24	0.911467
	**TCGA-CH-5739**	-35.177	80.75691	45.57989	0.818686
	**TCGA-CH-5762**	-308.602	304.3937	-4.20853	0.822861
	**TCGA-CH-5752**	-506.991	-784.822	-1291.81	0.914969
	**TCGA-HC-A8D0**	-264.762	205.2909	-59.4714	0.827443
	**TCGA-EJ-5515**	-673.702	-643.194	-1316.9	0.916449
	**TCGA-J4-8200**	-330.938	-433.097	-764.035	0.880995
	**TCGA-EJ-5517**	-358.117	102.5553	-255.562	0.843262

	**TCGA-TP-A8TT**	-164.259	878.0789	713.8199	0.758513
	**TCGA-VP-A87E**	42.31693	866.7521	909.069	0.739528
	**TCGA-EJ-7331**	29.88053	14.69873	44.57926	0.81877
	**TCGA-G9-6339**	-968.153	-412.129	-1380.28	0.920132
	**TCGA-EJ-5505**	-1129.83	-670.282	-1800.12	0.942504
	**TCGA-CH-5768**	-1110.67	-619.797	-1730.47	0.939038
	**TCGA-CH-5789**	-562.14	-32.7285	-594.868	0.868976
	**TCGA-VN-A88P**	-233.898	-254.898	-488.796	0.861166
	**TCGA-EJ-7794**	-227.951	96.94341	-131.008	0.833294
	**TCGA-Y6-A8TL**	-651.237	-664.689	-1315.93	0.916392
	**TCGA-EJ-5511**	-518.191	-752.631	-1270.82	0.913722
	**TCGA-KC-A7FE**	29.0553	-227.909	-198.853	0.838758
	**TCGA-EJ-5499**	-589.764	147.3465	-442.417	0.857685
	**TCGA-EJ-A8FS**	-1426.04	-775.629	-2201.67	0.960554
	**TCGA-J4-A67M**	-93.548	753.3234	659.7754	0.763659
	**TCGA-CH-5744**	-868.189	-249.409	-1117.6	0.904352
	**TCGA-XK-AAJA**	-1137.96	-642.598	-1780.56	0.941541
	**TCGA-HC-8258**	-924.061	-157.559	-1081.62	0.902086
	**TCGA-EJ-7783**	-534.363	-239.466	-773.829	0.881674
	**TCGA-G9-7525**	-285.851	-461.831	-747.682	0.879857
	**TCGA-EJ-A7NF**	-1449.35	-742.86	-2192.21	0.960167
	**TCGA-EJ-A46I**	-873.349	-578.281	-1451.63	0.924183
	**TCGA-J4-A67S**	-1042.32	34.03379	-1008.29	0.897388
	**TCGA-J4-AATV**	-414.197	221.2137	-192.983	0.838289
	**TCGA-EJ-7786**	-463.729	-531.096	-994.825	0.896514
	**TCGA-KC-A7FA**	-681.824	-218.643	-900.467	0.890293
	**TCGA-EJ-7115**	-317.455	-468.905	-786.36	0.88254
	**TCGA-VP-A875**	-1328.58	-1189.14	-2517.72	0.972418
	**TCGA-EJ-5542**	-243.566	-427.758	-671.324	0.874475
	**TCGA-KK-A8I9**	-63.6453	409.6281	345.9828	0.792577
	**TCGA-CH-5745**	-248.241	294.5147	46.2735	0.818627
	**TCGA-G9-6377**	-660.693	-102.755	-763.448	0.880954
	**TCGA-KK-A7B1**	-130.15	-405.108	-535.258	0.864613
	**TCGA-XK-AAJ3**	-475.806	-715.781	-1191.59	0.908934
	**TCGA-VP-A87J**	-864.736	-740.693	-1605.43	0.93257
	**TCGA-CH-5790**	-1077.92	-421.609	-1499.53	0.926846
	**TCGA-VN-A88O**	-890.906	-182.484	-1073.39	0.901564
	**TCGA-V1-A9OX**	-673.83	65.78081	-608.049	0.869932
	**TCGA-HC-7212**	-541.107	-640.05	-1181.16	0.908295
	**TCGA-EJ-5531**	-307.639	411.2104	103.5716	0.813768
	**TCGA-V1-A8X3**	-457.34	-464.052	-921.392	0.891687
	**TCGA-HC-8265**	-722.799	-173.087	-895.886	0.889986
	**TCGA-EJ-5512**	-893.804	-673.594	-1567.4	0.93054

	**TCGA-V1-A8MF**	-243.85	509.9005	266.0503	0.799676
	**TCGA-G9-6333**	-455.719	-191.257	-646.975	0.872735
	**TCGA-EJ-7327**	63.62152	-321.117	-257.496	0.843414
	**TCGA-EJ-7218**	-971.767	-469.078	-1440.84	0.923577
	**TCGA-KK-A8IH**	-929.22	-23.0246	-952.245	0.893728
	**TCGA-KK-A8IM**	-416.234	-82.4621	-498.696	0.861904
	**TCGA-KK-A7AY**	-894.268	-422.359	-1316.63	0.916433
	**TCGA-G9-6496**	-387.881	-520.066	-907.947	0.890792
	**TCGA-KC-A7FD**	-1526.01	-722.66	-2248.67	0.96245
	**TCGA-HC-7748**	12.49442	13.38872	25.88314	0.820343
	**TCGA-EJ-5532**	-488.733	-589.556	-1078.29	0.901875
	**TCGA-HC-7752**	-765.134	-364.914	-1130.05	0.905131
	**TCGA-CH-5771**	303.745	614.0178	917.7628	0.738668
	**TCGA-EJ-7312**	-890.892	-524.03	-1414.92	0.922112
	**TCGA-FC-A8O0**	-973.133	-915.275	-1888.41	0.946756
	**TCGA-EJ-7321**	-907.166	-3.32055	-910.487	0.890961
	**TCGA-HC-7080**	-1168.71	-738.124	-1906.83	0.947623
	**TCGA-G9-6494**	-512.895	-700.909	-1213.8	0.910289
	**TCGA-EJ-5530**	-473.18	-684.254	-1157.43	0.906832
	**TCGA-2A-AAYU**	-884.522	-930.915	-1815.44	0.943253
	**TCGA-V1-A8MM**	-103.711	305.6675	201.9562	0.80529
	**TCGA-HC-7079**	609.7865	3013.203	3622.99	0.4205
	**TCGA-EJ-AB20**	363.9109	509.7001	873.611	0.743021
	**TCGA-HC-7818**	-678.046	-48.3999	-726.446	0.878371
	**TCGA-VP-A87B**	-670.511	-523.647	-1194.16	0.909091
	**TCGA-V1-A9ZK**	-538.006	-453.305	-991.31	0.896286
	**TCGA-HC-8257**	-610.551	-325.494	-936.044	0.892659
	**TCGA-HC-7209**	-796.057	-358.964	-1155.02	0.906683
	**TCGA-V1-A9O9**	-274.946	1224.201	949.2552	0.735544
	**TCGA-EJ-A46B**	-951.24	-834.374	-1785.61	0.94179
	**TCGA-VN-A88N**	-1107.56	-743.424	-1850.98	0.944973
	**TCGA-HC-7749**	-343.466	-229.022	-572.488	0.867345
	**TCGA-CH-5764**	-708.846	-213.772	-922.618	0.891769
	**TCGA-EJ-5521**	-643.83	-173.828	-817.659	0.884692
	**TCGA-KC-A7F5**	-1393.48	-1323.73	-2717.21	0.97883
	**TCGA-HC-7736**	-328.308	-66.1508	-394.459	0.854044
	**TCGA-EJ-A46D**	-853.177	-469.828	-1323.01	0.916808
	**TCGA-EJ-A7NN**	-478.919	138.9296	-339.989	0.849858
	**TCGA-EJ-A6RA**	-830.585	-240.752	-1071.34	0.901433
	**TCGA-HI-7168**	111.623	-703.039	-591.416	0.868725
	**TCGA-V1-A9O7**	189.0522	113.215	302.2672	0.796473
	**TCGA-V1-A9OA**	-28.8096	-154.854	-183.664	0.837542
	**TCGA-KK-A8IJ**	-857.301	-713.399	-1570.7	0.930718

	**TCGA-ZG-A9L1**	-411.759	-1117.71	-1529.47	0.928487
	**TCGA-KK-A7AW**	-790.743	728.4756	-62.2679	0.827673
	**TCGA-X4-A8KQ**	-159.986	4.579331	-155.407	0.835269
	**TCGA-HI-7171**	-631.959	-473.448	-1105.41	0.903587
	**TCGA-CH-5769**	-516.159	-248.283	-764.442	0.881023
	**TCGA-ZG-A9MC**	294.2652	1818.975	2113.24	0.609643
	**TCGA-YL-A9WL**	-958.817	-1001.96	-1960.77	0.950122
	**TCGA-ZG-A9ND**	-1126.79	-637.264	-1764.06	0.940722
	**TCGA-V1-A8WV**	-450.235	375.2891	-74.9463	0.828716
	**TCGA-KK-A59Y**	-1202.66	-695.985	-1898.64	0.947239
	**TCGA-ZG-A9N3**	-1013.41	451.7637	-561.643	0.866552
	**TCGA-J4-AATZ**	-1170.34	-682.806	-1853.15	0.945077
	**TCGA-ZG-A9LZ**	-535.999	96.67161	-439.328	0.857452
	**TCGA-EJ-5507**	-522.297	-2.45506	-524.753	0.863837
	**TCGA-XK-AAIV**	223.2745	1488.146	1711.421	0.65531
	**TCGA-EJ-5518**	209.1274	-191.63	17.4977	0.821046
	**TCGA-J9-A52E**	417.9517	231.9451	649.8968	0.764594
	**TCGA-KK-A8I7**	-84.6253	299.913	215.2876	0.804128
	**TCGA-VP-A87H**	-760.316	-488.145	-1248.46	0.912383
	**TCGA-YL-A9WX**	-398.578	235.239	-163.339	0.835908
	**TCGA-V1-A9Z7**	372.646	132.8969	505.5429	0.778079
	**TCGA-ZG-A9LM**	-85.181	-11.7191	-96.9001	0.830516
	**TCGA-KK-A6E8**	-1037.9	-839.724	-1877.62	0.946245
	**TCGA-YL-A8SJ**	58.11583	-729.445	-671.329	0.874475
	**TCGA-CH-5766**	-972.874	-65.3754	-1038.25	0.89932
	**TCGA-MG-AAMC**	-1251.49	-735.083	-1986.57	0.951296
	**TCGA-YL-A8HM**	-563.704	428.3018	-135.402	0.83365
	**TCGA-KK-A5A1**	-130.755	7.540743	-123.214	0.832661
	**TCGA-YL-A9WJ**	-170.593	203.5399	32.94697	0.819749
	**TCGA-KK-A8I8**	-797.556	-450.745	-1248.3	0.912374
	**TCGA-KK-A8IC**	-44.338	34.33792	-10	0.823343
	**TCGA-KK-A7B2**	-50.0052	1071.798	1021.793	0.728289
	**TCGA-YL-A9WI**	-717.255	-730.335	-1447.59	0.923957
	**TCGA-EJ-8469**	-770.723	-603.305	-1374.03	0.919773
	**TCGA-FC-A5OB**	-1165.01	-1011.15	-2176.15	0.959506
	**TCGA-V1-A9ZI**	16.48266	232.3742	248.8568	0.801189
	**TCGA-YL-A8SQ**	-464.403	-183.992	-648.396	0.872837
	**TCGA-CH-5741**	-1280.85	-864.663	-2145.52	0.958229
	**TCGA-ZG-A9LB**	-333.571	371.3482	37.77696	0.819343
	**TCGA-YL-A9WH**	-798.966	-563.447	-1362.41	0.919102
	**TCGA-2A-A8VT**	-984.114	-551.621	-1535.74	0.928828
	**TCGA-KK-A7B3**	-72.8797	473.2738	400.3941	0.787681
	**TCGA-G9-6499**	-1049.69	-291.811	-1341.5	0.917888

	**TCGA-YL-A8HL**	-1072.73	-1044.01	-2116.74	0.957013
	**TCGA-ZG-A9LY**	-272.769	-4.50468	-277.274	0.84497
	**TCGA-CH-5751**	126.565	1667.054	1793.619	0.646148
	**TCGA-KK-A8IA**	-975.477	-791.685	-1767.16	0.940876
	**TCGA-V1-A9ZG**	-122.958	-236.958	-359.917	0.851396
	**TCGA-EJ-A65F**	-204.53	-156.581	-361.111	0.851488
	**TCGA-VP-A878**	-184.708	10.46158	-174.247	0.836786
	**TCGA-2A-A8W3**	-675.956	-541.137	-1217.09	0.910489
	**TCGA-HC-A8CY**	-885.825	223.0378	-662.787	0.873866
	**TCGA-ZG-A9L6**	173.6327	147.5899	321.2226	0.794788
	**TCGA-ZG-A8QZ**	2.620654	308.4008	311.0214	0.795695
	**TCGA-HC-A9TE**	132.8602	1014.122	1146.982	0.715574
	**TCGA-G9-7521**	-8.38238	741.3864	733.004	0.756675
	**TCGA-YL-A9WK**	94.10289	140.8383	234.9412	0.80241
	**TCGA-YL-A8SI**	-109.942	32.3944	-77.5478	0.82893
	**TCGA-G9-A9S0**	-867.014	21.73835	-845.276	0.886574
	**TCGA-EJ-5506**	-963.392	-499.633	-1463.02	0.924821
	**TCGA-KK-A7B0**	-233.056	1050.009	816.9534	0.748561
	**TCGA-KK-A7AU**	-1810.53	-1132.44	-2942.97	0.985074
	**TCGA-HC-7821**	323.8894	376.2993	700.1887	0.759816
	**TCGA-V1-A9Z9**	-262.427	-374.169	-636.596	0.871991
	**TCGA-V1-A9OY**	-452.325	-12.7916	-465.117	0.859394
	**TCGA-EJ-5514**	136.2114	228.6841	364.8955	0.790881
	**TCGA-EJ-5519**	-535.088	-590.941	-1126.03	0.90488
	**TCGA-CH-5761**	-221.268	-553.137	-774.405	0.881714
	**TCGA-J9-A52B**	-410.783	-67.7338	-478.517	0.860398
	**TCGA-VN-A88K**	-250.553	-430.683	-681.236	0.875179
	**TCGA-HC-7213**	-437.301	-37.0515	-474.353	0.860086
	**TCGA-EJ-5495**	300.2322	339.6244	639.8566	0.765543
	**TCGA-KC-A4BR**	-35.7771	-287.658	-323.435	0.848575
	**TCGA-KK-A8IL**	-399.126	795.3983	396.2719	0.788054
	**TCGA-KK-A7B4**	-521.874	3.090221	-518.784	0.863395
	**TCGA-G9-6356**	-208.621	325.5268	116.9057	0.812629
	**TCGA-ZG-A9M4**	-694.415	841.383	146.9678	0.810049
	**TCGA-G9-6367**	-604.466	-394.021	-998.487	0.896752
	**TCGA-CH-5753**	-1065.35	-215.396	-1280.75	0.914313
	**TCGA-XK-AAIW**	-1308.02	92.92525	-1215.09	0.910367
	**TCGA-V1-A9Z8**	119.7725	98.52777	218.3002	0.803865
	**TCGA-G9-A9S4**	-1325.09	-898.829	-2223.92	0.961458
	**TCGA-YL-A8SB**	-27.4132	-159.413	-186.826	0.837796
	**TCGA-V1-A9O5**	-502.42	699.3811	196.9608	0.805724
	**TCGA-YL-A8HK**	-191.767	-70.6606	-262.427	0.843803
	**TCGA-EJ-A65J**	-564.289	-440.039	-1004.33	0.897132

	**TCGA-XQ-A8TB**	-95.51	-501.953	-597.463	0.869164
	**TCGA-CH-5788**	-908.11	-737.799	-1645.91	0.934698
	**TCGA-V1-A9OL**	-513.219	9.30917	-503.91	0.862291
	**TCGA-KK-A6E7**	-932.665	-605.561	-1538.23	0.928964
	**TCGA-VP-AA1N**	-204.536	230.6854	26.14966	0.82032
	**TCGA-YL-A8S9**	-1543.39	-1281.12	-2824.5	0.981932
	**TCGA-VP-A87D**	-1159.76	-984.941	-2144.7	0.958195
	**TCGA-KK-A8ID**	-1247.63	-671.34	-1918.97	0.948191
	**TCGA-VP-A879**	-609.341	-856.985	-1466.33	0.925005
	**TCGA-KC-A4BV**	-8.77689	88.28375	79.50686	0.815816
	**TCGA-EJ-5526**	-116.03	-82.8376	-198.867	0.838759
	**TCGA-KK-A6E6**	-185.747	-105.29	-291.037	0.846049
	**TCGA-YL-A8SC**	-362.898	269.5427	-93.3558	0.830226
	**TCGA-YL-A8SK**	-392.397	-18.743	-411.14	0.855316
	**TCGA-HC-A9TH**	-122.229	41.73185	-80.4974	0.829172
	**TCGA-ZG-A9L2**	616.2952	745.8949	1362.19	0.693154
	**TCGA-EJ-5524**	150.6974	217.3615	368.0589	0.790596
	**TCGA-EJ-A65B**	-266.644	-139.133	-405.777	0.854907
	**TCGA-ZG-A9L4**	229.5499	6.052289	235.6022	0.802352
	**TCGA-YL-A9WY**	366.2278	1172.915	1539.143	0.6742
	**TCGA-ZG-A9LU**	-776.405	417.0171	-359.388	0.851355
	**TCGA-V1-A8WW**	-546.086	-216.108	-762.194	0.880867
	**TCGA-ZG-A9NI**	183.923	-306.352	-122.429	0.832597
	**TCGA-KK-A59V**	-300.598	2169.161	1868.563	0.637713
	**TCGA-ZG-A9LS**	507.2379	1149.801	1657.039	0.661318
	**TCGA-CH-5772**	-463.46	-312.047	-775.507	0.88179
	**TCGA-YL-A8S8**	-307.824	-558.718	-866.542	0.888014
	**TCGA-CH-5754**	-217.37	724.1154	506.7453	0.777968
	**TCGA-M7-A723**	-21.513	803.6423	782.1294	0.751941
	**TCGA-J9-A8CM**	154.7002	-50.0273	104.6729	0.813674
	**TCGA-YL-A8SR**	-522.119	-587.241	-1109.36	0.903836
	**TCGA-YL-A8SL**	-1278.71	-955.839	-2234.54	0.961885
	**TCGA-EJ-A46F**	-1361.12	-871.469	-2232.59	0.961807
	**TCGA-EJ-5504**	67.94905	-301.129	-233.18	0.841491
	**TCGA-ZG-A9LN**	315.3209	1689.52	2004.841	0.622178
	**TCGA-EJ-5525**	405.4222	-470.714	-65.2922	0.827923
	**TCGA-KK-A8IK**	-1414.26	-857	-2271.26	0.963345
	**TCGA-G9-7523**	1047.437	783.2521	1830.689	0.641986
	**TCGA-KK-A6E0**	-1077.65	-608.816	-1686.47	0.936798
	**TCGA-KK-A8I4**	436.5492	1186.119	1622.669	0.665094
	**TCGA-CH-5792**	196.9577	770.8947	967.8524	0.733692
	**TCGA-KK-A6E1**	-1136.02	-613.302	-1749.32	0.939986
