# Supplementary material for: An Integrative Multi-Omics Analysis Based on Nomogram for Predicting Prostate Cancer Bone Metastasis Incidence
Source: Genet Res (Camb). 2022 Sep 29;2022:8213723. doi: 10.1155/2022/8213723 (PMC9537037; doi:10.1155/2022/8213723)
Supplement: Supplementary Materials — Figure S1: associated with Figure 2. Figure S2: associated with Figure 5. Figure S3: associated with Figure 6. Data 1-1: SEER data baseline characteristics of patient with prostate cancer; Data 1-2: train group ROC and validation ROC, Delong's test; Data 1-3: clinical features of TCGA samples and predictive results. Data 2: TMB scores of TCGA samples. Data 3: genes with different frequencies of CNVs between LRBM and HRBM cohorts. Data 4-1: DElncRNAs; Data 4-2: DEmiRNAs; Data 4-3: DEmRNAs. Data 5: gene module. Data 6-1: 29 immune microenvironment signatures; Data-2: ssGSEA score of immune microenvironment signature; Data 6-3: stromal score, immune score, estimate score, and tumor purity of TCGA samples. [file 8213723.f1.zip › 8213723.f1/Supplementary Figure (1).docx]

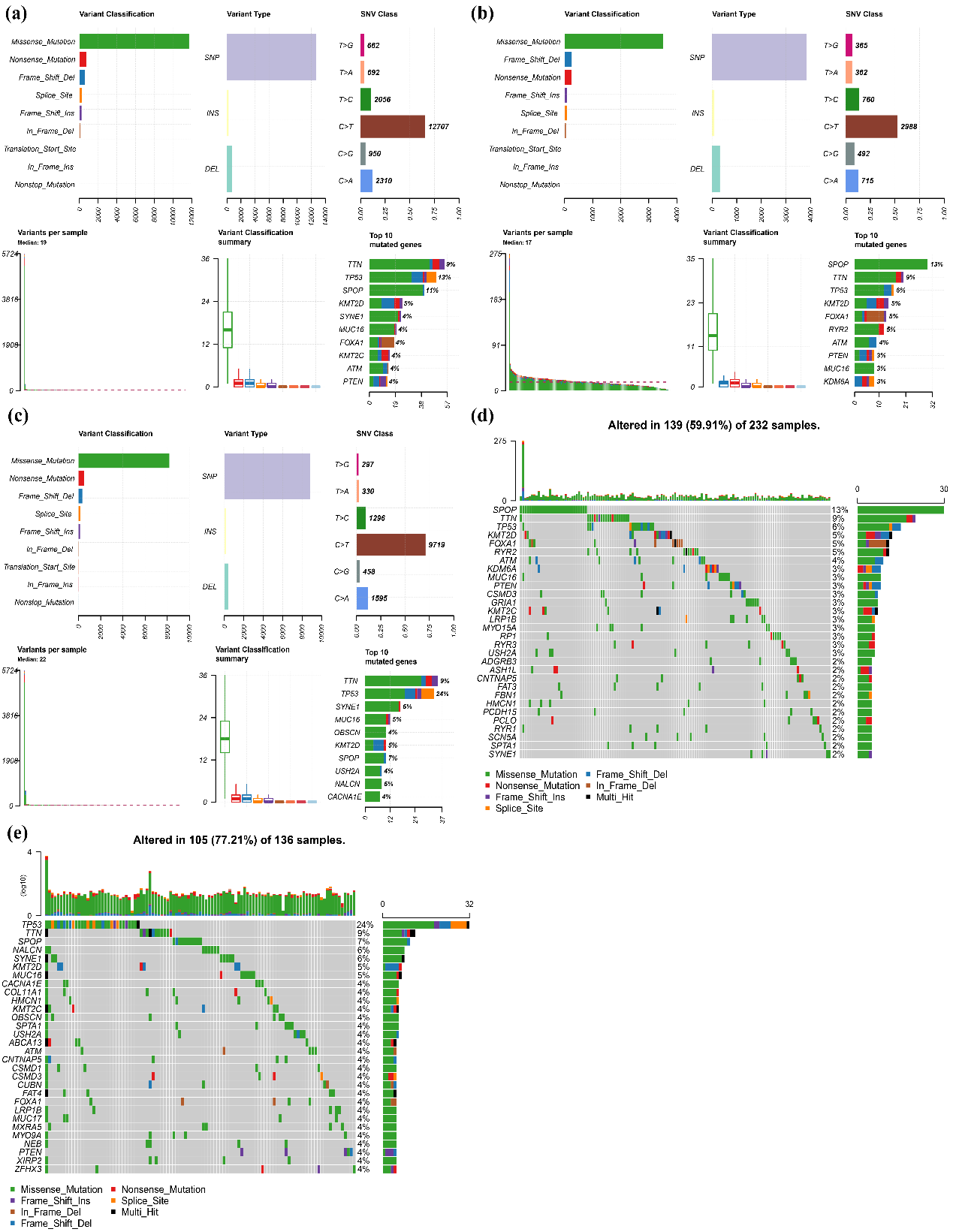


Figure S1: associated with Figure2. (a), Summary of the mutation information of all the 368 prostate cancer samples. (b), Summary of the mutation information of the LRBM cohort. (c), Summary of the mutation information of the HRBM cohort. (a,b,c), Number of variants in each sample as a stacked barplot and variant types as a boxplot summarized by Variant_Classification. (d), Landscape of mutation profiles in LRBM cohort. (e), Landscape of mutation profiles in HRBM cohort. (d,e), Mutation information of each gene in each sample was shown in the waterfall plot, where different colors with specific annotations at the bottom mean the various mutation types. The barplot above the legend exhibited the mutation burden.


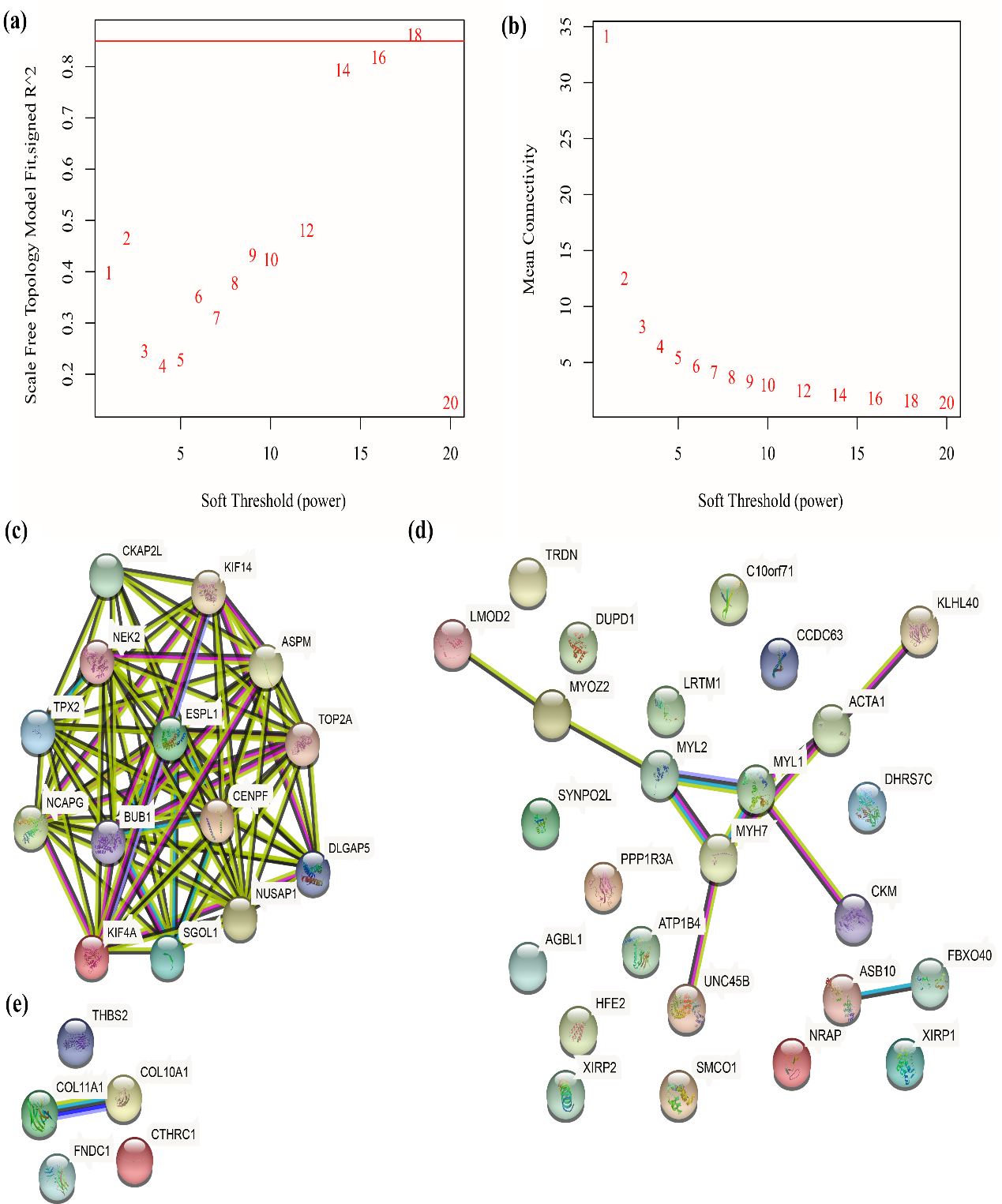


Figure S2: associated with Figure5. (a), The influence of soft-thresholding power (x-axis) on scale-free fit index (y-axis). Horizontal lines represent scale-free fit index (y-axis) of 0.85. (b), The influence of soft-thresholding power (x-axis) on the mean connectivity (degree, y-axis). (c), PPI network of blue module genes. (d), PPI network of turquoise module genes. (e), PPI network of brown module genes.


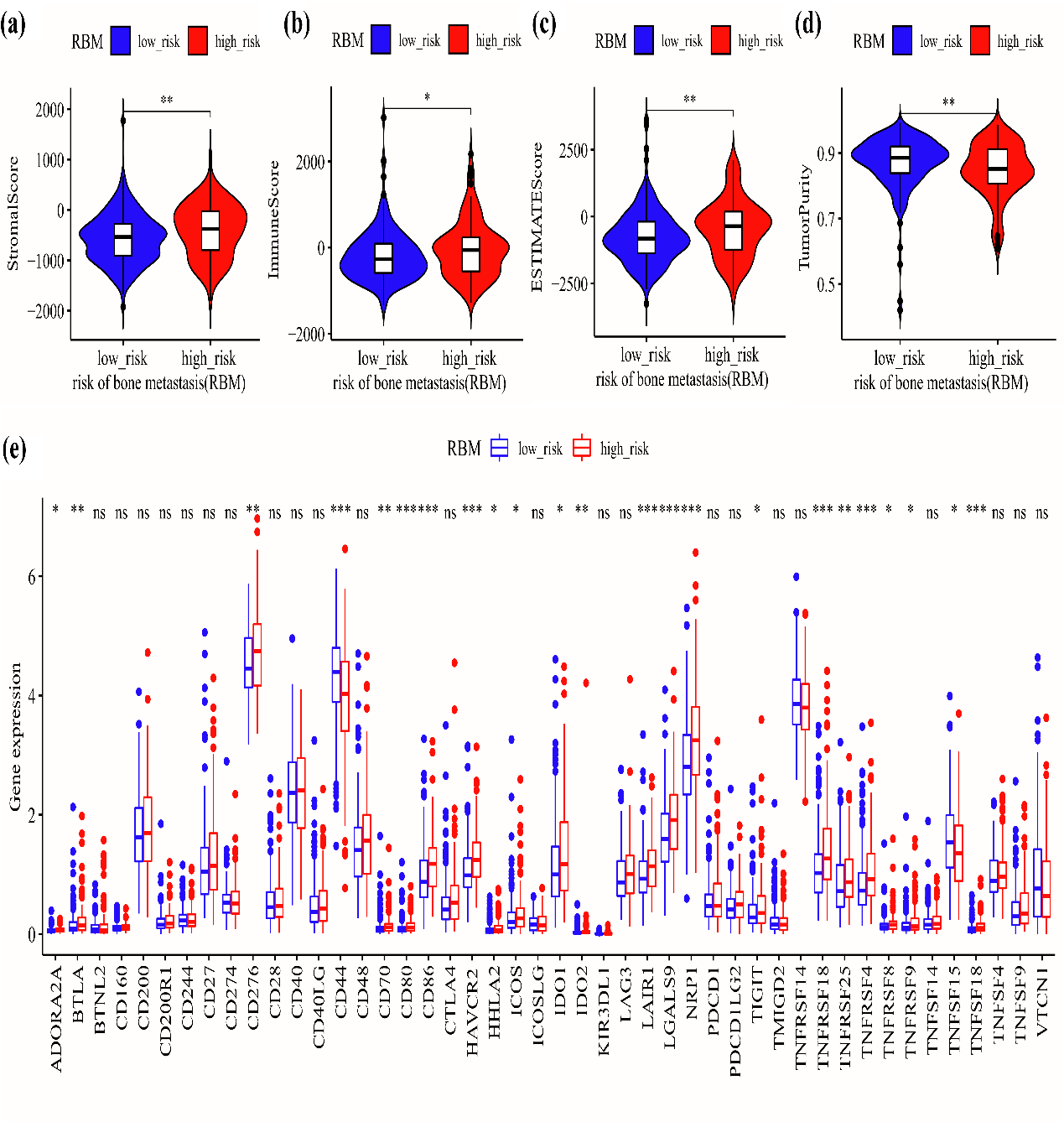


Figure S3: associated with Figure6. (a), Comparison of stromal scores between LRBM and HRBM cohort. (b), Comparison of immune scores between LRBM and HRBM cohort. (c), Comparison of ESTIMATE scores between LRBM and HRBM cohort. (d), Comparison of tumor purity between LRBM and HRBM cohort. (e), Comparison of immune check point genes between LRBM and HRBM cohort. (a, b, c, d, e), p values were determined by two-sided Mann-Whitney U test. The level of significance is indicated by *p<0.05, **p<0.01, ***p<0.001.
